# Ebselen analogues with dual human neutrophil elastase (HNE) inhibitory and antiradical activity†

**DOI:** 10.1039/d3md00736g

**Published:** 2024-03-19

**Authors:** Letizia Crocetti, Francesca Catarzi, Maria Paola Giovannoni, Claudia Vergelli, Gianluca Bartolucci, Marco Pallecchi, Paola Paoli, Patrizia Rossi, Martina Lippi, Igor A. Schepetkin, Mark T. Quinn, Gabriella Guerrini

**Affiliations:** a NEUROFARBA, Pharmaceutical and Nutraceutical Section, University of Florence Via Ugo Schiff 6 50019 Sesto Fiorentino Italy letizia.crocetti@unifi.it +39 055 4573683; b Department of Industrial Engineering, University of Florence Via Santa Marta 3 50139 Florence Italy; c Department of Microbiology and Cell Biology, Montana State University Bozeman MT 59717 USA

## Abstract

Human neutrophil elastase (HNE) plays an essential role in host defense against bacteria but is also involved in several respiratory diseases. Recent reports suggest that compounds exhibiting a combination of HNE inhibitory activity with antiradical properties may be therapeutically beneficial for the treatment of respiratory diseases involving inflammation and oxidative stress. We report here the synthesis and biological evaluation of novel ebselen analogues exhibiting HNE inhibitory and antiradical activities. HNE inhibition was evaluated in an enzymatic system using human HNE, whereas antiradical activity was evaluated in a cell-based assay system using phorbol 12-myristate 13-acetate (PMA)-stimulated murine bone marrow leukocytes as the source of reactive oxygen species (ROS). HNE inhibition was due to the N–CO group targeting Ser195-OH at position 2 of the scaffold, while antiradical activity was due to the presence of the selenium atom. The most active compounds 4d, 4f, and 4j exhibited a good balance between anti-HNE (IC_50_ = 0.9–1.4 μM) and antiradical activity (IC_50_ = 0.05–0.7 μM). Additionally, the solid-state structure of 4d was determined and compared to that of the similar compound *N*-propionyl-1,2-benzisoselenazol-3(2*H*)-one.

## Abstract 2

HNE inhibitors with antiradical properties may be therapeutically beneficial for the treatment of respiratory diseases involving inflammation and oxidative stress.

## Graphical abstract

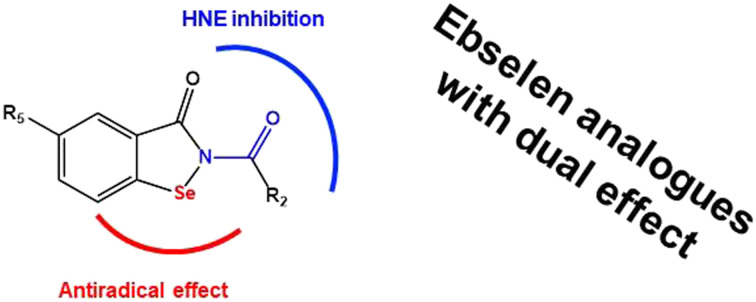

## Introduction

**Fig. 1:**
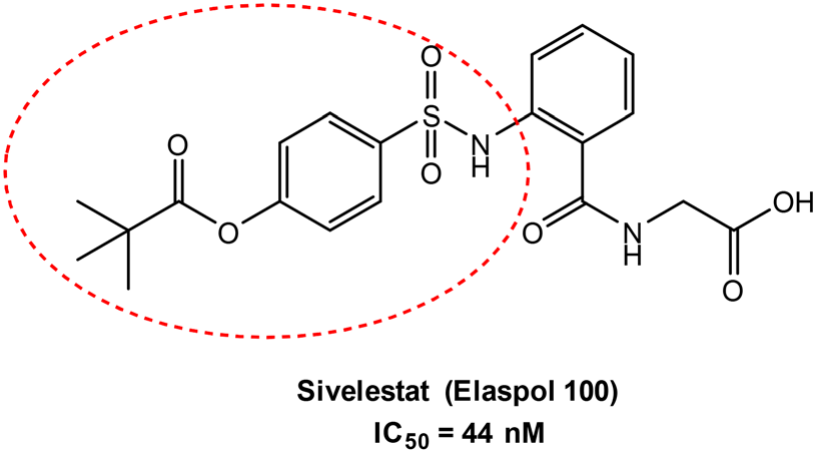
Sivelestat from Ono Pharma.

Human neutrophil elastase (HNE) is a serine protease that catalyzes its proteolytic activity through the use of a catalytic triad comprised of Ser195-Asp102-His57. The OH of Ser195 is responsible for nucleophilic attack at the carbonyl carbon of the peptide bond in the substrate being hydrolyzed.^1,2^ HNE is important in host defense against bacteria but is also involved in several respiratory diseases due to its aberrant elastolytic activity in the lung, including chronic obstructive pulmonary disease (COPD), acute respiratory distress syndrome (ARDS), acute lung injury (ALI), and cystic fibrosis.^3,4^ Recently, it has been reported that excessive HNE elastolytic activity in the lungs of Covid-19 patients is responsible for disease exacerbation and the associated severe respiratory complications.^5^ There are currently only two HNE inhibitors on the market, the “small molecule” sivelestat^6^ (Fig. 1) used for the treatment of ALI associated with systemic inflammatory response syndrome (SIRS) and the peptide Prolastin® (purified α1-AT), which is in use for a1-antitrypsin deficiency (AATD).^7^ However, sivelestat is also in clinical trials as a preventive therapy against Covid-19-induced ARDS in lymphocytopenic patients who have not yet developed abnormal levels of neutrophils and NETs.^8^

**Fig. 2:**
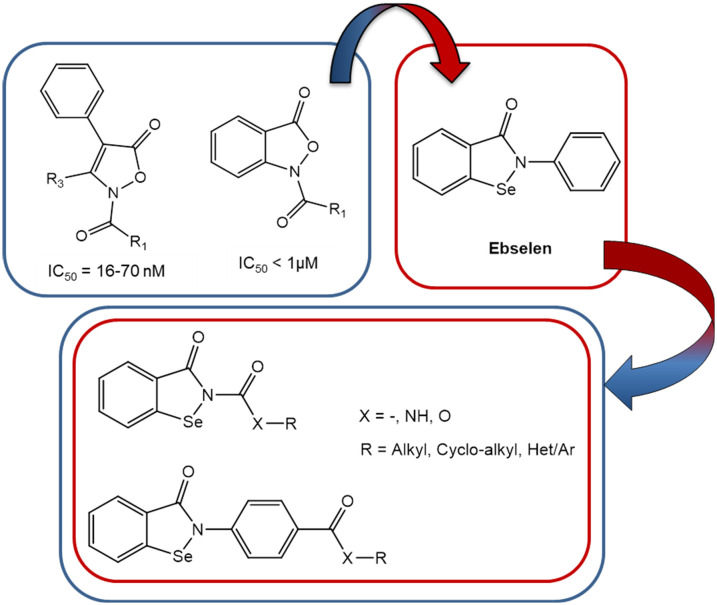
Developing dual HNE inhibitor/ebselen analogues.

The interest of the medicinal chemists in selenium-containing compounds has grown considerably in recent years^9,10^ due to the chemical–physical properties of this element and its numerous beneficial physiological effects.^11^ Specifically, selenium-containing compounds are of significant interest because of their antiradical/antioxidant activity, as oxidative stress plays a crucial role in the development of a number of human diseases.^12^ Notably, ebselen (2-phenyl-1,2-benzoselenazol-3(2*H*)-one) (Fig. 2), one of the first selenium-containing compounds synthesized,^13^ exhibits potent antioxidant activity and is currently under clinical investigation for treatment of a number of disorders.^9,14,15^

In 2020, Laforge and co-workers^16^ published an interesting study suggesting that compounds with synergistic HNE inhibitory and antioxidant activities could have therapeutic potential for the treatment of Covid-19. Based on our previous experience in developing potent HNE inhibitors,^17,18^ we initiated a new strategy to synthesize novel ebselen-containing seleno derivatives of our previously reported HNE inhibitors (Fig. 2). The aim was to obtain compounds dually endowed with HNE inhibitory and antiradical activities due to the selenium atom. These selenium-containing compounds contained an N–CO group, which we found previously to be responsible for HNE inhibitory activity of all our published compounds, as well as those groups/fragments that gave the best HNE-inhibition in the previous compound series.^19–21^ We also investigated the effects of adding other groups, such as urea and carbamate, and a fragment of the drug sivelestat (4-(sulfamoyl)phenyl pivalate), resulting in the first small series of seleno derivatives (red circle in Fig. 1). HNE inhibition and antiradical activity of all new compounds were evaluated to assess their therapeutic potential.

## Results and discussion

### Chemistry

**Scheme 1:**
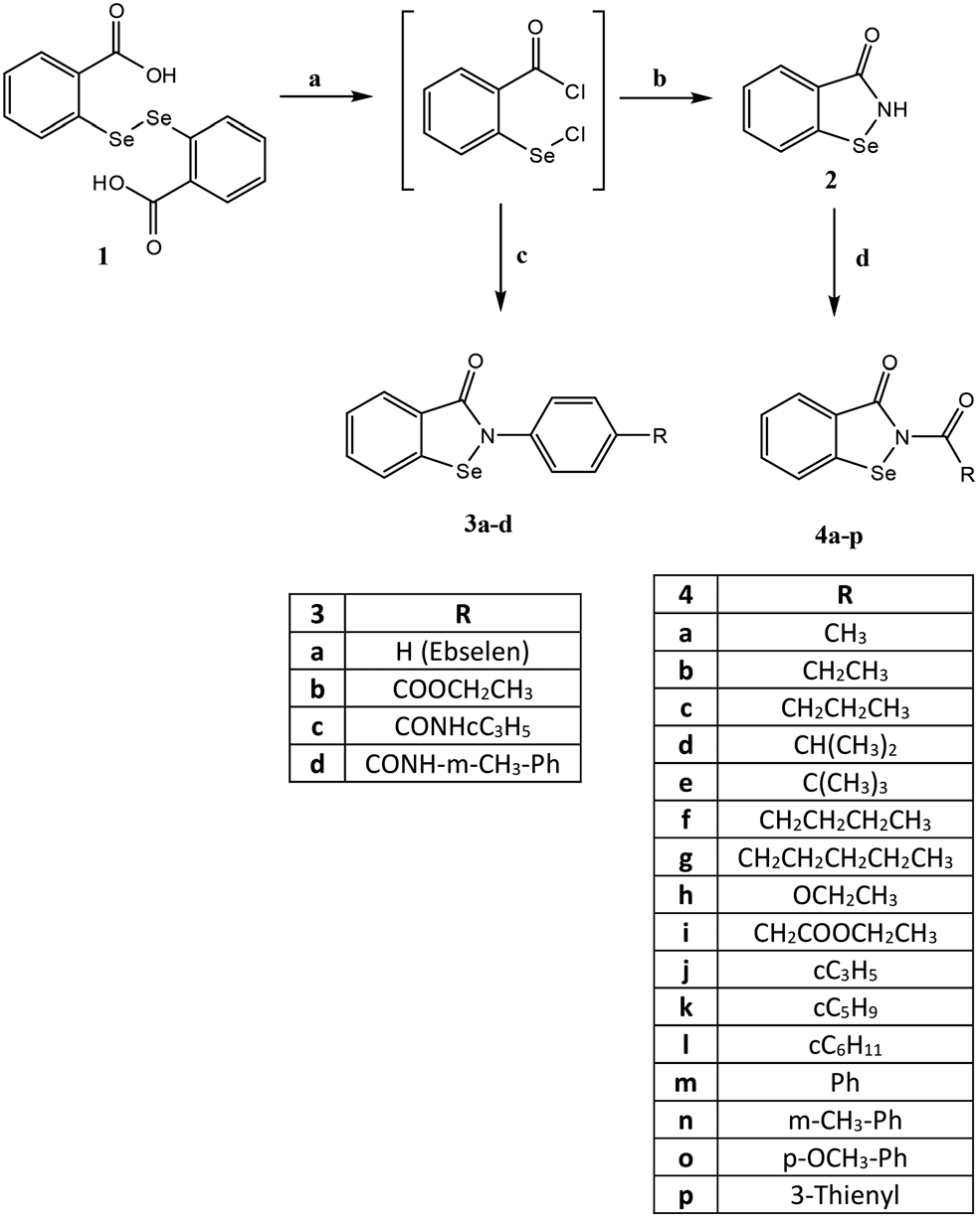
Reagents and conditions: a) SOCl_2_ (excess), reflux, 5–6 h; b) NH_3_ (g), dry THF, 1 h; c) appropriate R-aniline, Et_3_N, anhydrous CH_2_Cl_2_, 2 h, r.t.; d) R–COCl, Et_3_N, anhydrous CH_2_Cl_2_, 0 °C, 2 h, then r.t., 2 h. For 4m: benzoyl chloride, NaH, dry THF, 2 h. For 4p: 3-thiophene carboxylic acid, SOCl_2_, anhydrous toluene, reflux, 2 h then NEt_3_, anhydrous toluene, reflux, 6 h.

**Scheme 2:**
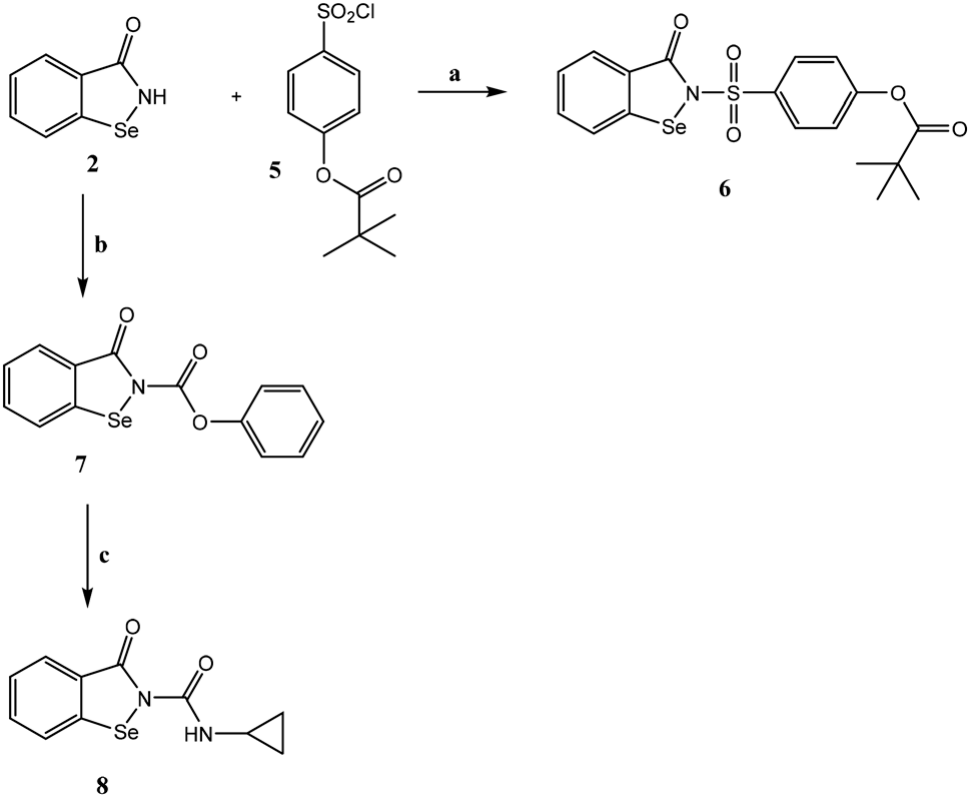
Reagents and conditions: a) NaH, dry THF, 6 h, r.t.; b) phenyl chloroformate, DIPEA, dry CH_3_CN, 3 h, r.t.; c) cyclopropylamine, DIPEA, dry CH_3_CN, reflux, N_2_, 3 h.

The compounds were synthesized as reported in Schemes 1 and 2, and the structures were confirmed on the basis of analytical and spectral data. All final compounds were characterized by ^1^H-NMR, ^13^C-NMR, and ITMS (ESI) to confirm structures and purity, with most exhibiting purity >95%. In addition, the ^77^Se-NMR spectrum was determined for representative compounds. Scheme 1 shows the synthetic pathway used to obtain ebselen-containing compounds of type 3 and the *N*-acyl derivatives of type 4. By treating the benzoic acid diselenide 1^22^ with an excess of thionyl chloride in toluene at reflux, the acyl chloride (not isolated) was obtained. The latter was treated with the suitable/appropriate aniline (synthesized by us or commercially available) in anhydrous dichloromethane and triethylamine to obtain the final compounds 3a–d (3a^23^ and 3b^24^). Compound 3a (R = H) is ebselen and was synthesized for biological assays as a reference compound. On the other hand, the treatment of the acyl chloride with NH_3_ gas in tetrahydrofuran led to the key intermediate benzo[*d*][1,2]selenazol-3(2*H*)-one 2,^25^ which was acylated using the appropriate acyl chloride in dichloromethane and triethylamine to obtain the final compounds of type 4. For compounds 4m and 4p, it was necessary to follow different procedures. For synthesis of compound 4m, the reaction was performed with sodium hydride in dry tetrahydrofuran. For synthesis of compound 4p, the acyl chloride was synthesized from 3-thiophene carboxylic acid and thionyl chloride and then reacted with intermediate 2 in toluene and triethylamine at reflux.

Scheme 2 shows synthesis of the final product 6, which has a portion of the drug sivelestat in its structure, and of the ureido derivative compound 8. The sulfonyl chloride 5, synthesized as reported in the literature,^26^ was reacted with intermediate 2 in the presence of sodium hydride in dry THF, resulting in the final compound 6. To obtain compound 8, intermediate 2 was treated with phenyl chloroformate and DIPEA in anhydrous CH_3_CN, resulting in the carbamate 7, which was reacted with cyclopropylamine and DIPEA in anhydrous CH_3_CN at reflux under nitrogen to obtain the final ureido derivative 8.

### Single-crystal X-ray diffraction

**Fig. 3:**
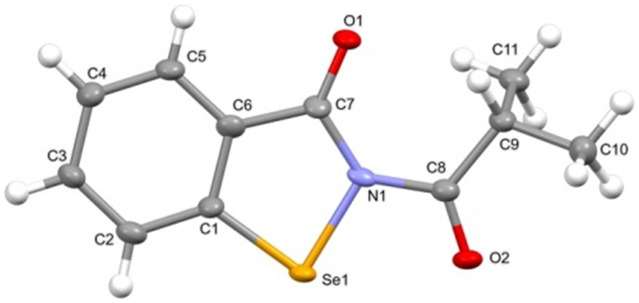
Ortep view of the asymmetric units of 4d (ellipsoid probability = 30%).

**Fig. 4:**
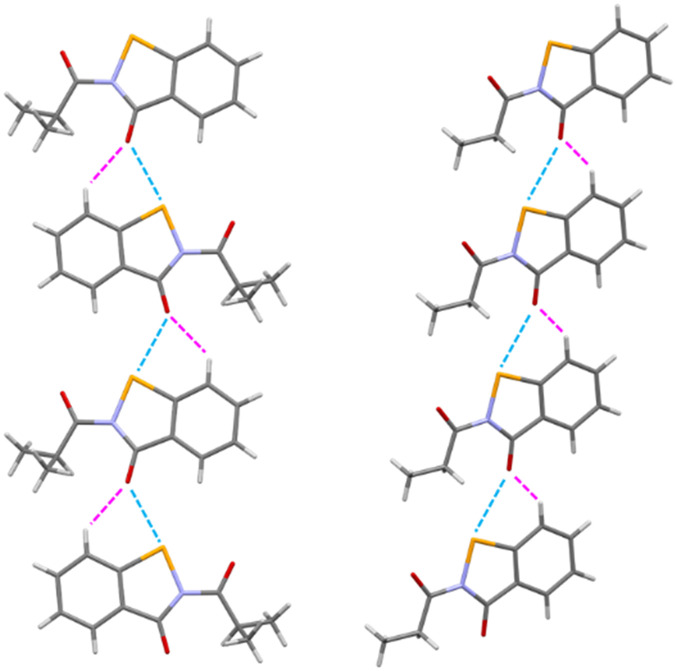
Chain in 4d (left) and XAYHIO (right) propagating along the *a*-axis.

In the asymmetric unit of 4d, a molecule of 4d is present. All of the non-hydrogen atoms, with the exception of C10 and C11, lie on a plane, with the maximum deviation from the mean plane defined by all the non-hydrogen atoms (with the exception of C10 and C11) is due to C9 (0.092(6) Å). These two remaining carbon atoms were located on the opposite end of this plane. All of the bond distances are in agreement with those observed in similar compounds, and the narrow C1–Se1–N1 angle (85.0(2)°) compares well with the values found in molecules containing a selenazole ring. A search performed using the Cambridge structural Database (CSD, v 5.42 update)^27^ allowed us to retrieve a compound very similar to 4d (CSD Refcode = XAYHIO).^28^ In XAYHIO, the isopropyl group present in 4d is substituted with an ethyl group. The structural conformation of the two molecules is quite comparable (see Fig. S1†), and they show a similar crystal packing. In fact, adjacent molecules interact in both compounds *via* chalcogen bonds^29^ involving the selenium and the endocyclic carbonyl oxygen atoms of two adjacent molecules; however, the Se⋯O distance observed in 4d (2.853(4) Å) is significantly shorter than that observed in XAYHIO (3.189(4) Å). A search performed in the CSD showed that this kind of interaction is common in molecules derived from the 1,2-benzisoselenazol-3(2*H*)-one, where the Se⋯O distance is seen in the 2.52–3.38 Å range (mean value = 2.87 Å). For example, the Se⋯O distance is 2.571 in the crystal structure of ebselen, CSD Refcode = SENGOH.^30^ In addition, the 4d molecules (as well as the XAYHIO molecule) interact *via* a CH⋯O bond involving the hydrogen atom bonded to C2 and the oxygen atom O1 (see Fig. 3, C2⋯O1 distance = 3.203(7) Å, H2⋯O distance = 2.49(7) Å, C2–H2⋯O1 angle 136(6)°). A similar interaction is observed in XAYHIO. In contrast, the resulting chain has a zig-zag evolution in 4d (see Fig. 4).

### Biological evaluation

**Fig. 5:**
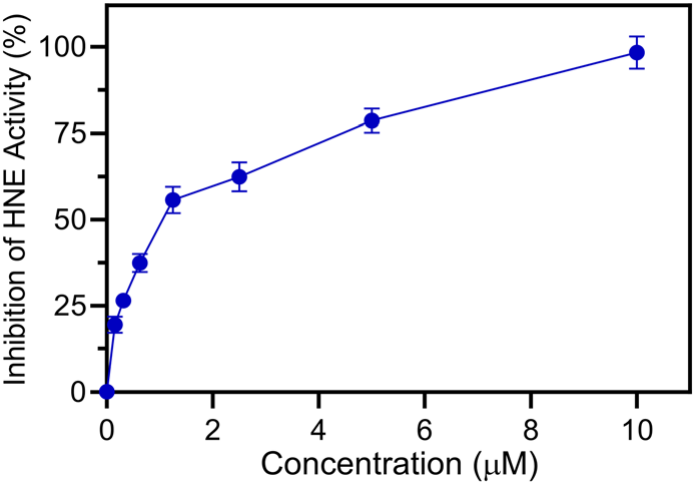
Inhibition of HNE by compound 4f. HNE was incubated with the indicated concentrations of 4f, and cleavage of the fluorogenic HNE substrate was monitored, as described. The percent inhibition of HNE activity is plotted *vs.* the inhibitor concentration. The data are presented as the mean S.D. of triplicate samples from a representative experiment of three independent experiments.

The HNE inhibitory activity and the antiradical activities of all compounds were evaluated and compared with activities of the reference drug sivelestat for HNE inhibition and ebselen for antiradical effects. Although ebselen has not been previously evaluated as an HNE inhibitor, it was found to be completely inactive. Most compounds exhibited some HNE inhibitory activity, with values in the micromolar range (4a–g, 4j–o) (Table 1). As an example, a representative dose–response curve showing HNE inhibitory activity for compound 4f is shown in Fig. 5. As expected, all active compounds were characterised by the presence of the N–CO group at position 2 of the benzo[*d*][1,2]selenazol-3(2*H*)-one nucleus. On the other hand, compounds in which the nitrogen at position 2 was contained in different groups, such as urea (8) or carbamate (4h), or those products containing an amide or ester group in the *para* position of the phenyl (3b–d), appeared to be completely inactive, indicating that the endocyclic N–CO function was essential for anti-HNE activity in this new series of compounds, which confirms our previous observations regarding the importance of this group for HNE inhibitory activity.^17,20,21^ Thus, we conclude that attack of the OH group of Ser195 occurs *via* the N–CO function at position 2 for these new compounds as well. Compounds 4a–g, which contain an alkyl group linked to the amide function also exhibited good HNE inhibitory activity, with IC_50_ values in the low micromolar range (0.9–3.0 μM).

**Table 1:** HNE inhibitory activity in an enzymatic assay, antiradical effects in a cell-based assay of ROS production, and cytotoxicity of new Se-derivatives 3a–d, 4a–p, 6 and 8

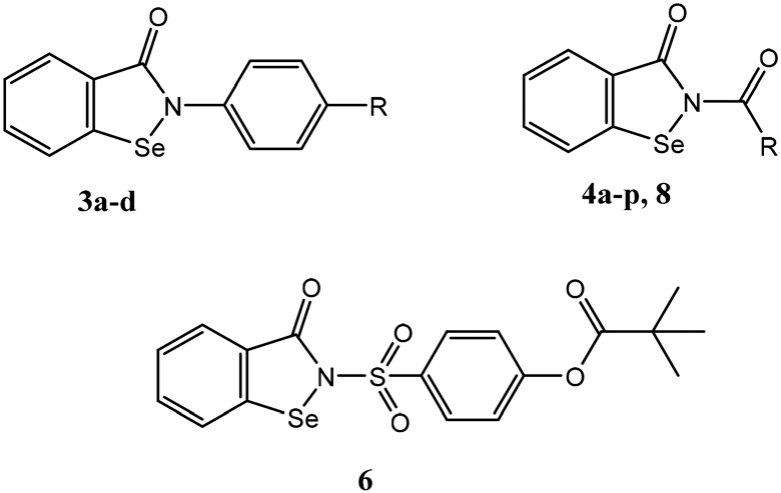				
Comp.	R	HNE inhibition	Antiradical activity	Cyto-toxicity
		IC_50_^a^ (μM)		
3b	COOCH_2_CH_3_	N.A.^b^	1.8 ± 0.2	N.A.^b^
3c	CONH–*c*C_3_H_5_	N.A.^b^	9.7 ± 1.4	8.0 ± 1.9
3d	CONH-3-CH_3_-Ph	N.A.^b^	N.A.^b^	N.A.^b^
4a	CH_3_	7.7 ± 2.3	0.42 ± 0.06	10.8 ± 2.4
4b	C_2_H_5_	2.6 ± 0.2	0.18 ± 0.05	8.5 ± 1.8
4c	CH_2_CH_2_CH_3_	2.4 ± 0.4	0.33 ± 0.08	4.9 ± 0.6
4d	iC_3_H_7_	1.2 ± 0.1	0.60 ± 0.2	9.6 ± 2.2
4e	C(CH_3_)_3_	1.8 ± 0.1	2.6 ± 0.4	13.6 ± 3.1
4f	*n*C_4_H_9_	0.9 ± 0.2	0.70 ± 0.2	4.2 ± 0.9
4g	*n*C_5_H_11_	3.0 ± 0.4	N.A.^b^	N.A.^b^
4h	OC_2_H_5_	N.A.^b^	2.5 ± 0.4	15.2 ± 3.2
4i	CH_2_COOCH_2_CH_3_	N.A.^b^	1.0 ± 0.3	8.1 ± 1.7
4j	*c*C_3_H_5_	1.4 ± 0.1	0.05 ± 0.01	10.4 ± 2.4
4k	*c*C_5_H_9_	2.8 ± 0.2	1.4 ± 0.2	11.8 ± 2.8
4l	*c*C_6_H_11_	24.1 ± 4.8	1.1 ± 0.1	12.4 ± 3.4
4m	Ph	2.3 ± 0.4	N.T.^c^	N.T.^c^
4n	3-CH_3_-Ph	10.9 ± 2.1	7.0 ± 1.3	N.A.^b^
4o	4-OCH_3_-Ph	8.7 ± 1.2	N.T.^c^	N.T.^c^
4p	3-Thienyl	N.A.^b^	14.3 ± 2.7	N.A.^b^
8	NH–*c*C_3_H_5_	N.A.^b^	0.90 ± 0.3	5.9 ± 1.6
6	—	2.7 ± 0.5	6.4 ± 0.4	N.A.^b^
**Ebselen** (3a)	H	N.A.^b^	0.15 ± 0.02	9.5 ± 2.5
**Sivelestat**	—	0.044	—	N.A.^b^

a IC_50_ values are presented as the mean ± SD of three independent experiments.

b N.A.: no inhibitory activity was found at the highest concentration of compound tested (50 μM) or no cytotoxicity activity was found at the highest concentration of compound tested (25 μM).

c N.T.: not tested.

Overall, our biological results demonstrate that there is an optimal hindrance and length of the alkyl group for HNE inhibitory activity. In fact, an increase in activity was observed moving from R = methyl (4a) to R = *n*-pentyl (4g) and passing through the branched isopropyl (4d) and *tert*-butyl (4e) groups until arriving at the optimal *n*-butyl derivative 4f, which was the most potent compound, with IC_50_ = 0.9 μM. On the other hand, further lengthening of the chain, as in 4g (R = *n*-pentyl), resulted in a decrease in activity (IC_50_ = 3.0 μM).

The importance of the hindrance of the alkyl group was also confirmed by comparing activities of the cycloalkyl derivatives 4j, 4k and 4l (R = *c*C_3_H_5_, IC_50_ = 1.4 μM; R = *c*C_5_H_9_, IC_50_ = 2.8 μM and R = *c*C_6_H_11_, IC_50_ = 24.1 μM, respectively). Indeed, the cyclopropyl derivative 4j exhibited activity comparable to that of isopropyl derivative 4d and the *t*-butyl derivative 4e, whereas the cyclopentyl 4k had reduced activity similar to that of *n*-pentyl 4g. Measurements of surface accessible to solvent (Connolly surface, Å^2^) and the subtended volume (Å^3^) using the program Accelrys/DSViewerPro60 demonstrated similar values of the alkyl substituent for compounds 4j, 4d, and 4e on the one hand and for compounds 4k and 4g on the other, thus confirming our interpretation of the structure–activity data (see Table S3, Fig. S2–S5 in ESI†). In further support of this conclusion, enlargement of the cycle further decreased activity (4l, R = *c*C_6_H_11_, IC_50_ = 24.1 μM).

HNE inhibitory activities of the few compounds in which a (hetero)aromatic substituent (*i.e.*, phenyl, R-phenyl and 3-thienyl) was introduced at N-2 (compounds 4m–p) seem, as a first hypothesis, to support the importance of the substituent's bulk, but need confirmation with the synthesis of additional compounds.

In compound 6, which contains the pharmacophore of sivelestat on N-2 of the benzo[*d*][1,2]selenazol-3(2*H*)-ion scaffold, we observed the maintenance of HNE inhibitory activity (IC_50_ = 2.7 μM), which was comparable to most other compounds of this series. This finding is in agreement with previously published results, in which the point of attack of the Ser195 OH of HNE is the terminal ester group of sivelestat.^19^

**Fig. 6:**
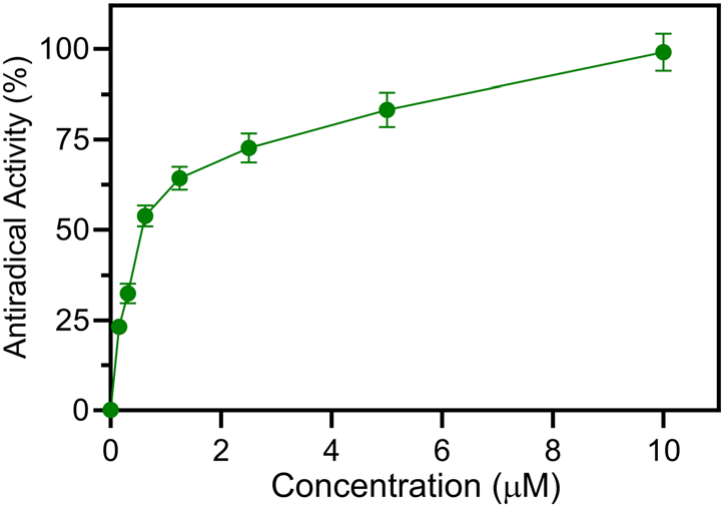
Inhibition of ROS production in PMA-stimulated murine bone marrow neutrophils by compound 4f. ROS production was monitored as L-012-enhanced chemiluminescence, as described. The percent inhibition of the luminescence signal is plotted *vs.* the inhibitor concentration. The data are presented as the mean S.D. of triplicate samples from a representative experiment of three independent experiments.

The phagocyte respiratory burst generates reactive oxygen species (ROS) through activation of the NADPH oxidase enzyme, and both receptor-mediated and receptor-independent processes can activate this oxidase.^31,32^ Phorbol-12-myristate 13-acetate (PMA) has been used extensively to activate the phagocyte receptor-independent respiratory burst^33,34^ and was used here to activate leukocyte ROS production. Thus, to assess antiradical activities of new Se-derivatives 3b–d, 4a–p, 6, and 8 and control ebselen (3a), we evaluated the ability of test compounds to scavenge reactive oxygen species (ROS) produced by PMA-activated murine bone marrow leukocytes (see Table 1). ROS production was significantly reduced by most of the new compounds tested, with compounds 4a–d, 4f, and 4j having significant antiradical activity (IC_50_ < 1 μM). As an example, a representative curve showing dose dependent antiradical for compound 4f is shown in Fig. 6. Notably, the most active compound 4j was more potent toward HNE than ebselen itself (IC_50_ = 50 nM for 4j*versus* IC_50_ = 150 nM for ebselen). The mechanism by which these new compounds exert their antiradical effect is under investigation, but a mechanism similar to that of ebselen can plausibly be assumed, since all compounds were ebselen analogues. For this reason, the inactivity of 3d and 4g cannot be explained. Nevertheless, of the new compounds have dual activity, acting as HNE inhibitors with micromolar values (IC_50_ = 0.9–3.0 μM) and ROS scavengers with activity in the submicromolar range (IC_50_ = 50–600 nM).

To evaluate cytotoxicity of the test compounds, murine bone marrow leukocytes were incubated with the ebselen analogs, ebselen, and sivelestat at various concentrations (up to 25 μM) for 90 min and leukocyte viability was evaluated. Compounds 3b, 3d, 4g, 4n, 4p, 6, and sivelestat had no cytotoxicity during a 90 min incubation period, although ebselen and other analogs were cytotoxic with IC_50_ from 4.2 to 15.2 μM (Table 1). This was not surprising, as ebselen has been shown to exhibit cytotoxic activity against many cell lines and can induce apoptosis through different mechanisms, including thiol depletion, mitochondria dysfunction and cytochrome C release.^35–38^ While cytotoxicity occurred at much higher concentrations of the ebselen analogs than those needed to inhibit HNE or exhibit antiradical activity, further modification of these compounds will be necessary to reduce cytotoxicity if they ever could become useful therapeutically. Additionally, further experiments are needed to investigate pro-apoptotic mechanisms and anticancer effects of these novel ebselen analogs.

## Experimental

### Chemistry

All melting points were determined on a Büchi apparatus (New Castle, DE, USA) and are uncorrected. Extracts were dried over Na_2_SO_4_, and the solvents were removed under reduced pressure. Merck F-254 commercial plates (Merck, Durham, NC, USA) were used for analytical TLC to follow the course of reactions. Silica gel 60 (Merck 70–230 mesh, Merck, Durham, NC, USA) was used for column chromatography. ^1^H-NMR, ^13^C-NMR, and ^77^Se-NMR spectra were recorded on an Avance 400 instrument (Bruker Biospin Version 002 with SGU, Bruker Inc., Billerica, MA, USA). Chemical shifts (*δ*) are in parts per million (ppm) approximated by the nearest 0.01 ppm, using the solvent as internal standard. Coupling constants (*J*) are in Hz; they were calculated by Top Spin 3.1 and approximated by 0.1 Hz. Data are reported as follows: chemical shift, multiplicity (exch, exchange; br, broad; s, singlet; d, doublet; t, triplet; q, quartet; m, multiplet; or a combination of those, *e.g.*: dd), integral, assignments, and coupling constant. All new compounds had a purity >95%; microanalyses indicated by symbols of the elements were performed on a Perkin-Elmer 260 elemental analyzer for C, H, and N, and they were within ±0.4% of the theoretical values. The mass spectrometry analysis was performed by analyzing the compounds in study by a Thermo LTQ (Linear Ion Trap, LIT), coupled with a HPLC Dionex Ultimate 3000 through an ElectroSpray (ESI) source (Waltham, MA). The chromatography program was the same as the one used for the evaluation of purity. The ion trap worked in scan mode recording a range of *m*/*z* from 150 to 750 in positive polarity. Samples were prepared at 5 μg mL^−1^ of concentration in methanol while the injection volume was 5 μL. Purity was evaluated employing an Agilent 1200 HPLC-DAD (Palo Alto, CA). The solvents used are the following: a) solvent A: H_2_O : CH_3_CN 90 : 10, 10 mM HCOOH and 5 Mm HCOONH_4_; b) solvent B: H_2_O : CH_3_CN 10 : 90, 10 mM HCOOH and 5 Mm HCOONH_4_. The elution gradient was developed as follows: starting from 90% of solvent A and reaching 10% of solvent A in 8 min (10% min^−1^), then kept at 10% of solvent A for 5 min, then the initial conditions were restored in 0.1 min for a total run time of 20 minutes. The flow was kept at 0.25 ml min^−1^ and the injection volume was 5 μL. The column used was an Agilent Pursuit XRs C18 50 × 2 mm, 3 μ. The wavelength used to evaluate the purity was 240 nm because it was common for all the compounds in study and the most intense. Samples were prepared at 10 μg mL^−1^ of concentration in methanol.

#### General procedure for compounds 3c, d

2,2′-Diselanediyldibenzoic acid 1^22^ (2.03 mmol) was dissolved in an excess of SOCl_2_ (6 mL) and refluxed for 5 h. After cooling, excess SOCl_2_ was removed *in vacuo*. The crude compound was dissolved in 6 mL of dry dichloromethane, and a previously prepared solution composed of 4-amino-*N*-cyclopropylbenzamide^39^ (for 3c) or 4-amino-*N*-(*m*-tolyl)benzamide^40^ (for 3d) (0.97 mmol) and Et_3_N (1.94 mmol) in 10 mL of dry dichloromethane was dripped in at 0 °C. The mixture was stirred at room temperature for 2 h, the precipitate was removed by filtration, and the organic solvent was recovered and evaporated under vacuum. The crude compound was purified by flash column chromatography, using dichloromethane/methanol 95 : 5 as the eluent.

#### *N*-Cyclopropyl-4-(3-oxobenzo[*d*][1,2]selenazol-2(3*H*)-yl)benzamide (3c)

Yield = 15%; mp = 228–231 °C (EtOH). ^1^H-NMR (400 MHz, DMSO-d_6_) *δ* 0.55–0.60 (m, 2H, CH_2_*c*C_3_H_5_); 0.65–0.70 (m, 2H, CH_2_*c*C_3_H_5_); 2.81–2.86 (m, 1H, CH *c*C_3_H_5_); 7.46 (t, 1H, Ar, *J* = 7.6 Hz); 7.67 (t, 1H, Ar, *J* = 7.6 Hz); 7.74 (d, 2H, Ar, *J* = 8.4 Hz); 7.85–7.90 (m, 3H, Ar); 8.06 (d, 1H, Ar, *J* = 8.4 Hz); 8.43 (exch br d, 1H, NH, *J* = 3.6 Hz). ^13^C-NMR (100 MHz, DMSO-d_6_) *δ* 6.24 (CH_2_); 23.54 (CH); 123.91 (CH); 126.31 (CH); 126.86 (CH); 128.51 (CH); 128.71 (CH); 129.00 (C); 131.61 (C); 133.00 (CH); 139.19 (C); 142.85 (C); 165.71 (C); 167.29 (C). ^77^Se-NMR (76 MHz, DMSO-d_6_) *δ* 915.30. ITMS (ESI) *m*/*z*: 359.33 [M + H]^+^; 375.92 [M + NH_4_]^+^; 716.50 [2M + H]^+^. Anal. calcd for C_17_H_14_N_2_O_2_Se (C, H, N): C, 57.15; H, 3.95; N, 7.84; found: C, 57.38; H, 3.97; N, 7.87.

#### 4-(3-Oxobenzo[*d*][1,2]selenazol-2(3*H*)-yl)-*N*-(*m*-tolyl)benzamide (3d)

Yield = 18%; oil. ^1^H-NMR (400 MHz, DMSO-d_6_) *δ* 2.30 (s, 3H, *m-CH*_3_-Ph); 6.90 (d, 1H, Ar, *J* = 8.0 Hz); 7.21 (t, 1H, Ar, *J* = 7.8 Hz); 7.48 (t, 1H, Ar, *J* = 7.2 Hz); 7.55 (d, 1H, Ar, *J* = 8.4 Hz); 7.61 (s, 1H, Ar); 7.68 (t, 1H, Ar, *J* = 7.0 Hz); 7.83 (d, 2H, Ar, *J* = 8.8 Hz); 7.91 (d, 1H, Ar, *J* = 7.6 Hz); 8.01 (d, 2H, Ar, *J* = 8.8 Hz); 8.09 (d, 1H, Ar, *J* = 8.4 Hz); 10.15 (exch br s, 1H, NH). ^13^C-NMR (100 MHz, DMSO-d_6_) *δ* 21.68 (CH_3_); 118.02 (CH); 119.23 (CH); 121.38 (CH); 123.82 (CH); 126.73 (CH); 128.91 (CH); 129.20 (CH); 129.80 (C); 129.99 (CH); 131.39 (CH); 133.50 (CH); 133.80 (C); 135.76 (CH); 135.80 (C); 138.06 (C); 139.58 (C) 141.60 (C); 143.10 (C); 164.70 (C); 167.40 (C). Anal. calcd for C_21_H_16_N_2_O_2_Se (C, H, N): C, 61.92; H, 3.96; N, 6.88; found: C, 61.67; H, 3.94; N, 6.85.

#### General procedure for compounds 4a–l, 4n and 4o

To a cooled (0 °C) suspension of benzo[*d*][1,2]selenazol-3(2*H*)-one 2^25^ (0.45 mmol) in anhydrous CH_2_Cl_2_ (5 mL), 0.90 mmol of Et_3_N and 1.12 mmol of the suitable acyl/aroyl chloride (commercially available) were added. The solution was stirred at 0 °C for 2 h and then for 2 h at room temperature. The precipitate was filtered by suction, and the organic solvent was collected and evaporated under vacuum. The residue was mixed with ice-cold H_2_O (20 mL), neutralized with 2.5 N NaOH, and the suspension was extracted with CH_2_Cl_2_ (3 × 15 mL). Evaporation of the solvent resulted in the final compounds of type 4, which were purified by flash column chromatography using petroleum ether/ethyl acetate 5 : 1 (for 4a, b, d, e, j, k, l), 10 : 1 (for 4c, f, g, n), 3 : 1 (for 4h, o), or 1 : 3 (for 4i) as eluents. Characterization of compounds 4a–c, 4e, 4h and 4o is comparable with the literature (4a,^41^4b,^28^4c,^41^4e,^41^4h,^42^4o^41^).

#### 2-Acetylbenzo[*d*][1,2]selenazol-3(2*H*)-one (4a)^41^

Yield = 35%; mp = 170–171 °C (EtOH). ^1^H-NMR (400 MHz, CDCl_3_) *δ* 2.75 (s, 3H, CH_3_); 7.40 (t, 1H, Ar, *J* = 7.4 Hz); 7.58 (d, 1H, Ar, *J* = 8.0 Hz); 7.67 (t, 1H, Ar, *J* = 7.4 Hz); 8.00 (d, 1H, Ar, *J* = 7.6 Hz). ^13^C-NMR (100 MHz, CDCl_3_) *δ* 24.98 (CH_3_); 124.23 (CH); 126.53 (CH); 128.81 (C); 129.70 (CH); 134.44 (CH); 137.92 (C); 164.72 (C); 171.60 (C). ITMS (ESI) *m*/*z*: 242.17 [M + H]^+^; 258.92 [M + NH_4_]^+^; 504.75 [2M + Na]^+^. Anal. calcd for C_9_H_7_NO_2_Se (C, H, N): C, 45.02; H, 2.94; N, 5.83; found: C, 45.20; H, 2.95; N, 5.85.

#### 2-Propionylbenzo[*d*][1,2]selenazol-3(2*H*)-one (4b)^28^

Yield = 24%; mp = 160–161 °C (EtOH). ^1^H-NMR (400 MHz, CDCl_3_) *δ* 1.26 (t, 3H, CH_2_*CH*_3_, *J* = 7.2 Hz); 3.17 (q, 2H, *CH*_2_CH_3_, *J* = 7.2 Hz); 7.38 (t, 1H, Ar, *J* = 7.6 Hz); 7.41 (d, 1H, Ar, *J* = 8.0 Hz); 7.65 (t, 1H, Ar, *J* = 7.6 Hz); 7.99 (d, 1H, Ar, *J* = 8.0 Hz). ^13^C-NMR (100 MHz, CDCl_3_) *δ* 8.61 (CH_3_); 31.35 (CH_2_); 124.30 (CH); 126.41 (CH); 129.09 (C); 129.65 (CH); 134.30 (CH); 138.09 (C); 164.58 (C); 175.50 (C). ITMS (ESI) *m*/*z*: 256.17 [M + H]^+^; 273.00 [M + NH_4_]^+^; 278.08 [M + Na]^+^. Anal. calcd for C_10_H_9_NO_2_Se (C, H, N): C, 47.26; H, 3.57; N, 5.51; found: C, 47.45; H, 3.58; N, 5.53.

#### 2-Butyrylbenzo[*d*][1,2]selenazol-3(2*H*)-one (4c)^41^

Yield = 28%; mp = 120–121 °C (EtOH). ^1^H-NMR (400 MHz, CDCl_3_) *δ* 1.02 (t, 3H, CH_2_CH_2_*CH*_3_, *J* = 7.4 Hz); 1.78 (sex, 2H, CH_2_*CH*_2_CH_3_, *J* = 7.4 Hz); 3.14 (t, 2H, *CH*_2_CH_2_CH_3_, *J* = 7.4 Hz); 7.40 (t, 1H, Ar, *J* = 7.6 Hz); 7.57 (d, 1H, Ar, *J* = 8.0 Hz); 7.65 (t, 1H, Ar, *J* = 7.6 Hz); 8.00 (d, 1H, Ar, *J* = 8.0 Hz). ^13^C-NMR (100 MHz, CDCl_3_) *δ* 13.75 (CH_3_); 17.94 (CH_2_); 39.46 (CH_2_); 124.12 (CH); 126.40 (CH); 129.16 (C); 129.67 (CH); 134.31 (CH); 138.06 (C); 164.53 (C); 174.64 (C). ITMS (ESI) *m*/*z*: 270.17 [M + H]^+^; 287.00 [M + NH_4_]^+^; 292.08 [M + Na]^+^. Anal. calcd for C_11_H_11_NO_2_Se (C, H, N): C, 49.27; H, 4.13; N, 5.22; found: C, 49.46; H, 4.15; N, 5.24.

#### 2-Isobutyrylbenzo[*d*][1,2]selenazol-3(2*H*)-one (4d)

Yield = 56%; mp = 138–139 °C (EtOH). ^1^H-NMR (400 MHz, CDCl_3_) *δ* 1.28 (d, 6H, 2*x* CH_3_, *J* = 6.8 Hz); 3.96 (quin, 1H, CH, *J* = 6.8 Hz); 7.40 (t, 1H, Ar, *J* = 7.4 Hz); 7.57 (d, 1H, Ar, *J* = 7.6 Hz); 7.65 (t, 1H, Ar, *J* = 7.6 Hz); 7.99 (d, 1H, Ar, *J* = 8.0 Hz). ^13^C-NMR (100 MHz, CDCl_3_) *δ* 18.97 (CH_3_); 35.42 (CH); 124.26 (CH); 126.55 (CH); 129.70 (CH); 129.72 (C); 134.38 (CH); 138.18 (C); 164.11 (C); 178.94 (C). ^77^Se-NMR (76 MHz, CDCl_3_) *δ* 932.95. ITMS (ESI) *m*/*z*: 270.17 [M + H]^+^; 286.92 [M + NH_4_]^+^; 292.08 [M + Na]^+^. Anal. calcd for C_11_H_11_NO_2_Se (C, H, N): C, 49.27; H, 4.13; N, 5.22; found: C, 49.46; H, 4.15; N, 5.24.

#### 2-Pivaloylbenzo[*d*][1,2]selenazol-3(2*H*)-one (4e)^41^

Yield = 22%; mp = 124–125 °C (EtOH). ^1^H-NMR (400 MHz, CDCl_3_) *δ* 1.47 (s, 9H, 3*x* CH_3_); 7.38 (t, 1H, Ar, *J* = 7.6 Hz); 7.52 (d, 1H, Ar, *J* = 8.0 Hz); 7.65 (t, 1H, Ar, *J* = 8.0 Hz); 7.97 (d, 1H, Ar, *J* = 8.0 Hz). ^13^C-NMR (100 MHz, CDCl_3_) *δ* 25.71 (CH_3_); 41.93 (C); 123.65 (CH); 126.28 (CH); 130.48 (CH); 131.35 (C); 134.04 (CH); 139.07 (C); 162.38 (C); 179.84 (C). ITMS (ESI) *m*/*z*: 284.17 [M + H]^+^; 302.21 [M + NH_4_]^+^; 328.08 [M + HCOOH]^+^; 585.67 [2M + NH_4_]^+^. Anal. calcd for C_12_H_13_NO_2_Se (C, H, N): C, 51.07; H, 4.64; N, 4.96; found: C, 51.27; H, 4.66; N, 4.98.

#### 2-pentanoylbenzo[*d*][1,2]selenazol-3(2*H*)-one (4f)

Yield = 16%; mp = 127–128 °C (EtOH). ^1^H-NMR (400 MHz, DMSO-d_6_) *δ* 1.04 (t, 3H, CH_3_, CH_2_CH_2_CH_2_*CH*_3_, *J* = 7.2 Hz); 1.49 (sex, 2H, CH_2_CH_2_*CH*_2_CH_3_, *J* = 7.2 Hz); 1.73 (quin, 2H, CH_2_*CH*_2_CH_2_CH_3_, *J* = 7.2 Hz); 3.18 (t, 2H, *CH*_2_CH_2_CH_2_CH_3_, *J* = 7.2 Hz); 7.59 (t, 1H, Ar, *J* = 7.4 Hz); 7.85 (t, 1H, Ar, *J* = 7.4 Hz); 8.02 (d, 1H, Ar, *J* = 8.0 Hz); 8.15 (d, 1H, Ar, *J* = 8.0 Hz). ^13^C-NMR (100 MHz, DMSO-d_6_) *δ* 14.08 (CH_3_); 22.22 (CH_2_); 26.54 (CH_2_); 37.32 (CH_2_); 126.51 (CH); 126.83 (CH); 128.60 (CH); 128.99 (C); 130.04 (CH); 134.59 (CH); 139.45 (C); 165.12 (C); 175.48 (C). ITMS (ESI) *m*/*z*: 284.17 [M + H]^+^; 300.92 [M + NH_4_]^+^; 306.08 [M + Na]^+^. Anal. calcd for C_12_H_13_NO_2_Se (C, H, N): C, 51.07; H, 4.64; N, 4.96; found: C, 51.27; H, 4.66; N, 4.98.

#### 2-Hexanoylbenzo[*d*][1,2]selenazol-3(2*H*)-one (4g)

Yield = 13%; mp = 143–144 °C (EtOH). ^1^H-NMR (400 MHz, DMSO-d_6_) *δ* 0.87 (t, 3H, CH_2_CH_2_CH_2_CH_2_*CH*_3_, *J* = 7.2 Hz); 1.29 (m, 4H, CH_2_CH_2_*CH*_2_*CH*_2_CH_3_); 1.60 (m, 2H, CH_2_*CH*_2_CH_2_CH_2_CH_3_); 3.02 (t, 2H, *CH*_2_CH_2_CH_2_CH_2_CH_3_, *J* = 7.2 Hz); 7.43 (t, 1H, Ar, *J* = 7.6 Hz); 7.70 (t, 1H, Ar, *J* = 7.6 Hz); 7.87 (d, 1H, Ar, *J* = 7.6 Hz); 8.00 (d, 1H, Ar, *J* = 8.0 Hz). ^13^C-NMR (100 MHz, DMSO-d_6_) *δ* 22.39 (CH_2_); 24.11 (CH_2_); 31.25 (CH_2_); 37.54 (CH_2_); 126.53 (CH); 126.83 (CH); 128.99 (CH); 130.05 (C); 134.59 (CH); 139.45 (C); 165.13 (C); 175.50 (C). ITMS (ESI) *m*/*z*: 298.17 [M + H]^+^; 315.00 [M + NH_4_]^+^; 320.08 [M + Na]^+^. Anal. calcd for C_13_H_15_NO_2_Se (C, H, N): C, 52.71; H, 5.10; N, 4.73; found: C, 52.49; H, 5.08; N, 4.71.

#### Ethyl 3-oxobenzo[*d*][1,2]selenazole-2(3*H*)-carboxylate (4h)^42^

Yield = 18%; mp = 162–163 °C (EtOH). ^1^H-NMR (400 MHz, CDCl_3_) *δ* 1.42 (t, 3H, CH_2_*CH*_3_, *J* = 7.2 Hz); 4.44 (q, 2H, *CH*_2_CH_3_, *J* = 7.2 Hz); 7.41 (t, 1H, Ar, *J* = 7.6 Hz); 7.56 (d, 1H, Ar, *J* = 8.0 Hz); 7.67 (t, 1H, Ar, *J* = 8.0 Hz); 8.05 (d, 1H, Ar, *J* = 7.6 Hz). Anal. calcd for C_10_H_9_NO_3_Se (C, H, N): C, 44.46; H, 3.36; N, 5.18; found: C, 44.28; H, 3.34; N, 5.16.

#### Ethyl 3-oxo-3-(3-oxobenzo[*d*][1,2]selenazol-2(3*H*)-yl)propanoate (4i)

Yield = 12%; mp = 158–159 °C (EtOH). ^1^H-NMR (400 MHz, CDCl_3_) *δ* 1.27 (t, 3H, CH_2_*CH*_3_, *J* = 7.2 Hz); 4.15 (s, 2H, CH_2_); 4.23 (q, 2H, *CH*_2_CH_3_, *J* = 7.2 Hz); 7.42 (t, 1H, Ar, *J* = 7.6 Hz); 7.59 (d, 1H, Ar, *J* = 8.0 Hz); 7.69 (t, 1H, Ar, *J* = 7.6 Hz); 8.01 (d, 1H, Ar, *J* = 8.0 Hz). ^13^C-NMR (100 MHz, DMSO-d_6_) *δ* 14.20 (CH_3_); 44.92 (CH_2_); 61.20 (CH_2_); 126.66 (CH); 127.04 (CH); 129.10 (CH); 129.12 (C); 134.98 (CH); 139.68 (C); 165.64 (C); 167.31 (C); 168.18 (C). ITMS (ESI) *m*/*z*: 314.25 [M + H]^+^; 330.92 [M + NH_4_]^+^. Anal. calcd for C_12_H_11_NO_4_Se (C, H, N): C, 46.17; H, 3.55; N, 4.49; found: C, 46.35; H, 3.53; N, 4.51.

#### 2-(Cyclopropanecarbonyl)benzo[*d*][1,2]selenazol-3(2*H*)-one (4j)

Yield = 30%; mp = 186–189 °C (EtOH). ^1^H-NMR (400 MHz, DMSO-d_6_) *δ* 1.06 (m, 4H, 2*x* CH_2_, *c*C_3_H_5_); 3.42 (quin, 1H, CH, *c*C_3_H_5_, *J* = 6.4 Hz); 7.43 (t, 1H, Ar, *J* = 7.4 Hz); 7.70 (t, 1H, Ar, *J* = 7.2 Hz); 7.89 (d, 1H, Ar, *J* = 8.0 Hz); 7.98 (d, 1H, Ar, *J* = 8.0 Hz). ^13^C-NMR (100 MHz, DMSO-d_6_) *δ* 11.18 (CH_2_); 14.75 (CH); 126.45 (CH); 126.86 (CH); 129.13 (CH); 130.21 (C); 134.67 (CH); 139.47 (C); 165.65 (C); 176.31 (C). ITMS (ESI) *m*/*z*: 268.17 [M + H]^+^; 290.08 [M + Na]^+^. Anal. calcd for C_11_H_9_NO_2_Se (C, H, N): C, 49.64; H, 3.41; N, 5.26; found: C, 49.44; H, 3.39; N, 5.24.

#### 2-(Cyclopentanecarbonyl)benzo[*d*][1,2]selenazol-3(2*H*)-one (4k)

Yield = 33%; mp = 141–144 °C (EtOH). ^1^H-NMR (400 MHz, CDCl_3_) *δ* 1.70–1.76 (m, 2H, CH_2_*c*C_5_H_9_); 1.85–1.93 (m, 4H, 2*x* CH_2_*c*C_5_H_9_); 2.03–2.11 (m, 2H, CH_2_, *c*C_5_H_9_); 4.10 (quin, 1H, CH *c*C_5_H_9_, *J* = 7.8 Hz); 7.39 (t, 1H, Ar, *J* = 7.6 Hz); 7.56 (d, 1H, Ar, *J* = 8.0 Hz); 7.64 (t, 1H, Ar, *J* = 7.6 Hz); 7.98 (d, 1H, Ar, *J* = 7.6 Hz). ^13^C-NMR (100 MHz, CDCl_3_) *δ* 22.59 (CH_2_); 29.91 (CH_2_); 45.52 (CH); 124.14 (CH); 126.40 (CH); 128.18 (C); 129.54 (CH); 134.24 (CH); 138.20 (C); 164.21 (C); 177.86 (C). ITMS (ESI) *m*/*z*: 296.33 [M + H]^+^; 318.17 [M + Na]^+^. Anal. calcd for C_13_H_13_NO_2_Se (C, H, N): C, 53.07; H, 4.45; N, 4.76; found: C, 53.28; H, 4.47; N, 4.78.

#### 2-(Cyclohexanecarbonyl)benzo[*d*][1,2]selenazol-3(2*H*)-one (4l)

Yield = 29%; mp = 128–131 °C (EtOH). ^1^H-NMR (400 MHz, CDCl_3_) *δ* 1.20–1.30 (m, 1H, CH–*H c*C_6_H_11_); 1.42–1.52 (m, 4H, 2*x* CH_2_*c*C_6_H_11_); 1.79 (d, 1H, CH–*H c*C_6_H_11_, *J* = 12.8 Hz); 1.81 (d, 2H, CH_2_*c*C_6_H_11_, *J* = 12.8 Hz); 2.00 (d, 2H, CH_2_*c*C_6_H_11_, *J* = 10.4 Hz); 3.68–3.75 (m, 1H, CH *c*C_6_H_11_); 7.37 (t, 1H, Ar, *J* = 7.6 Hz); 7.56 (d, 1H, Ar, *J* = 8.0 Hz); 7.62 (t, 1H, Ar, *J* = 7.6 Hz); 7.97 (d, 1H, Ar, *J* = 8.0 Hz). ^13^C-NMR (100 MHz, CDCl_3_) *δ* 25.07 (CH_2_); 25.24 (CH_2_); 25.67 (CH_2_); 28.45 (CH_2_); 29.05 (CH_2_); 44.90 (CH); 124.17 (CH); 126.41 (CH); 129.20 (C); 129.59 (CH); 134.22 (CH); 138.23 (C); 164.15 (C); 177.79 (C). ^77^Se-NMR (76 MHz, CDCl_3_) *δ* 930.91. ITMS (ESI) *m*/*z*: 310.25 [M + H]^+^; 332.08 [M + Na]^+^; 635.83 [2M + NH_4_]^+^. Anal. calcd for C_14_H_15_NO_2_Se (C, H, N): C, 54.55; H, 4.91; N, 4.54; found: C, 54.33; H, 4.89; N, 4.52.

#### 2-(3-Methylbenzoyl)benzo[*d*][1,2]selenazol-3(2*H*)-one (4n)

Yield = 38%; mp = 196–199 °C (EtOH). ^1^H-NMR (400 MHz, CDCl_3_) *δ* 2.41 (s, 3H, CH_3_, *m-CH*_3_-Ph); 7.36–7.40 (m, 3H, Ar); 7.51 (s, 2H, Ar); 7.61 (d, 1H, Ar, *J* = 8.0 Hz); 7.67 (t, 1H, Ar, *J* = 7.6 Hz); 7.95 (d, 1H, Ar, *J* = 8.0 Hz). ^13^C-NMR (100 MHz, CDCl_3_) *δ* 21.36 (CH_3_); 124.21 (CH); 125.32 (CH); 126.01 (CH); 126.63 (CH); 129.20 (C); 129.29 (CH); 129.88 (CH); 132.66 (CH); 133.35 (C); 134.52 (CH); 137.81 (C); 164.13 (C); 170.65 (C). ITMS (ESI) *m*/*z*: 318.25 [M + H]^+^; 334.75 [M + NH_4_]^+^; 656.33 [2M + Na]^+^. Anal. calcd for C_15_H_11_NO_2_Se (C, H, N): C, 56.97; H, 3.51; N, 4.43; found: C, 56.74; H, 3.49; N, 4.41.

#### 2-(4-Methoxybenzoyl)benzo[*d*][1,2]selenazol-3(2*H*)-one (4o)^41^

Yield = 12%; mp = 215–217 °C (EtOH). ^1^H-NMR (400 MHz, DMSO-d_6_) *δ* 3.83 (s, 3H, OCH_3_); 6.90–7.10 (m, 2H, Ar); 7.40–7.50 (m, 1H, Ar); 7.60–7.85 (m, 4H, Ar); 7.95–8.05 (m, 1H, Ar). ^13^C-NMR (100 MHz, DMSO-d_6_) *δ* 55.80 (CH_3_); 113.55 (2*x* CH); 126.59 (C); 126.65 (CH); 129.11 (CH); 130.00 (C); 131.96 (2*x* CH); 134.58 (CH); 139.98 (C); 162.76 (C); 165.01 (C); 170.72 (C). ITMS (ESI) *m*/*z*: 334.17 [M + H]^+^; 350.83 [M + NH_4_]^+^. Anal. calcd for C_15_H_11_NO_3_Se (C, H, N): C, 54.23; H, 3.34; N, 4.22; found: C, 54.44; H, 3.35; N, 4.24.

#### 2-Benzoylbenzo[*d*][1,2]selenazol-3(2*H*)-one (4m)^41^

To a suspension of substrate 2^25^ (0.35 mmol) in 10 ml of anhydrous THF, 0.70 mmol of sodium hydride (60% dispersion in mineral oil) and 0.42 mmol of benzoyl chloride were added. The mixture was stirred at room temperature for 2 h. After evaporation of the solvent, ice-cold H_2_O (20 mL) was added, neutralized with 2.5 N NaOH, and the suspension was extracted with CH_2_Cl_2_ (3 × 15 mL). Evaporation of the solvent resulted in the final compound, which was purified by flash column chromatography using petroleum ether/ethyl acetate 3 : 1 as the eluent and then recrystallized with ethanol. Yield = 19%; mp = 225–226 °C (EtOH). ^1^H-NMR (400 MHz, DMSO-d_6_) *δ* 7.50–8.00 (m, 8H, Ar); 8.15–8.25 (m, 1H, Ar). ^13^C-NMR (100 MHz, DMSO-d_6_) *δ* 126.66 (CH); 128.08 (CH); 128.23 (CH); 128.45 (CH); 128.98 (CH); 129.82 (C); 134.77 (CH); 135.02 (C); 139.93 (C); 164.95 (C); 171.43 (C). ITMS (ESI) *m*/*z*: 304.25 [M + H]^+^; 320.75 [M + NH_4_]^+^. Anal. calcd for C_14_H_19_NO_2_Se (C, H, N): C, 55.64; H, 3.00; N, 4.64; found: C, 55.86; H, 3.01; N, 4.66.

#### 2-(Thiophene-3-carbonyl)benzo[*d*][1,2]selenazol-3(2*H*)-one (4p)

3-Thiophen carboxylic acid (1.00 mmol) was dissolved in SOCl_2_ (1.7 mL) and refluxed for 2 h. After cooling, excess SOCl_2_ was removed under vacuum, and the residue oil was dissolved in cold anhydrous toluene (3 mL). To this solution, a mixture of intermediate 2^25^ (0.50 mmol) and Et_3_N (1.10 mmol) in 3 mL of anhydrous toluene was added. The mixture was stirred at 110 °C for 6 h, then at room temperature overnight. The precipitate was removed by vacuum filtration and, after evaporation of the solvent, an oil residue was obtained. The final compound was purified by flash column chromatography using petroleum ether/ethyl acetate 5 : 1 as the eluent. Yield = 14%; mp = 188–191 °C (EtOH). ^1^H-NMR (400 MHz, CDCl_3_) *δ* 7.31 (d, 1H, thiophene, *J* = 3.2 Hz); 7.42 (t, 2H, Ar, *J* = 7.4 Hz); 7.55–7.61 (m, 2H, 1H Ar + 1H thiophene); 7.68 (t, 1H, Ar, *J* = 7.6 Hz); 8.00 (d, 1H, Ar, *J* = 7.6 Hz); 8.37 (ds, 1H, thiophene, *J* = 2.8 Hz). ^13^C-NMR (100 MHz, CDCl_3_) *δ* 123.39 (CH); 125.97 (CH); 126.61 (CH); 128.35 (CH); 129.24 (CH); 129.64 (C); 133.82 (CH); 134.47 (CH); 136.30 (C); 138.20 (C); 167.70 (C); 169.90 (C). ITMS (ESI) *m*/*z*: 308.25 [M + H]^+^; 326.83 [M + NH_4_]^+^; 640.25 [2M + Na]^+^. Anal. calcd for C_12_H_7_NO_2_SSe (C, H, N): C, 46.76; H, 2.29; N, 4.54; found: C, 44.94; H, 2.30; N, 4.56.

#### 4-[(3-Oxobenzo[*d*][1,2]selenazol-2(3*H*)-yl)sulfonyl]phenyl pivalate (6)

Compound 6 was obtained following the same procedure used for synthesizing 4m, but using 4-(chlorosulfonyl)phenyl pivalate 5^43^ as the reagent. The mixture was stirred at room temperature for 6 h. The crude compound was purified by flash column chromatography using dichloromethane/methanol 99 : 1 as the eluent. Yield = 10%; mp = 196–197 °C (EtOH). ^1^H-NMR (400 MHz, DMSO-d_6_) *δ* 1.29 (s, 9H, 3*x* CH_3_); 7.40 (d, 2H, Ar, *J* = 8.4 Hz); 7.44 (t, 1H, Ar, *J* = 7.6 Hz); 7.72 (t, 1H, Ar, *J* = 7.6 Hz); 7.77 (d, 1H, Ar, *J* = 7.6 Hz); 8.00 (d, 1H, Ar, *J* = 8.4 Hz); 8.08 (d, 2H, Ar, *J* = 8.8 Hz). ^13^C-NMR (100 MHz, DMSO-d_6_) *δ* 27.14 (3*x* CH_3_); 122.84 (C); 123.74 (CH); 126.83 (CH); 128.05 (C); 128.90 (CH); 130.34 (3*x* CH); 134.55 (CH); 135.81 (C); 140.89 (C); 155.31 (C); 165.27 (C); 176.28 (C). ITMS (ESI) *m*/*z*: 456.83 [M + NH_4_]^+^. Anal. calcd for C_18_H_17_NO_5_SSe (C, H, N): C, 49.32; H, 3.91; N, 3.20; found: C, 49.51; H, 3.92; N, 3.21.

#### Phenyl 3-oxobenzo[*d*][1,2]selenazole-2(3*H*)-carboxylate (7)

To a cooled (0 °C) solution of benzo[*d*][1,2]selenazol-3(2*H*)-one 2^25^ (0.55 mmol) in anhydrous acetonitrile (10 mL), 0.82 mmol of DIPEA and 0.82 mmol of phenyl chloroformate were added. The mixture was stirred for 3 h at room temperature. After evaporation of the solvent, ice-cold H_2_O (20 mL) was added, and the pH was adjusted to 2–3 with HCl 6 M. The suspension was extracted with CH_2_Cl_2_ (3 × 15 mL), the organic solvent was recovered and dehydrated with sodium sulfate. Evaporation of the solvent resulted in the final compound, which was purified by flash column chromatography using petroleum ether/ethyl acetate 3 : 1 as the eluent. Yield = 22%; mp = 170–171 °C (EtOH). ^1^H-NMR (400 MHz, DMSO-d_6_) *δ* 7.29–7.34 (m, 3H, Ar); 7.45–7.50 (m, 3H, Ar); 7.75 (t, 1H, Ar, *J* = 7.8 Hz); 7.92 (d, 1H, Ar, *J* = 7.2 Hz); 8.04 (d, 1H, Ar, *J* = 8.0 Hz). Anal. calcd for C_14_H_9_NO_3_Se (C, H, N): C, 52.85; H, 2.85; N, 4.40; found: C, 52.64; H, 2.84; N, 4.38.

#### *N*-Cyclopropyl-3-oxobenzo[*d*][1,2]selenazole-2(3*H*)-carboxamide (8)

To a cooled (0 °C) solution of intermediate 7 (0.31 mmol) in anhydrous CH_2_Cl_2_ (10 mL), a catalytic amount of DIPEA and 0.46 mmol of cyclopropylamine were added. The mixture was stirred at reflux for 2 h under nitrogen. After evaporation of the solvent, ice-cold H_2_O (20 mL) was added, and the pH was adjusted to 2–3 with HCl 6 M. The suspension was extracted with CH_2_Cl_2_ (3 × 15 mL), the organic solvent was recovered and dehydrated with sodium sulfate. Evaporation of the solvent resulted in the final compound, which was purified by flash column chromatography using dichloromethane/methanol 99 : 1 as the eluent. Yield = 46%; mp = 175–176 °C (EtOH). ^1^H-NMR (400 MHz, DMSO-d_6_) *δ* 0.59–0.63 (m, 2H, CH_2_*c*C_3_H_5_); 0.71–0.76 (m, 2H, CH_2_*c*C_3_H_5_); 2.72–2.78 (m, 1H, CH *c*C_3_H_5_); 7.46 (t, 1H, Ar, *J* = 7.6 Hz); 7.71 (t, 1H, Ar, *J* = 7.6 Hz); 7.87 (d, 1H, Ar, *J* = 7.6 Hz); 8.05 (d, 1H, Ar, *J* = 8.0 Hz); 8.84 (exch br s, 1H, NH). ^13^C-NMR (100 MHz, DMSO-d_6_) *δ* 6.27 (CH_2_); 6.85 (CH_2_); 23.38 (CH); 126.49 (CH); 126.77 (CH); 128.61 (CH); 129.52 (C); 134.07 (CH); 139.72 (C); 154.14 (C); 166.47 (C). ^77^Se-NMR (76 MHz, DMSO-d_6_) *δ* 894.60. ITMS (ESI) *m*/*z*: 283.17 [M + H]^+^; 305.08 [M + Na]^+^. Anal. calcd for C_11_H_10_N_2_O_2_Se (C, H, N): C, 46.99; H, 3.58; N, 9.96; found: C, 46.80; H, 3.56; N, 9.92.

### Single crystal X-ray diffraction

Single crystal X-ray diffraction data of compound 4d were collected on a Bruker Apex-II diffractometer equipped with a CCD detector (*T* = 100 K, Cu-Kα radiation (*λ* = 1.54178 Å). The data were collected using APEX2 software,^44^ and data integration and reduction were performed with the Bruker SAINT software.^45^ The crystal structures were solved using the SIR-2004 package^46^ and refined by full-matrix least squares against *F*^2^ using all data (SHELXL-2018/3).^47^ All non-hydrogen atoms were refined with anisotropic displacement parameters, while all hydrogen atoms were found in the Fourier density maps. Their coordinates were freely refined, while their thermal parameter was set in accordance with that of the atoms to which they are bonded. Geometric calculations were performed by PARST97,^48^ and molecular plots were produced using CCDC Mercury (v. 2022.3.0).^49^

### HNE inhibition assay

All compounds were dissolved in 100% DMSO at 5 mM stock concentrations and then subsequently diluted in 100% DMSO at various concentrations appropriate for use in the dose–response assays. The final concentration of DMSO in the reactions was 1%, and this level of DMSO had no effect on enzyme activity. The HNE inhibition assay was performed in black flat-bottom 96-well microtiter plates. Briefly, a buffer solution containing 200 mM Tris-HCl, pH 7.5, 0.01% bovine serum albumin, and 0.05% Tween-20 and 20 mU mL^−1^ of HNE (Calbiochem) was added to wells containing different concentrations of each compound. The reaction was initiated by addition of 25 μM elastase substrate (*N*-methylsuccinyl-Ala-Ala-Pro-Val-7-amino-4-methylcoumarin, Calbiochem) in a final reaction volume of 100 μL per well. Kinetic measurements were obtained every 30 s for 10 min at 25 °C using a Fluoroskan Ascent FL fluorescence microplate reader (Thermo Electron, MA) with excitation and emission wavelengths of 355 and 460 nm, respectively. For all compounds tested, the concentration of inhibitor that caused 50% inhibition of the enzymatic reaction (IC_50_) was calculated by plotting % inhibition *versus* logarithm of inhibitor concentration (at least six points).^50^ The data are presented as the mean values of at least three independent experiments with relative standard deviations of <15%.

### Isolation of murine bone marrow leukocytes

All animal use was conducted in accordance with a protocol approved by the Institutional Animal Care and Use Committee (IACUC) at Montana State University (protocol approval number 2022-45-IA). Bone marrow leukocytes were flushed from tibias and femurs of BALB/c mice (2-month old; 25 g body weight) or CD-1 female mice (2.5-month old; 30 g body weight) with HBSS, filtered through a 70 μm nylon cell strainer (BD Biosciences, Franklin Lakes, NJ) to remove cell clumps and bone particles.

### Antiradical activity assay

To evaluate antiradical activity, murine bone marrow leukocytes from BALB/c mice were resuspended at 10^6^ cells per ml in HBSS containing Ca^2+^ and Mg^2+^ (HBSS^+^) and supplemented with 40 μM L-012 (Wako Chemicals, Richmond, VA),^51^ since L-012-enhanced chemiluminescence represents a sensitive and reliable method for detecting ROS.^52^ The cells (100 μl) were then aliquoted into wells of 96-well flat-bottom white microtiter plates containing test compounds diluted in 100 μl of HBSS^+^ (final DMSO concentration of 1%). The cells were activated by adding 200 nM PMA (Sigma Chemical Co., St. Louis, MO), and luminescence was monitored for 60 min (2 min intervals) at 37 °C using a Fluoroskan Ascent FL microtiter plate reader (Thermo Electron, Waltham, MA). The compound concentration that inhibited ROS production by 50% of the PMA-stimulated response was determined by graphing the percentage inhibition of the 60 min integrated luminescence *versus* the logarithm of concentration of test compound. Each curve was determined using five to seven compound concentrations. Curve fitting and calculation of median effective concentration values (IC_50_) were performed by nonlinear regression analysis of the dose–response curves generated using Prism 10 (GraphPad Software, Inc., San Diego, CA, USA).

### Cytotoxicity assay

The cytotoxicity of compounds for murine bone marrow leukocytes was analyzed using a CellTiter-Glo Luminescent Cell Viability Assay kit (Promega), according to the manufacturer's protocol. Briefly, bone marrow leukocytes from CD-1 mice were incubated at a density of 10^4^ cells per well with different concentrations of compounds (the final concentration of DMSO was 1%) for 90 min at 37 °C and 5% CO_2_. Following treatment, the substrate was added to the cells, and the samples were analyzed with a FlexStation 3 microplate reader (Molecular Devices, San Jose, CA), USA.

## Conclusions

In conclusion, we report the first series of selenium-containing compounds with a dual HNE inhibitory and antioxidant activity. In confirmation of our previously published results, HNE inhibitory activity appears to be due to the attack of the Ser195 OH on the N–CO group at position 2. Indeed, we also found a correlation between HNE inhibitory activity and the size of the alkyl group linked to nitrogen in the benzo[*d*][1,2]selenazol-3(2*H*)-one scaffold. Furthermore, antiradical activity was due to the presence of the selenium atom, implying an ebselen-like mechanism that will need to be confirmed. The best dual compounds exhibiting a good balanced between anti-HNE and antiradical activity were compounds 4d, 4f, and 4j, which have an isopropyl, *n*-butyl, and cyclopropyl group, respectively, linked to position 2 of the N–CO function. These initial results are encouraging since this dual therapeutic approach/effect could be useful for the treatment of respiratory diseases involving both inflammation and oxidative stress. Further development/improvement of these compounds is warranted, including the synthesis of additional compounds and confirmation of the mechanism of action, complemented by modelling studies to understand the possible involvement of selenium in the active site.

## Ethical statement

All animal procedures were performed in accordance with the Guidelines for Care and Use of Laboratory Animals of Montana State University and approved by the Animal Ethics Committee of Montana State University.

## Author contributions

GG, GB, MP: methodology, software, data curation. IAS: investigation, validation. CV, FC, PR, ML: investigation, validation. PP: data curation, conceptualization, review & editing. LC: conceptualization, writing – original draft, review & editing. MTQ: conceptualization, review & editing. MPG: supervision, review & editing, project administration. All of the authors have given approval to the final version of the manuscript.

## Conflicts of interest

The authors declare that they have no known competing financial interests or personal relationships that could have appeared to influence the work reported in this paper. There are no conflicts to declare.

## Supplementary Material

MD-015-D3MD00736G-s001

MD-015-D3MD00736G-s002
