# Supplementary material for: Ebselen analogues with dual human neutrophil elastase (HNE) inhibitory and antiradical activity
Source: RSC Med Chem. 2024 Mar 19;15(4):1247–57. doi: 10.1039/d3md00736g (PMC11042244; doi:10.1039/d3md00736g)
Supplement: MD-015-D3MD00736G-s001 [file MD-015-D3MD00736G-s001.pdf]

Electronic Supplementary Material (ESI) for RSC Medicinal Chemistry.
This journal is © The Royal Society of Chemistry 2024

Supporting Information

for

# Ebselen analogues with dual human neutrophil elastase (HNE) inhibitory and antiradical activity

Letizia Crocetti*[a], Francesca Catarzi[a], Maria Paola Giovannoni[a], Claudia Vergelli[a], Gianluca Bartolucci[a], Marco Pallecchi[a], Paola Paoli[b], Patrizia Rossi[b], Martina Lippi[b], Igor A. Schepetkin[c], Mark T. Quinn[c], and Gabriella Guerrini[a]

[a]*NEUROFARBA, Pharmaceutical and Nutraceutical Section, University of Florence, Via Ugo Schiff 6, 50019 Sesto Fiorentino, Italy*

[b]*Department of Industrial Engineering, Via Santa Marta 3 - 50136 Florence University of Florence, Florence, Italy*

[c]*Department of Microbiology and Cell Biology, Montana State University, Bozeman, MT 59717, USA*

***Corresponding Author**
Dr. Letizia Crocetti
Department of NEUROFARBA
Via Ugo Schiff 6
Sesto Fiorentino 50019 Firenze
Tel +39-055-4573683
E-mail: letizia.crocetti@unifi.it

**Table of contents**

1. $^{1}$H NMR, $^{13}$C NMR, $^{77}$Se NMR spectra of some representative final compounds (pages 1-18)
2. Mass Spectrometry spectra (pages 19-36) and purity data (**Table S1**)
3. Crystallographic data and refinement parameters of **4d** (**Table S2, Figure S1**)
4. Volume and surface accessible to solvent for **4d**, **4e**, **4j**, **4g** and **4k** and for the *i*-propyl, *t*-butyl, *c*-propyl, *n*-pentyl and *c*-pentyl residues (**Table S3**)
5. Overlapping of surface accessible to solvent of compounds **4d**, **4e**, **4j**, (**Figure S2, Figure S3**); **4g** and **4k** (**Figure S4, Figure S5**)

1. *$^{1}$H-NMR, $^{13}$C-NMR, $^{77}$Se-NMR spectra of some representative final compounds*

1H NMR Compound 3c
DMSO

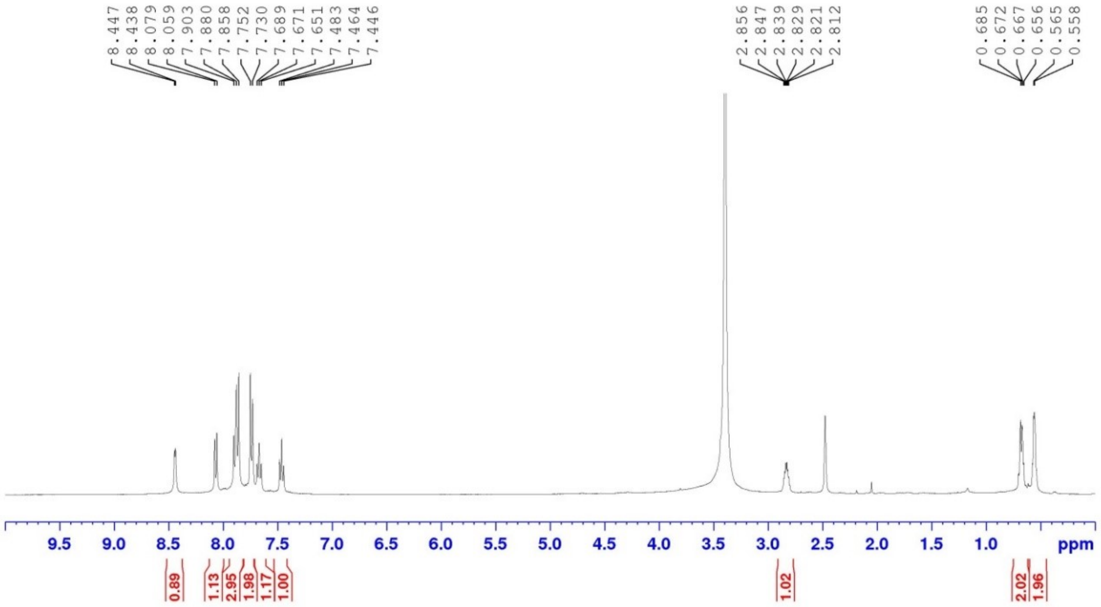


1H NMR Compound 3c
DMSO + D2O

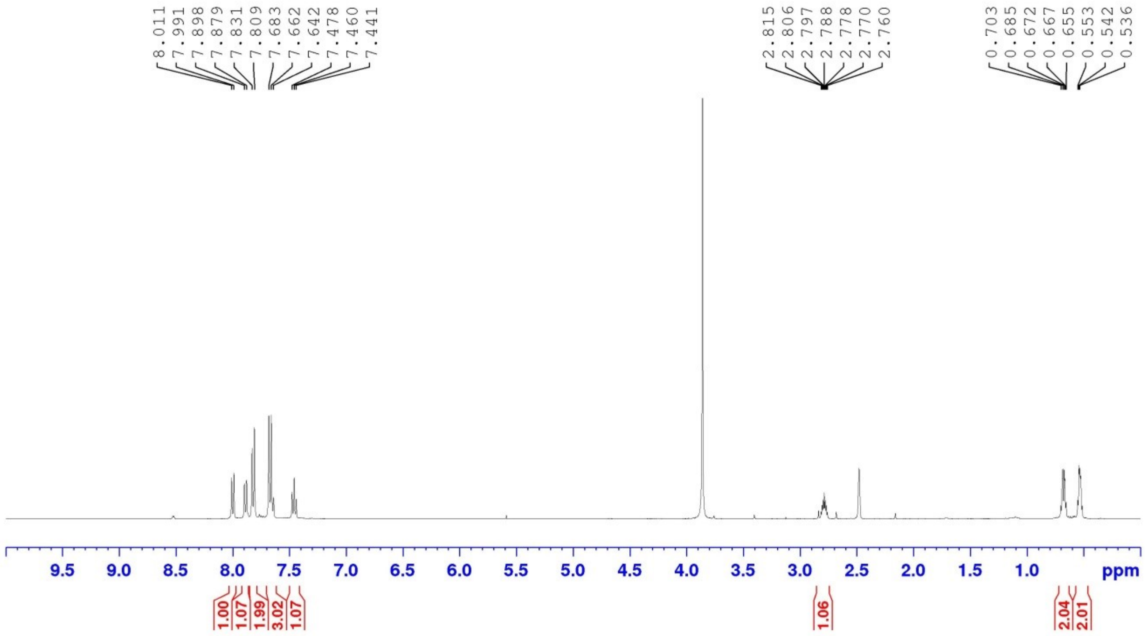

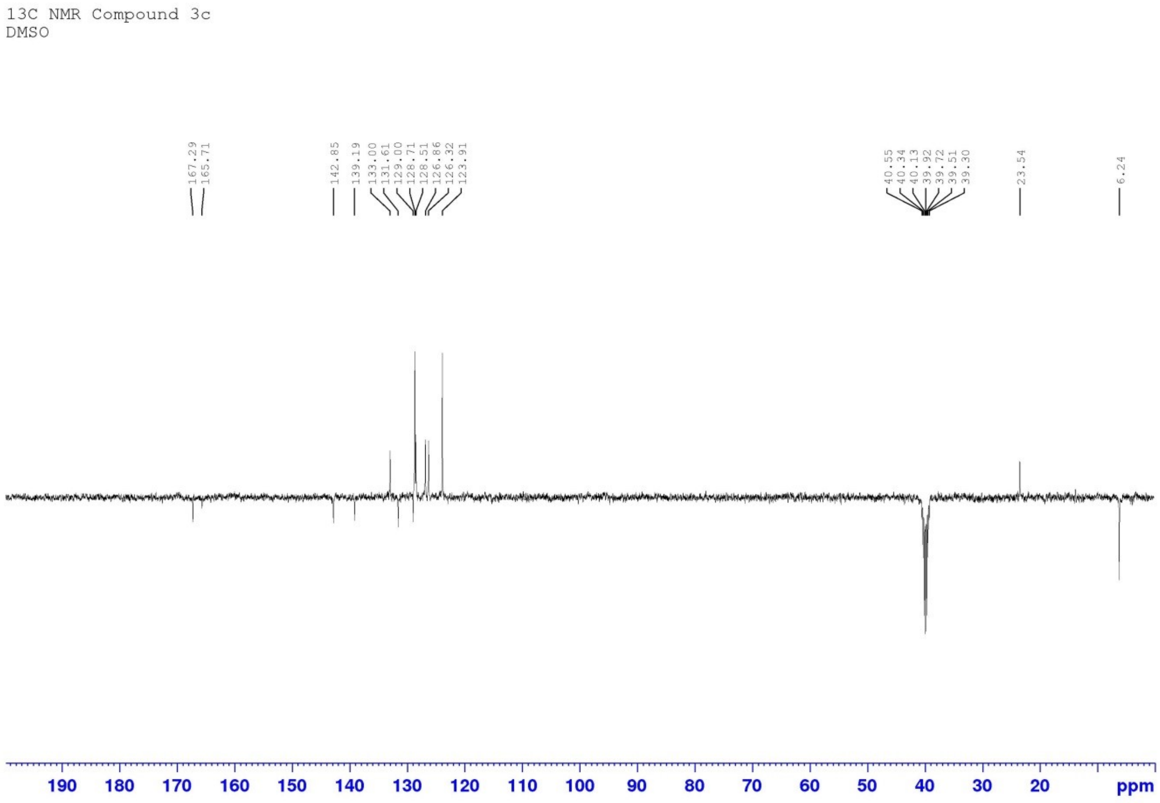
13C NMR Compound 3c
DMSO
167.29
165.71
142.85
139.19
133.00
131.61
129.00
128.71
128.51
126.86
126.32
123.91
40.55
40.34
40.13
39.92
39.72
39.51
39.30
23.54
6.24
190
180
170
160
150
140
130
120
110
100
90
80
70
60
50
40
30
20
ppm

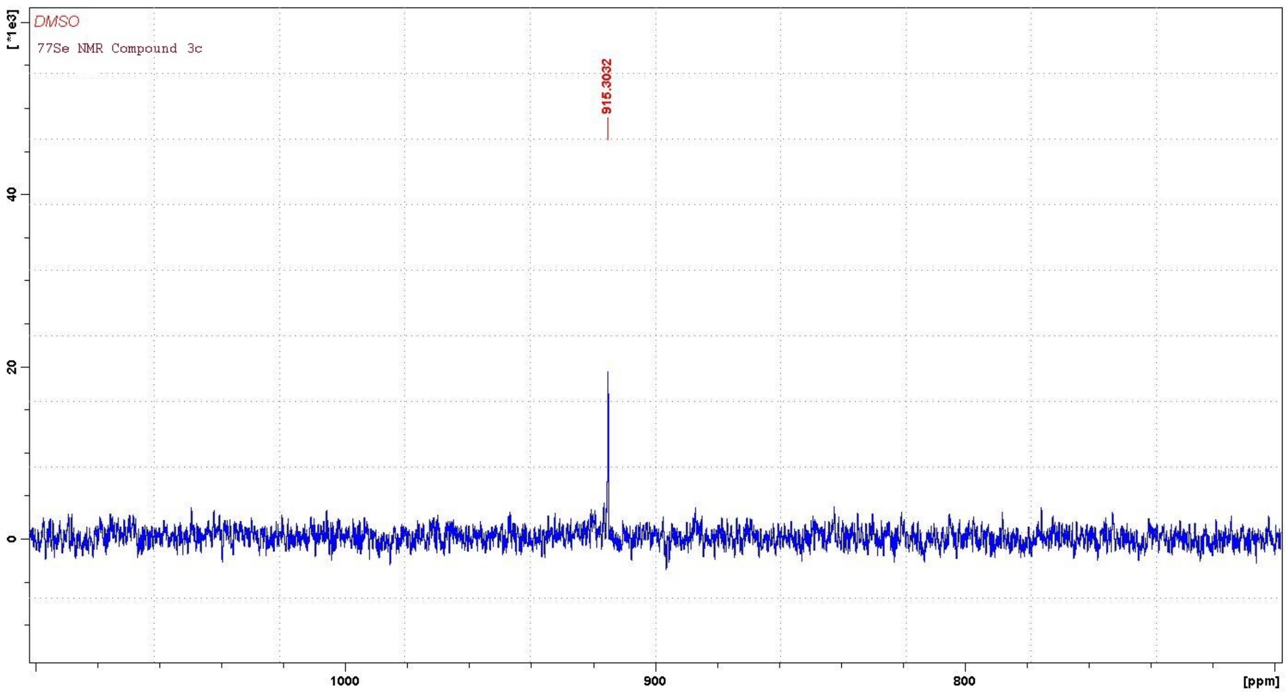
DMSO
77Se NMR Compound 3c
915.3032
[*1e3]
40
20
0
1000
900
800
[ppm]

1H NMR Compound 4a
CDCl3

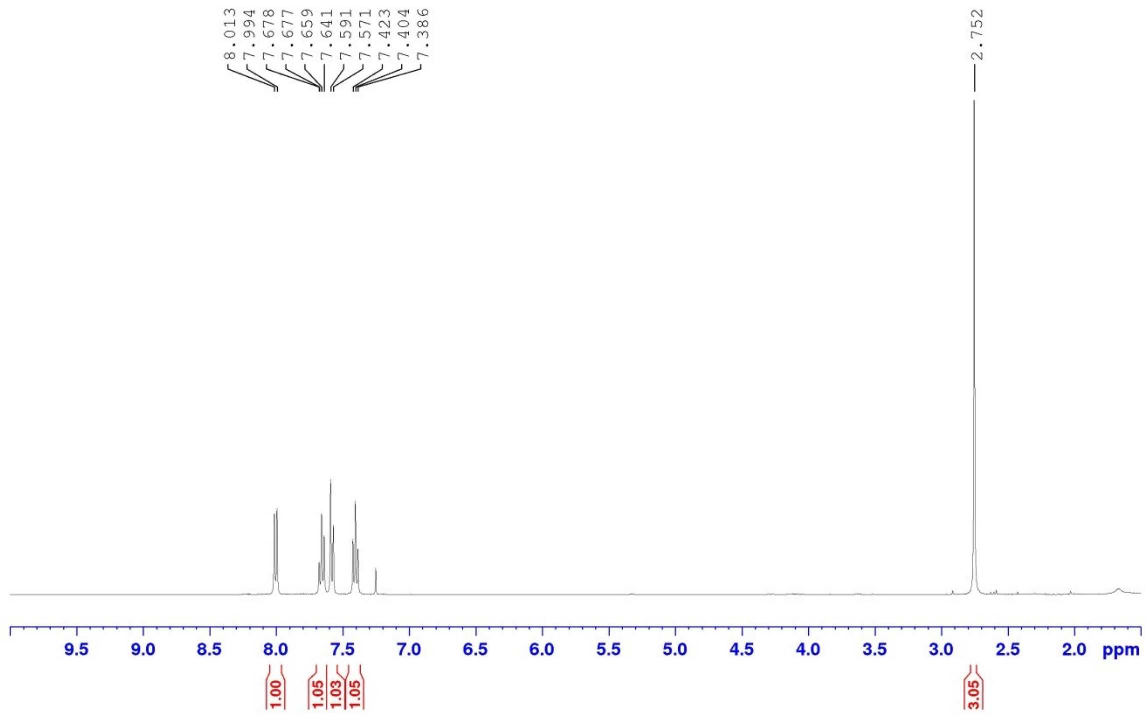


13C NMR Compound 4a
CDCl3

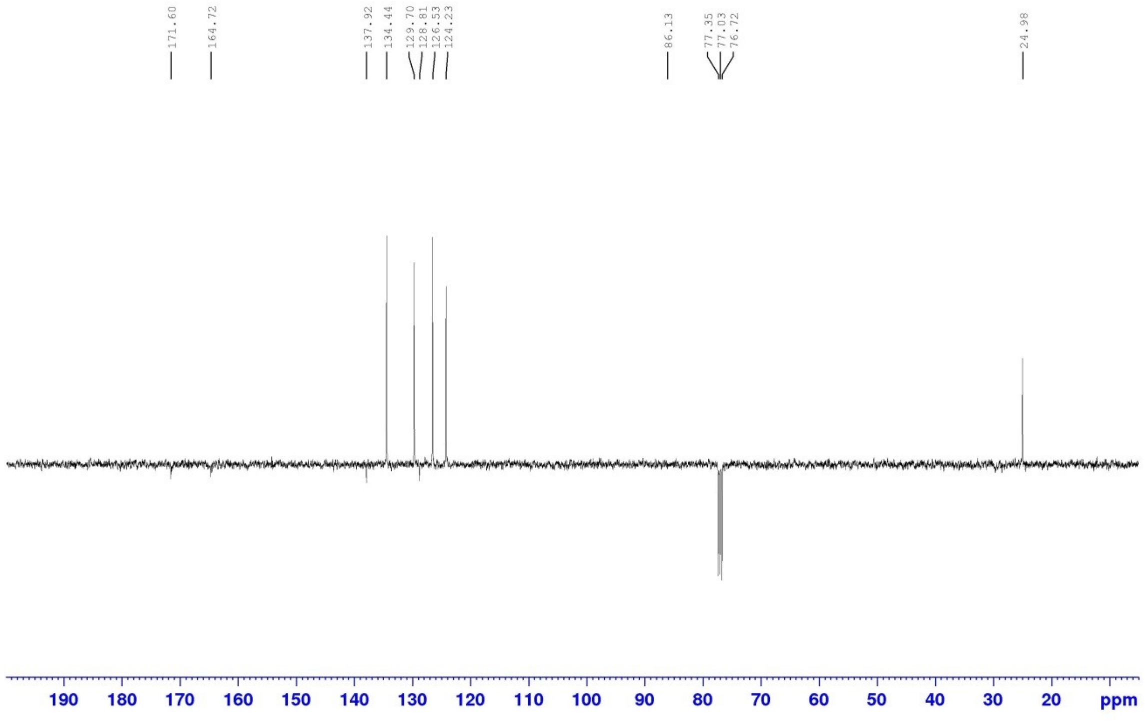

1H NMR Compound 4b
CDCl3

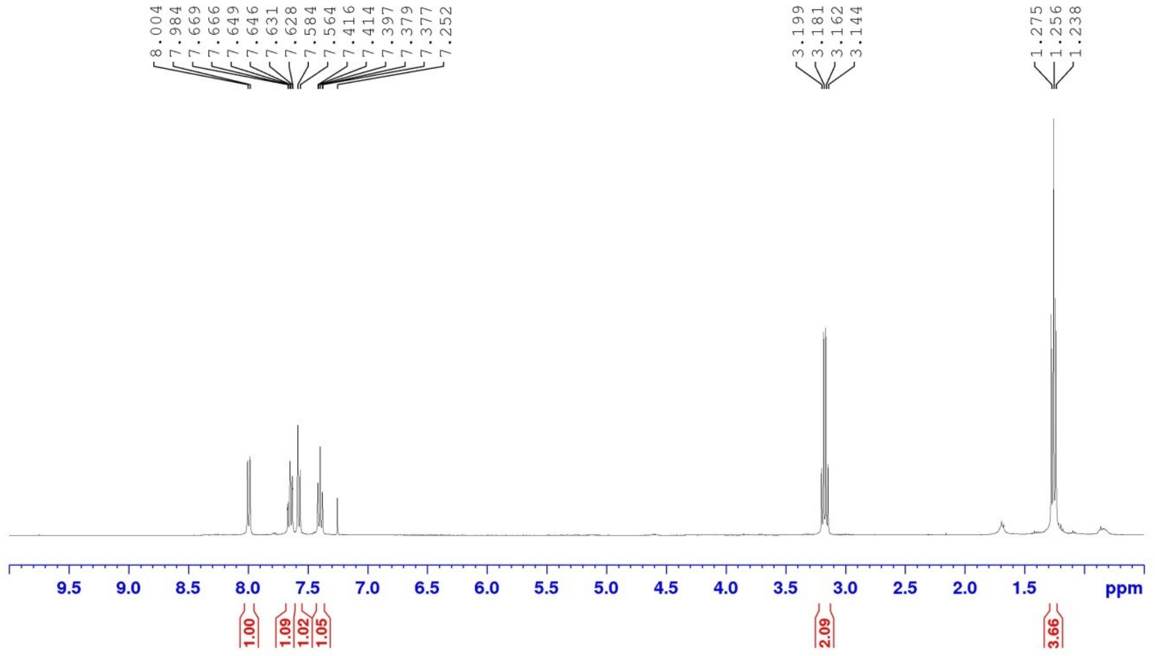


13C NMR Compound 4b
CDCl3

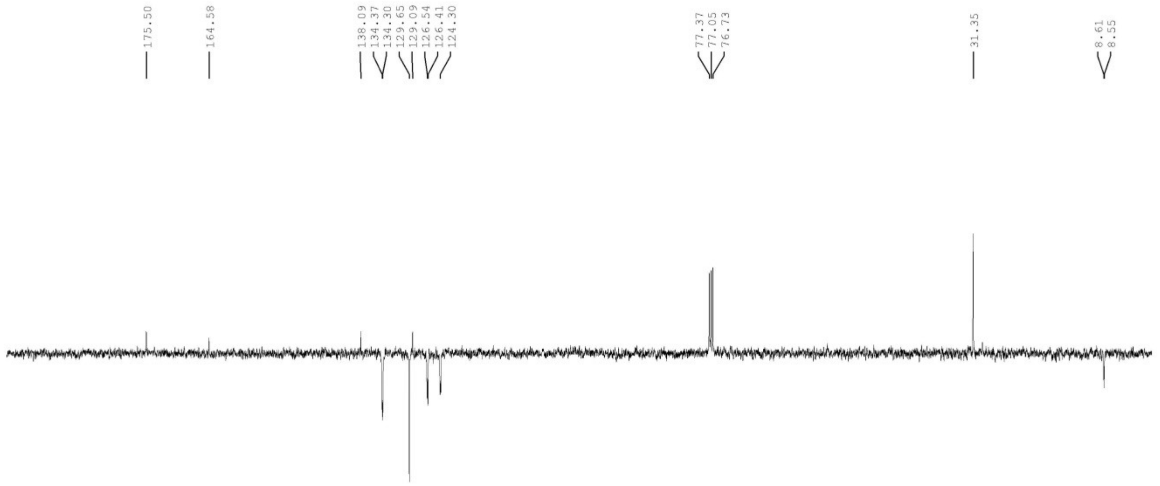

1H NMR Compound 4c
CDCl3

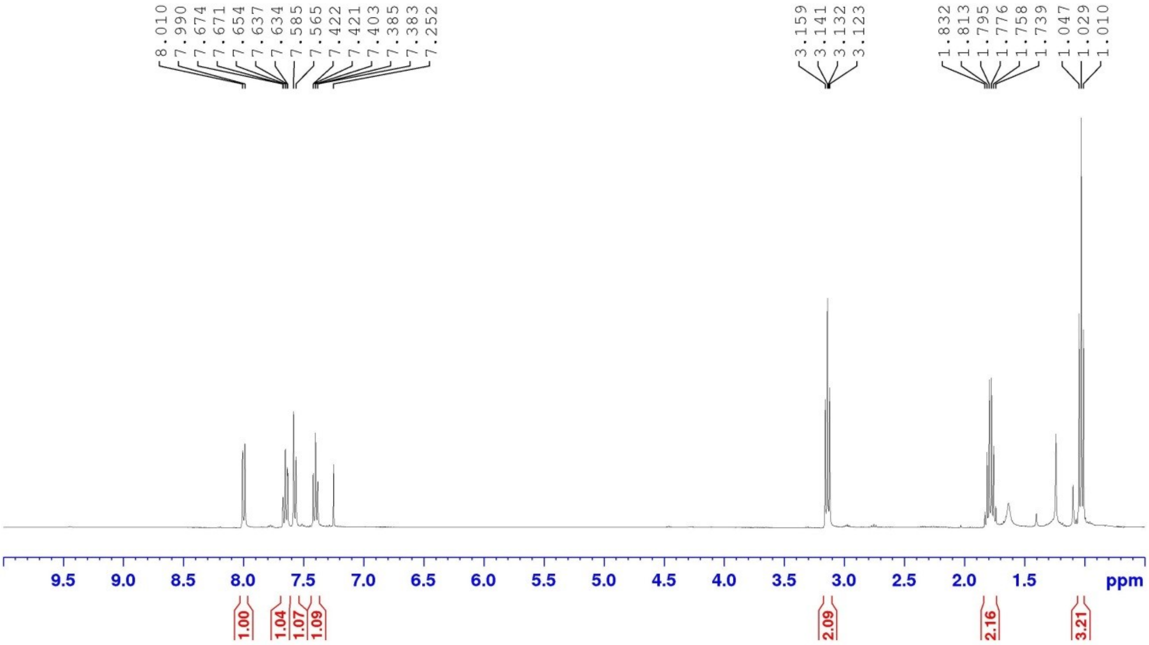


13C NMR Compound 4c
CDCl3

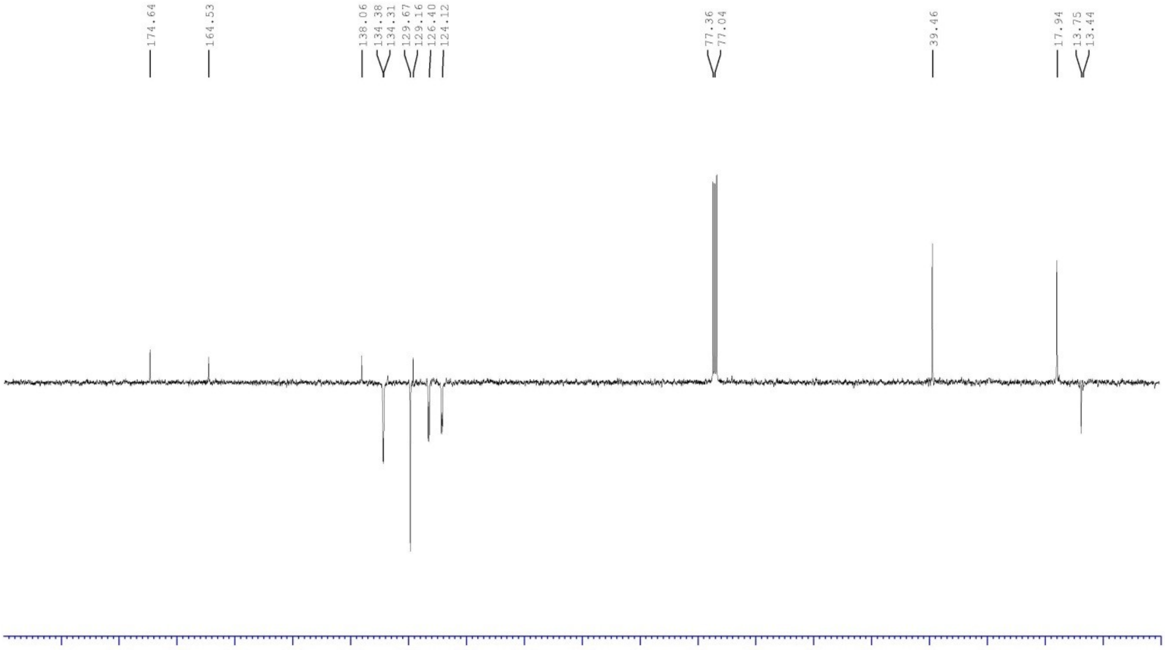

1H NMR Compound 4d
CDCl3

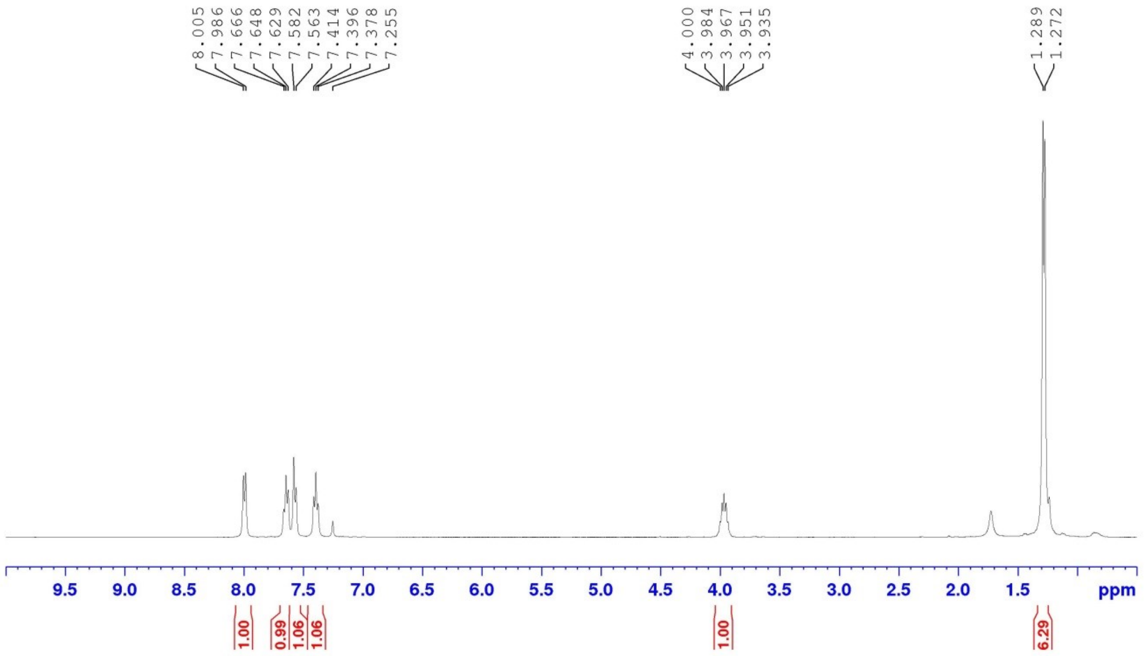


13C NMR Compound 4d
CDCl3

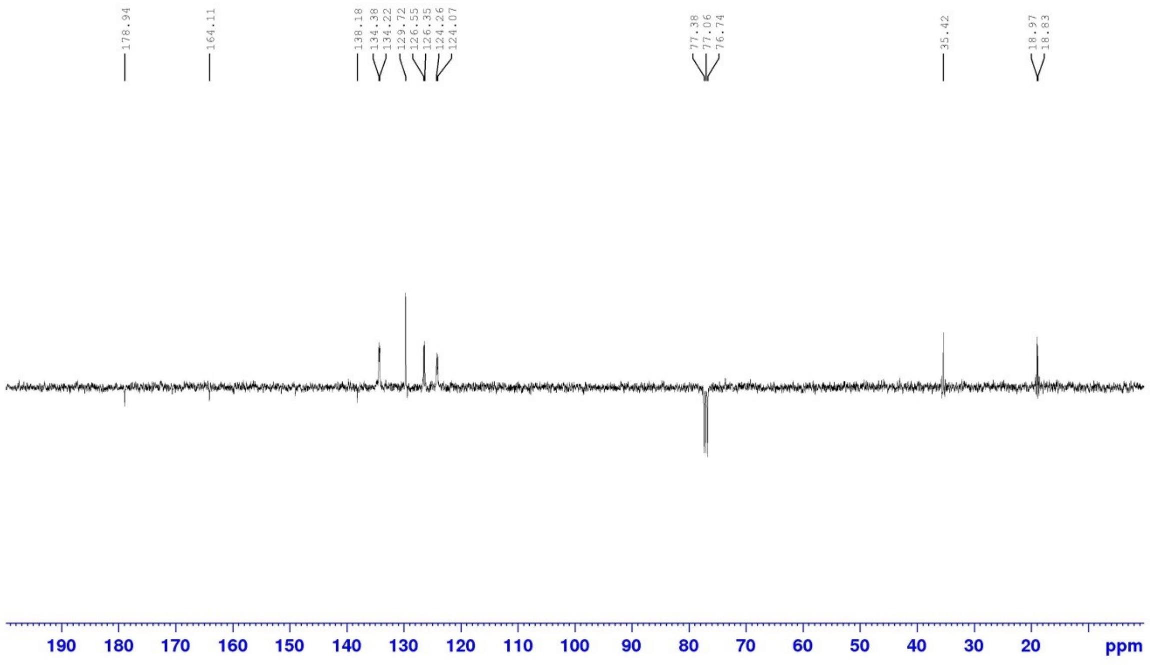

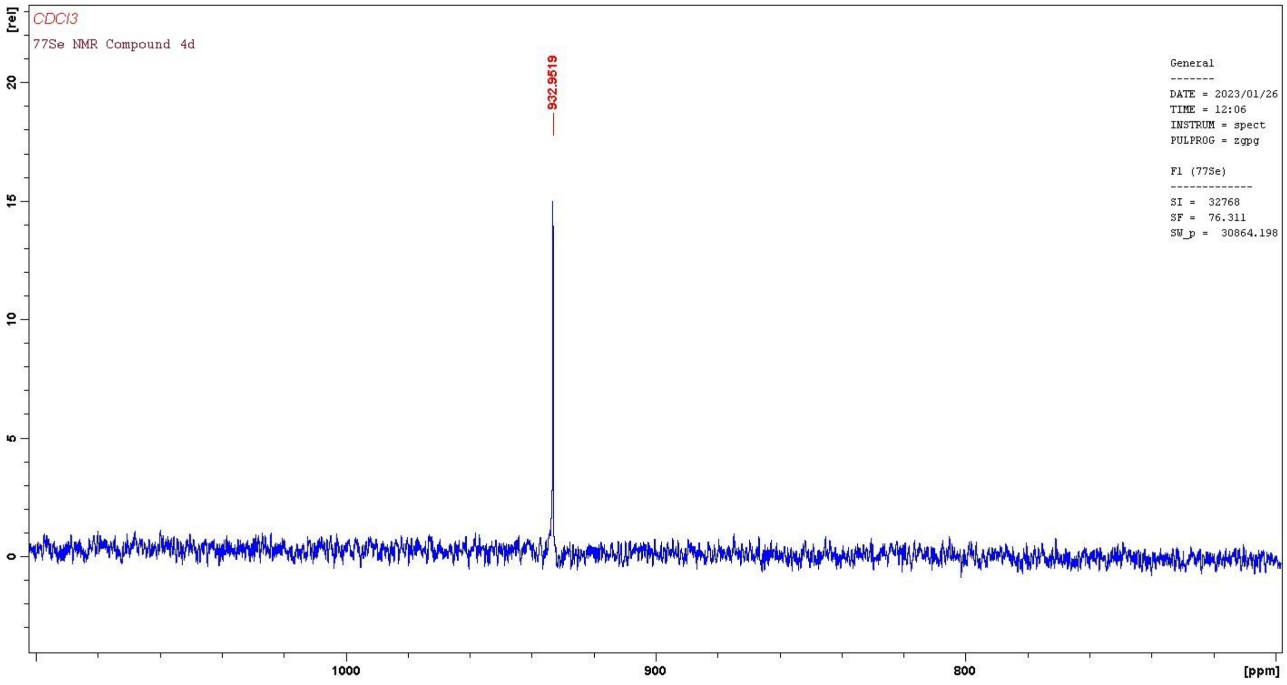
CDCl3
77Se NMR Compound 4d
932.9519
General
-------
DATE = 2023/01/26
TIME = 12:06
INSTRUM = spect
PULPROG = zgpg
F1 (77Se)
-------------
SI = 32768
SF = 76.311
SW_p = 30864.198
[rel]
20
15
10
5
0
1000
900
800
[ppm]

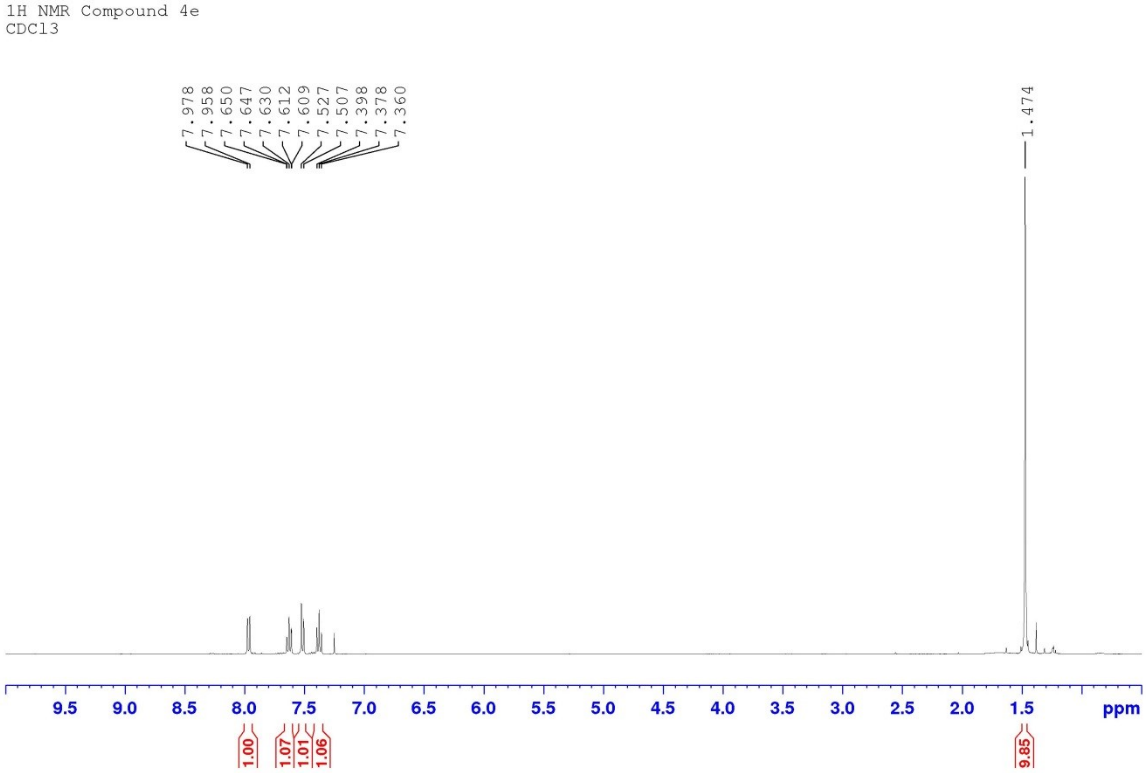
1H NMR Compound 4e
CDCl3
7.978
7.958
7.650
7.647
7.630
7.612
7.609
7.527
7.507
7.398
7.378
7.360
1.474
9.5
9.0
8.5
8.0
7.5
7.0
6.5
6.0
5.5
5.0
4.5
4.0
3.5
3.0
2.5
2.0
1.5
ppm
1.00
1.07
1.01
1.06
9.85

13C NMR Compound 4e
CDCl3

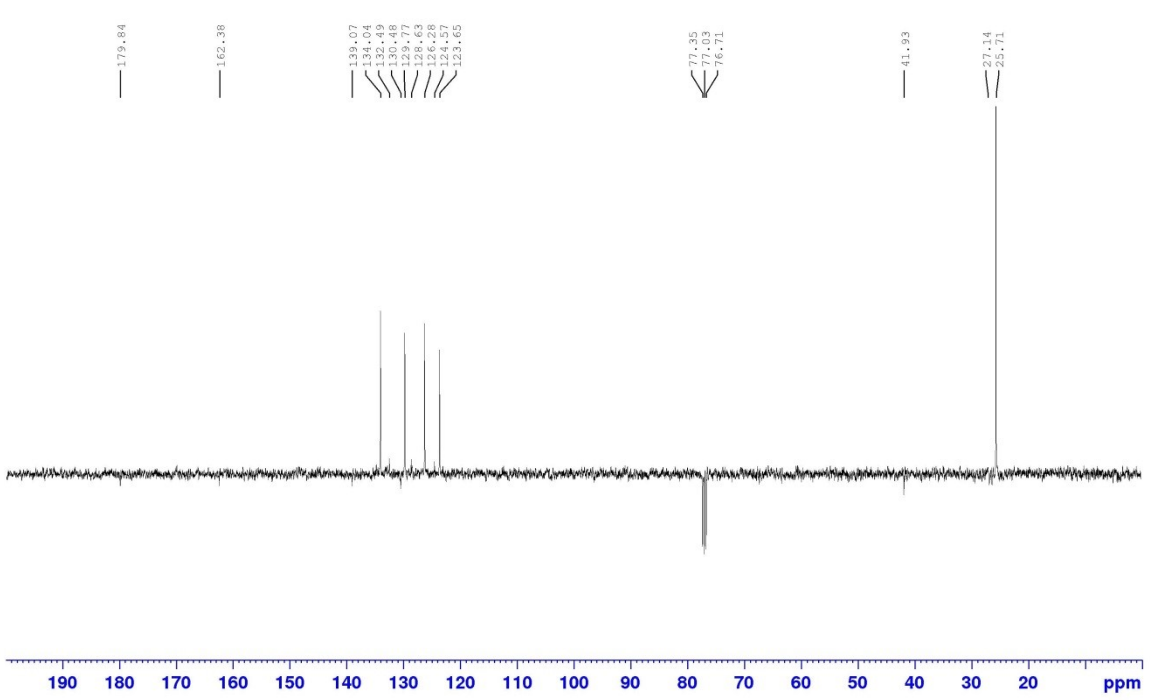


1H NMR Compound 4f
DMSO

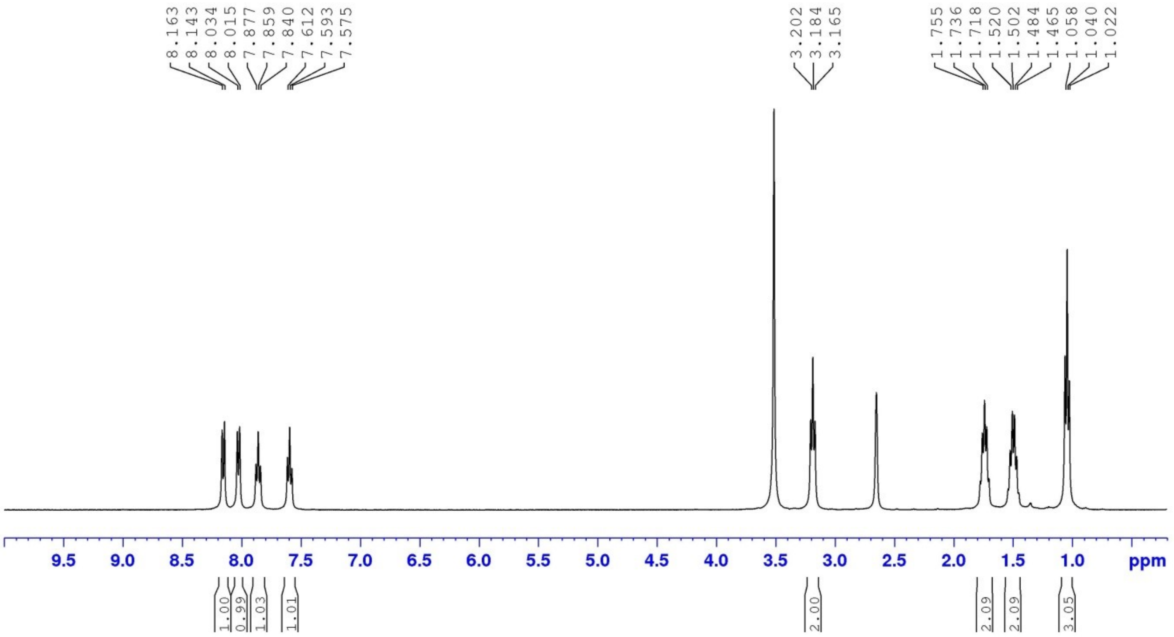

13C NMR Compound 4f
DMSO

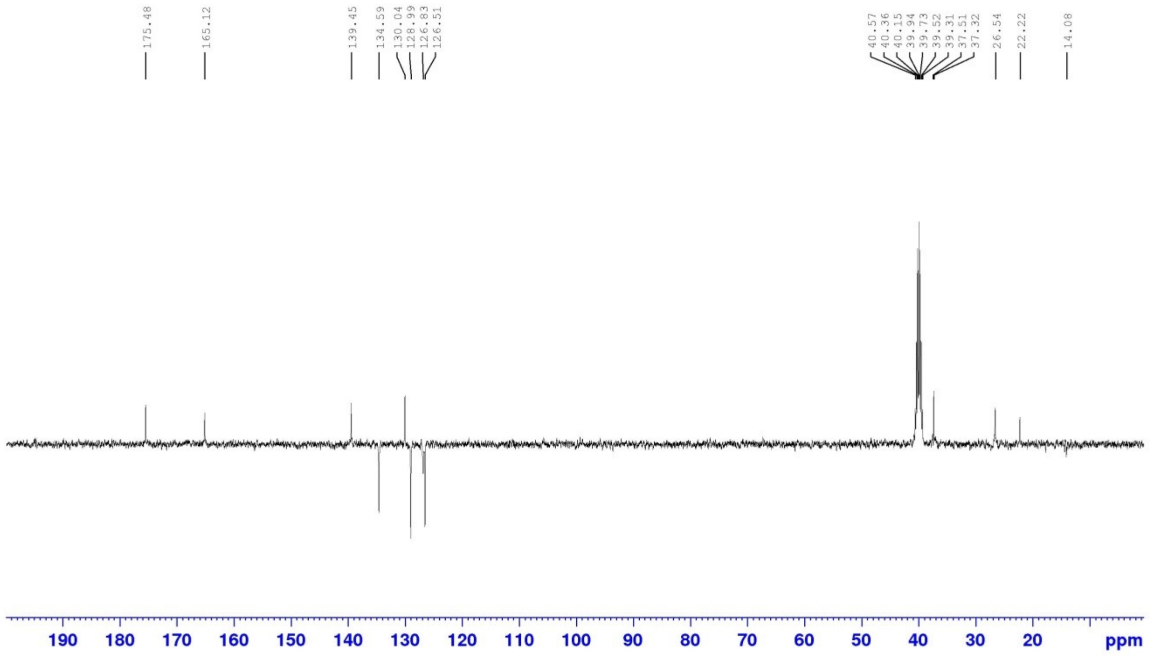


1H NMR Compound 4g
DMSO

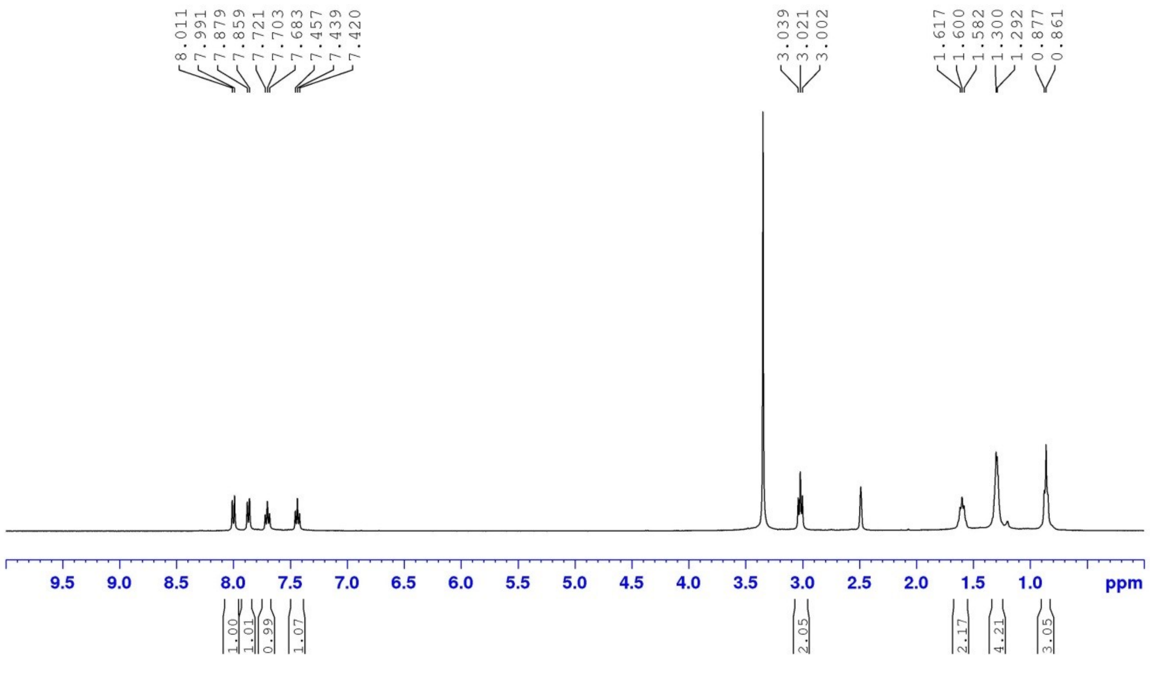

13C NMR Compound 4g
DMSO

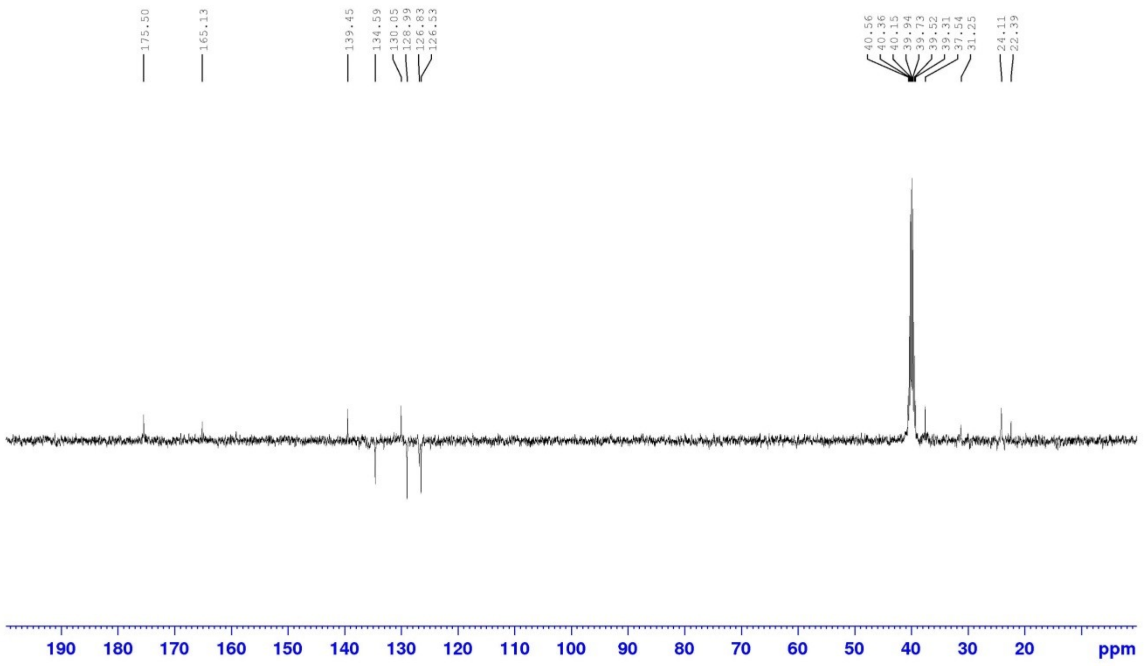


1H NMR Compound 4j
DMSO

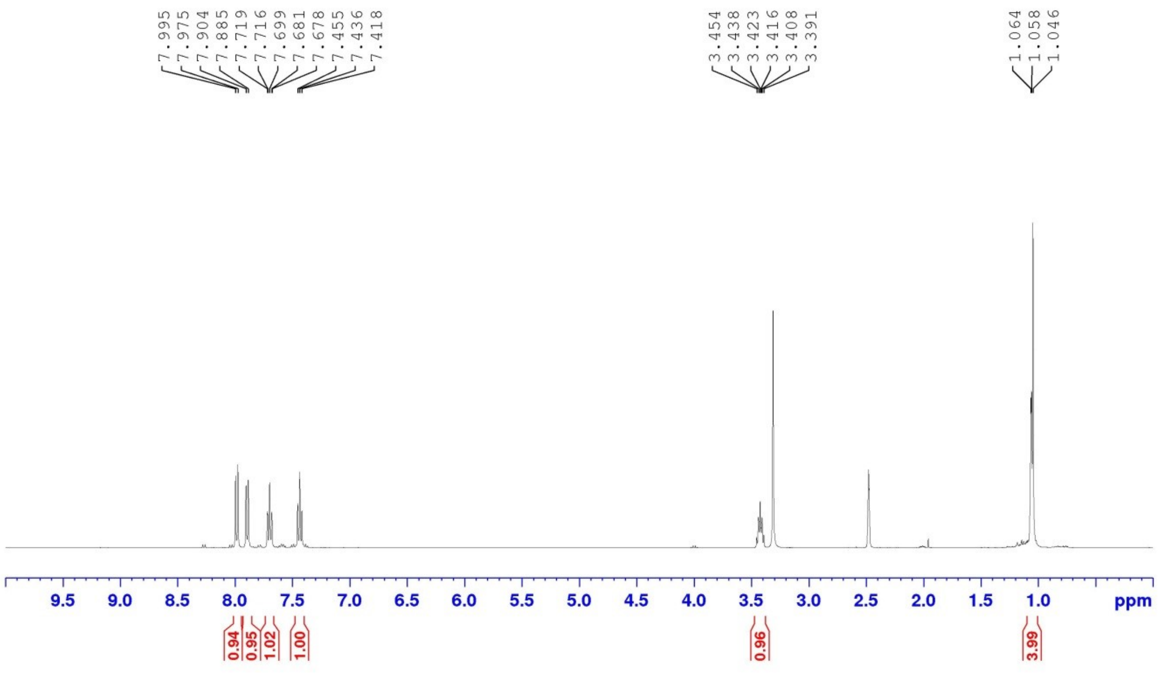

13C NMR Compound 4j
DMSO

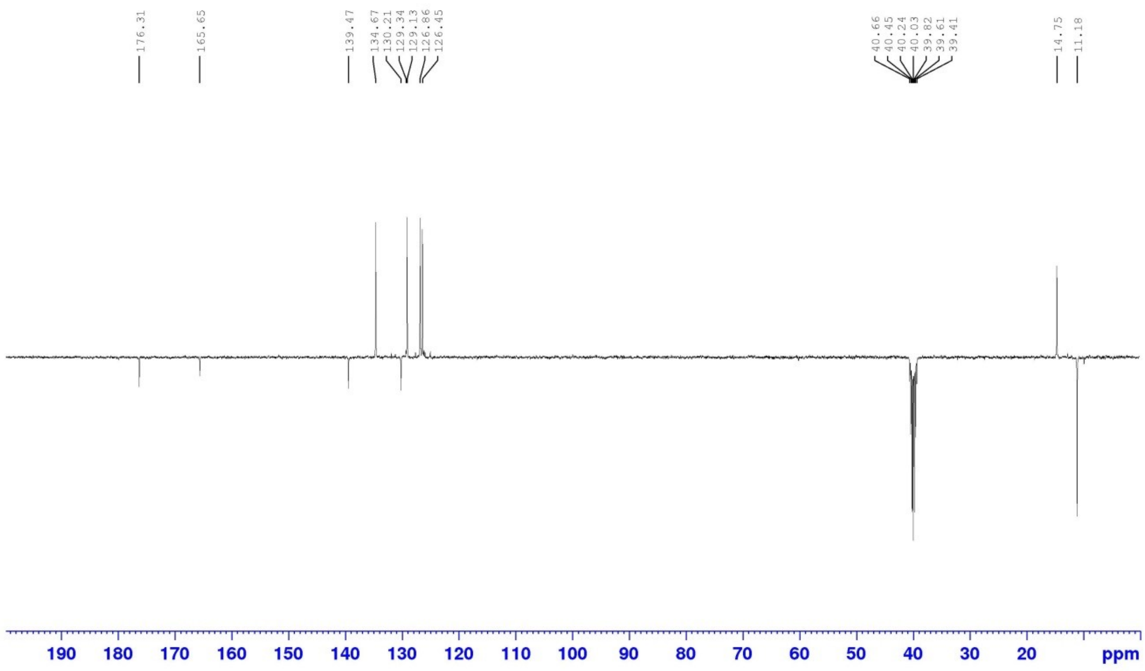


1H NMR Compound 4k
CDCl3

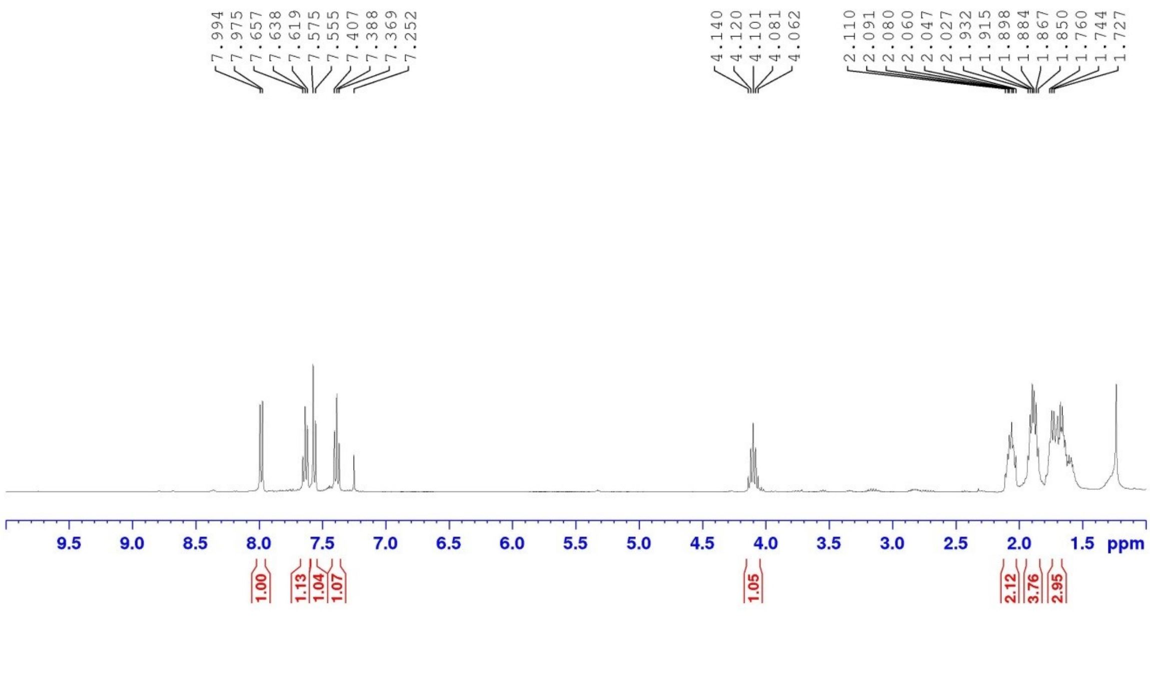

13C NMR Compound 4k
CDCl3

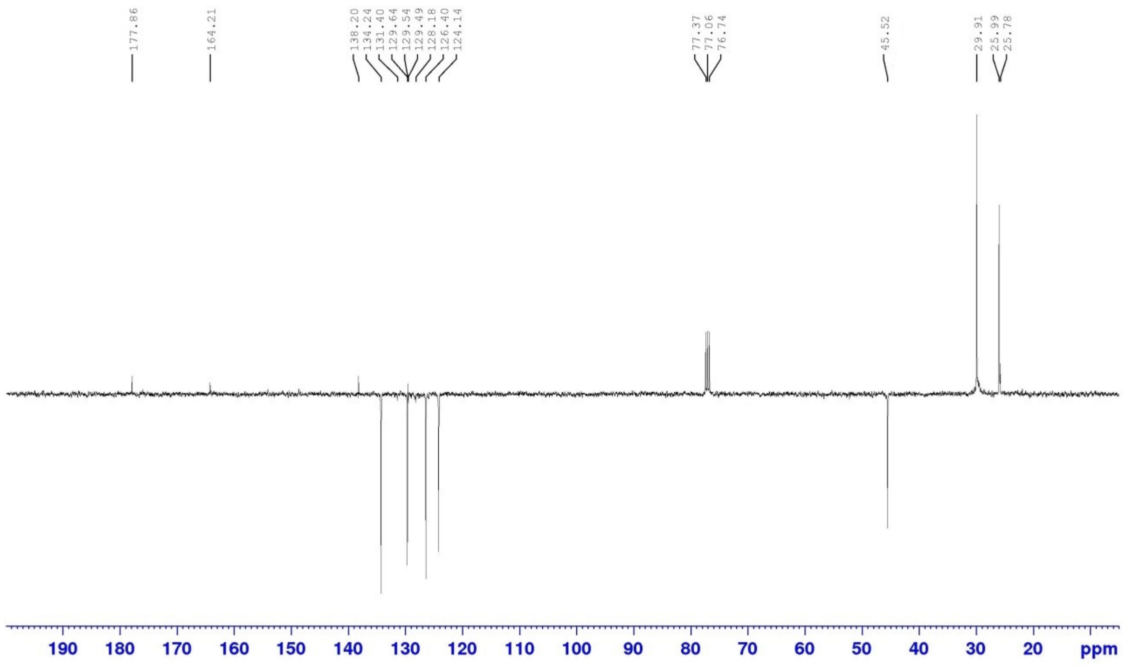

1H NMR Compound 4l
CDCl3

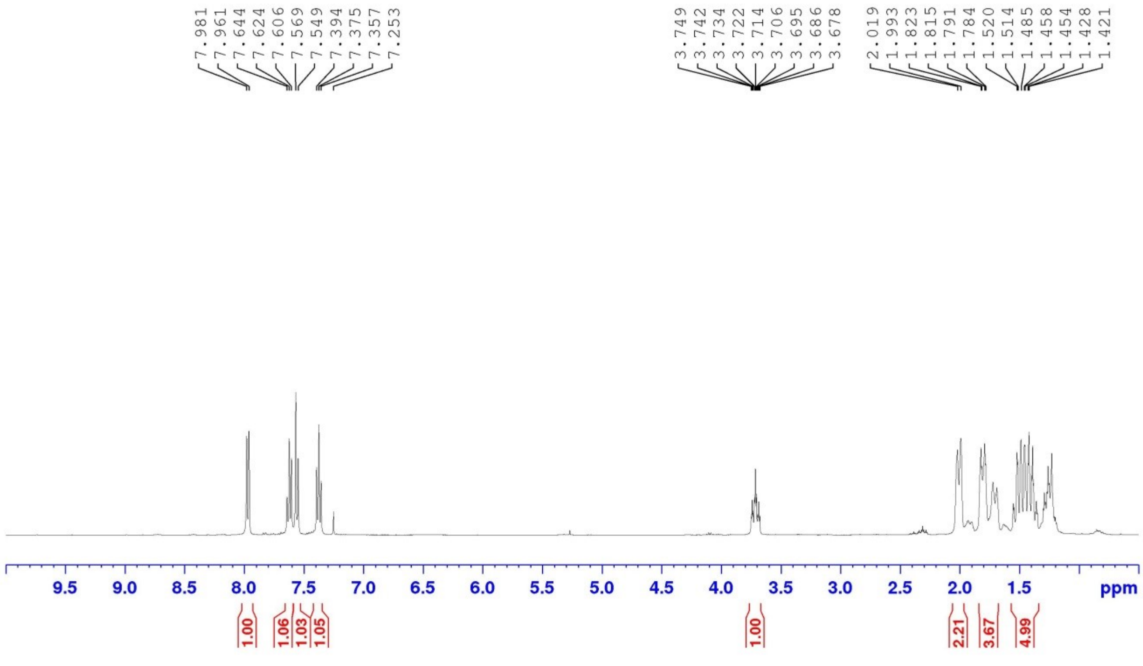

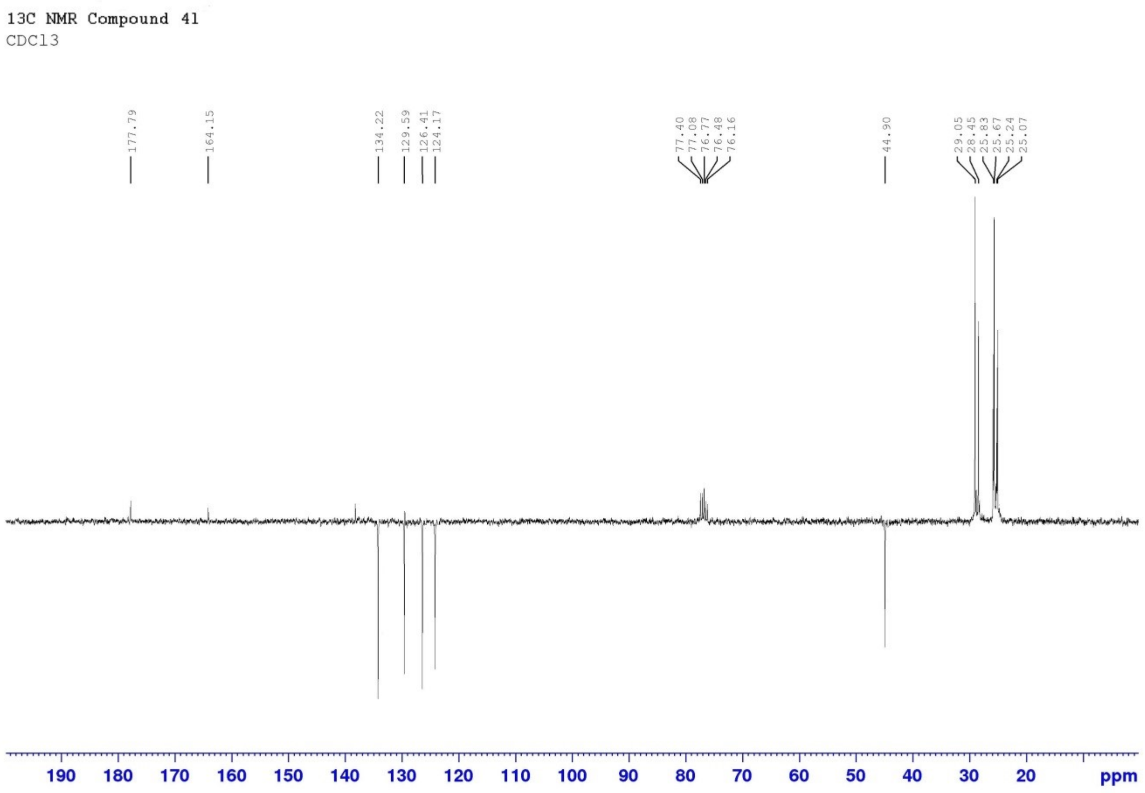

13C NMR Compound 41
CDCl3
177.79
164.15
134.22
129.59
126.41
124.17
77.40
77.08
76.77
76.48
76.16
44.90
29.05
28.45
25.83
25.67
25.24
25.07
190
180
170
160
150
140
130
120
110
100
90
80
70
60
50
40
30
20
ppm


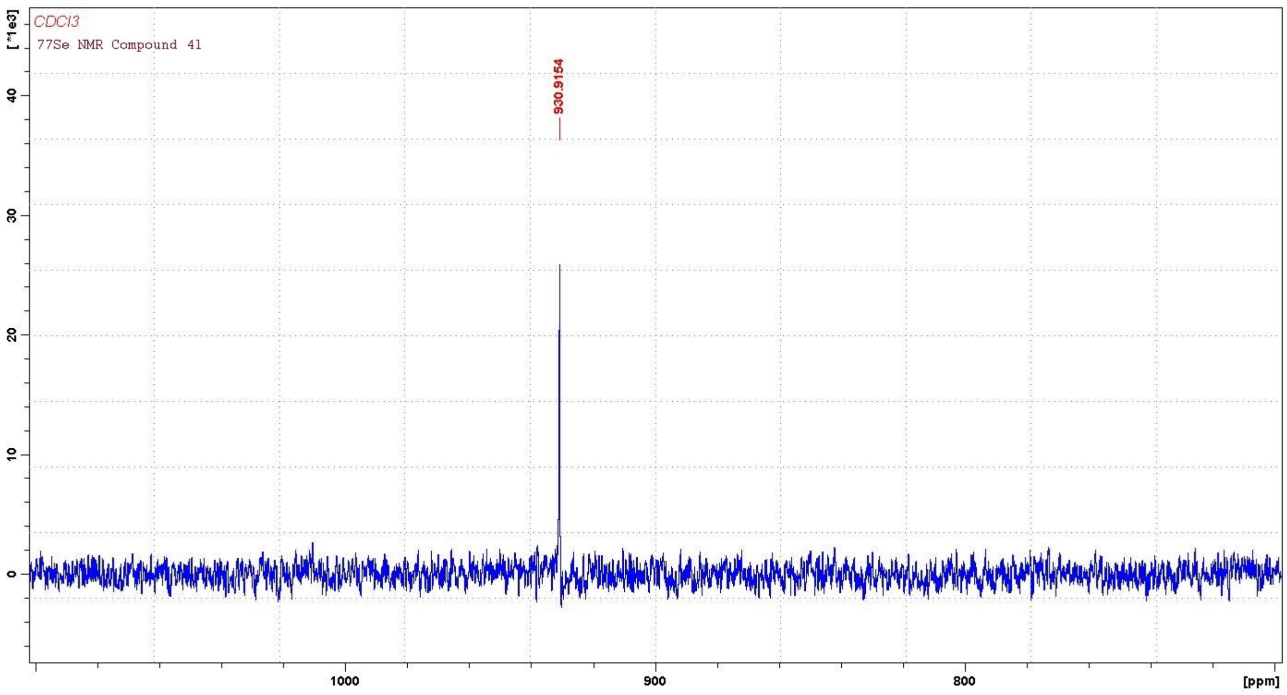

CDCl3
77Se NMR Compound 41
930.9154
[*1e3]
40
30
20
10
0
1000
900
800
[ppm]

1H NMR Compound 4n
CDCl3

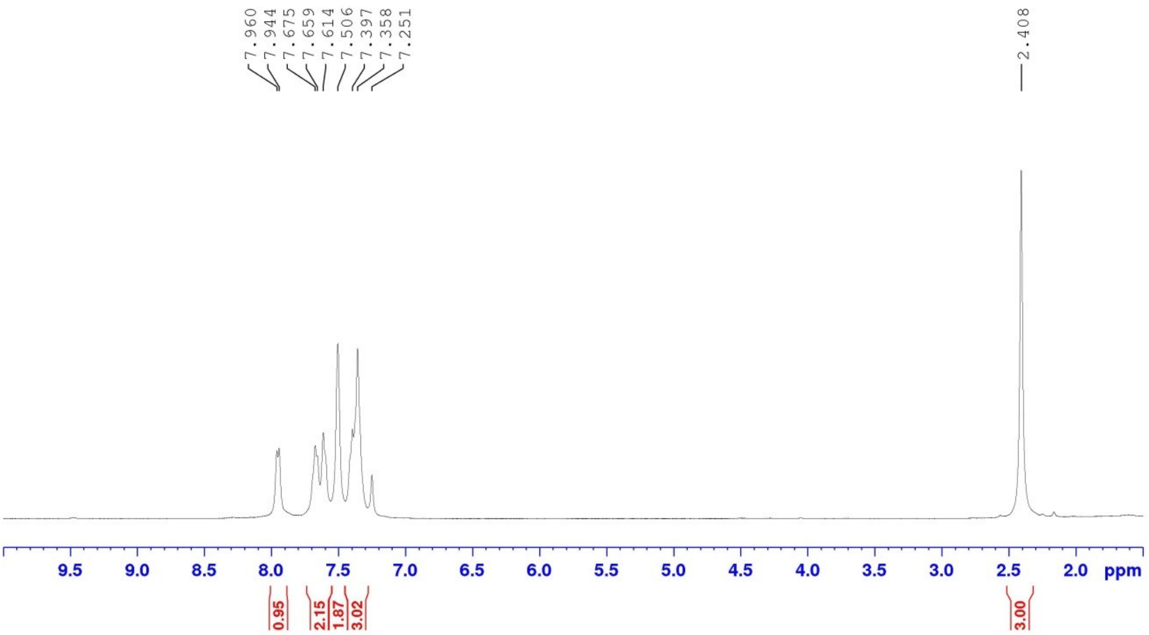


13C NMR Compound 4n
CDCl3

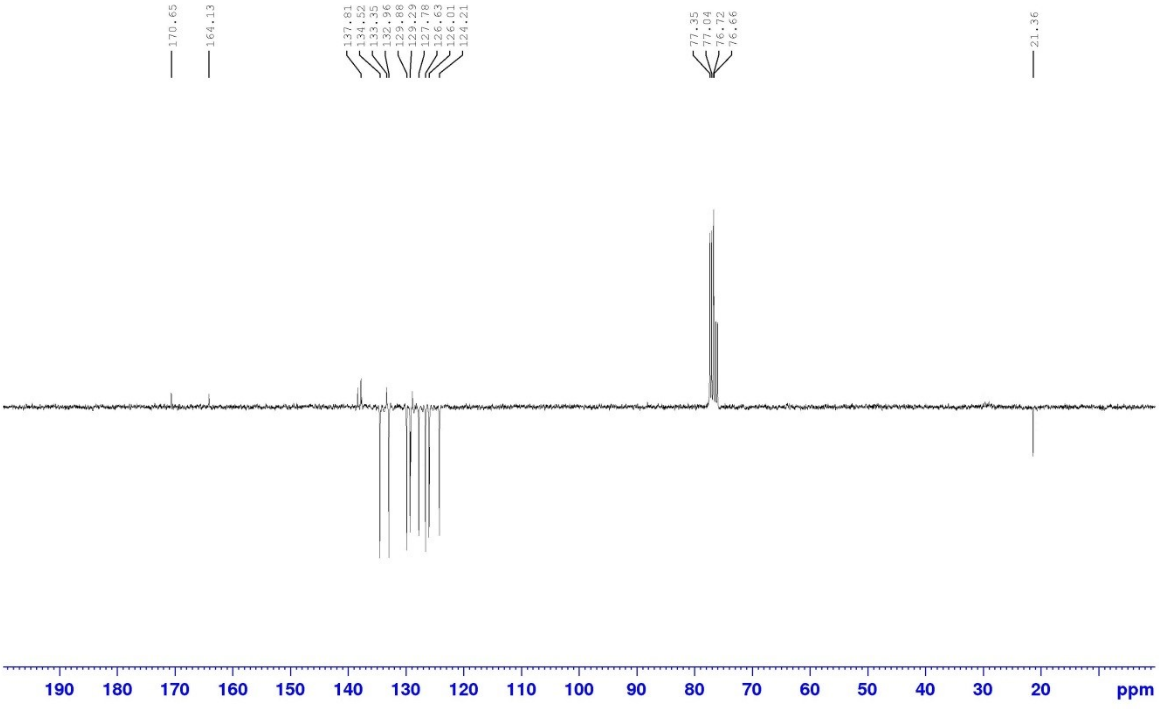

1H NMR Compound 6
DMSO

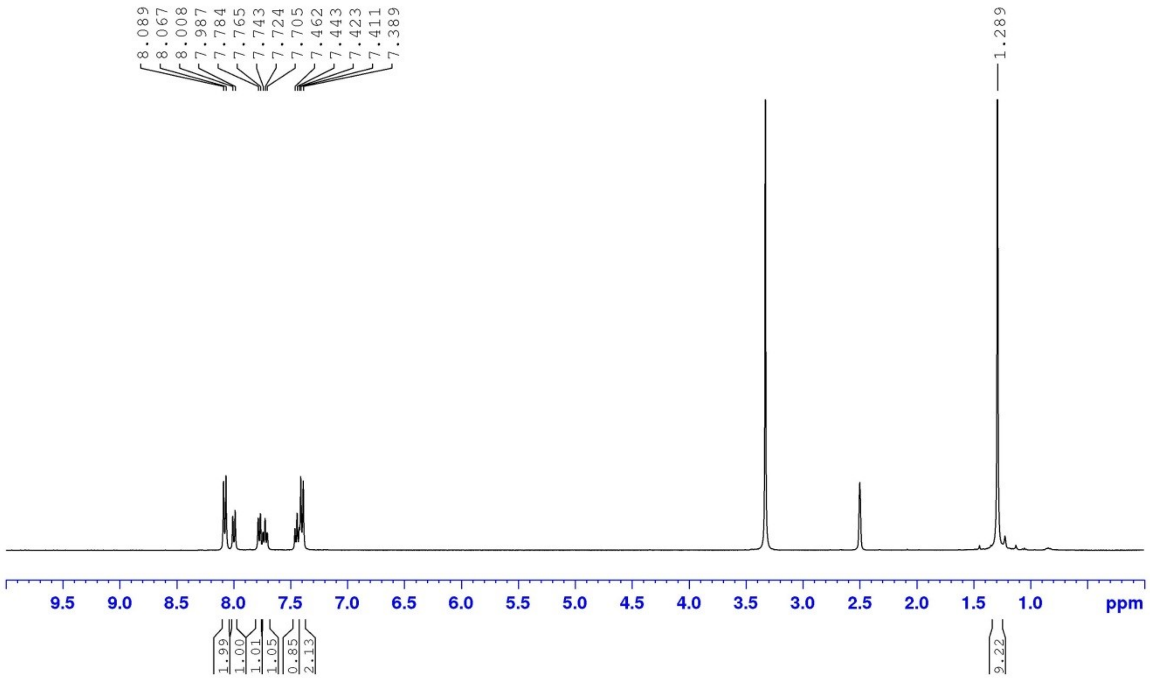


13C NMR Compound 6
DMSO

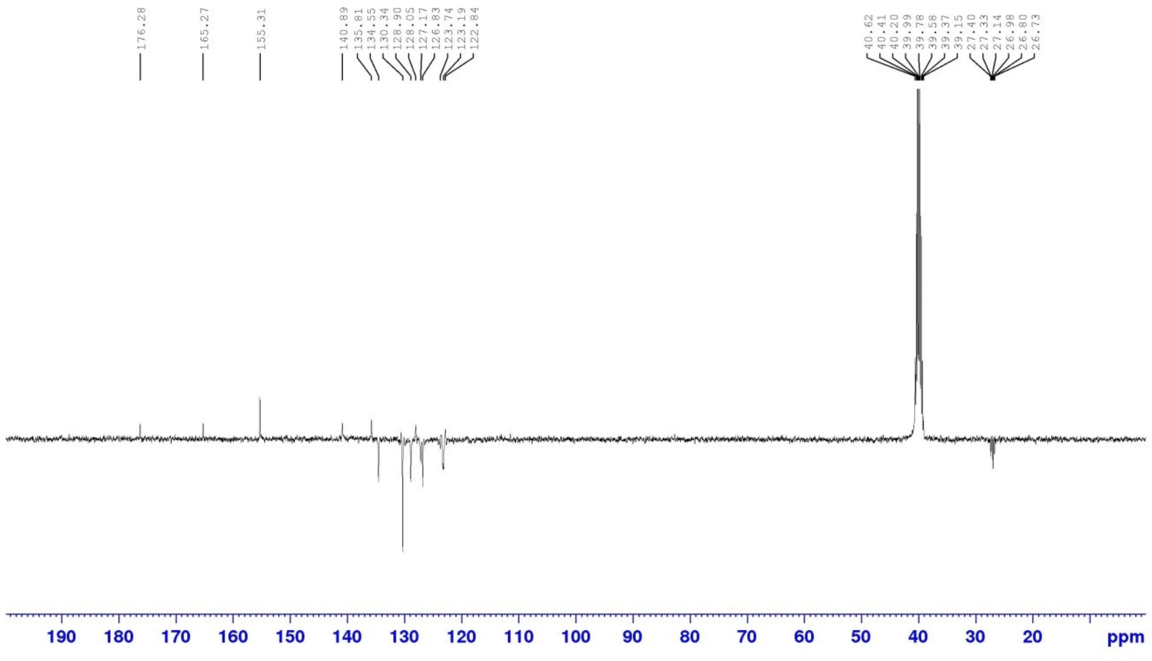

1H NMR Compound 8
DMSO

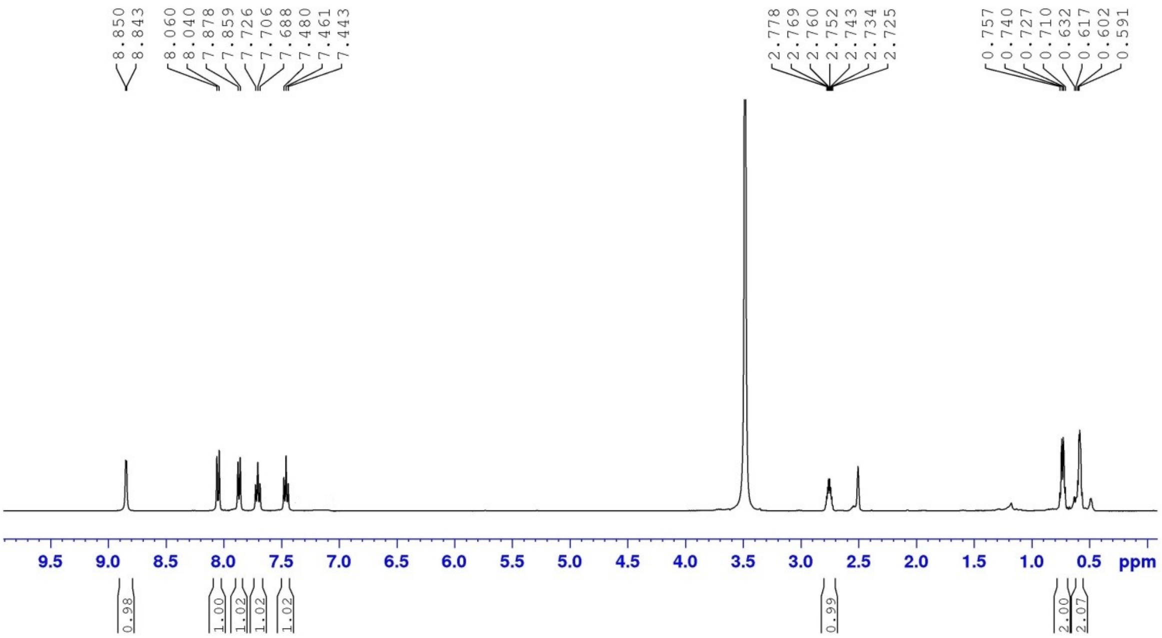


13C NMR Compound 8
DMSO

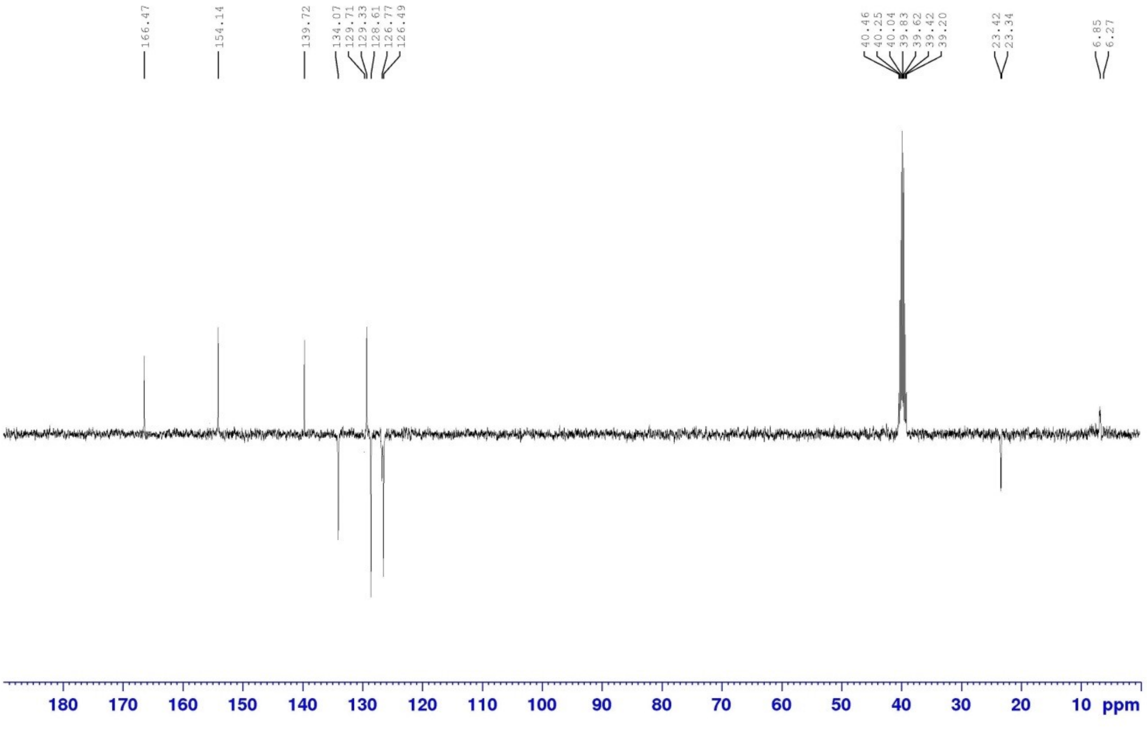

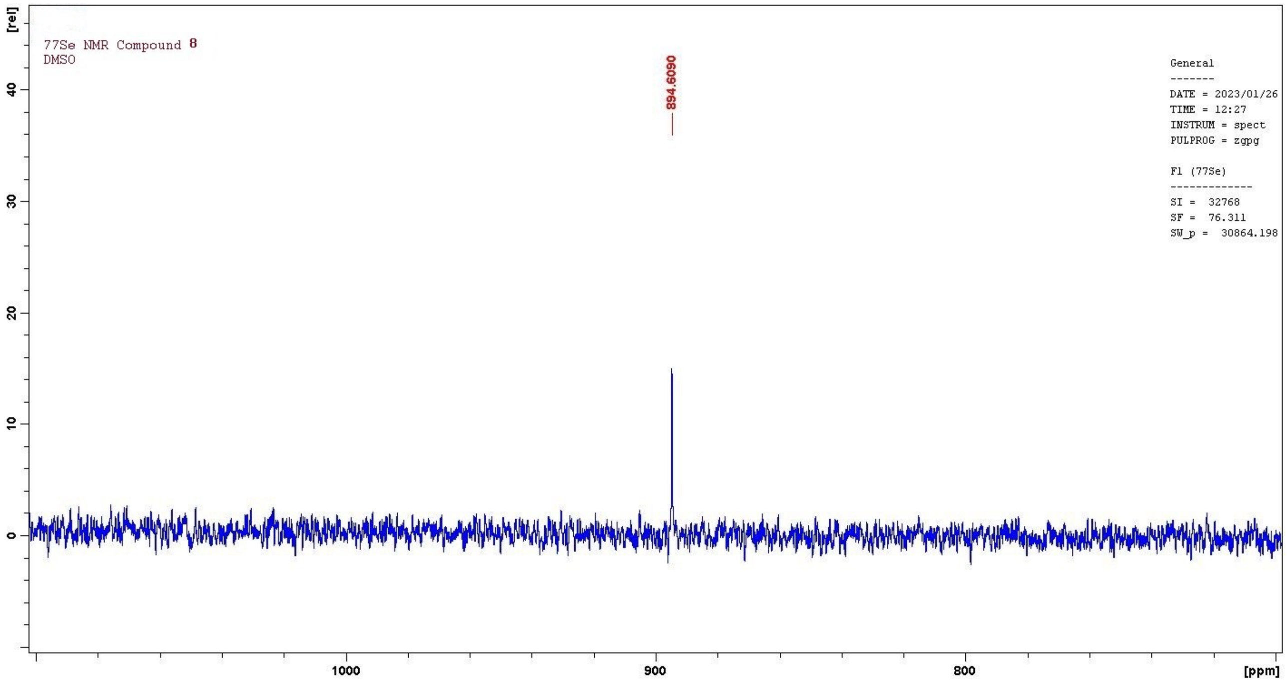
77Se NMR Compound 8
DMSO
894.6090
General
-------
DATE = 2023/01/26
TIME = 12:27
INSTRUM = spect
PULPROG = zgpg
F1 (77Se)
-------------
SI = 32768
SF = 76.311
SW_p = 30864.198
[rel]
[ppm]

*2. Mass Spectrometry spectra*

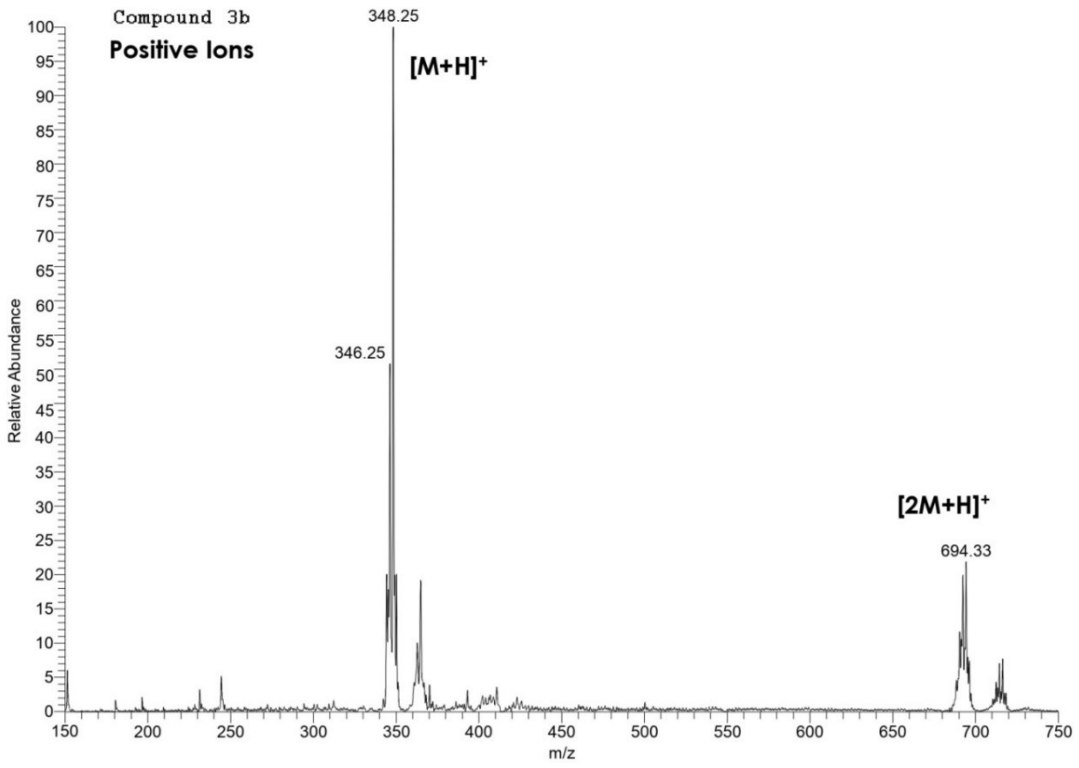


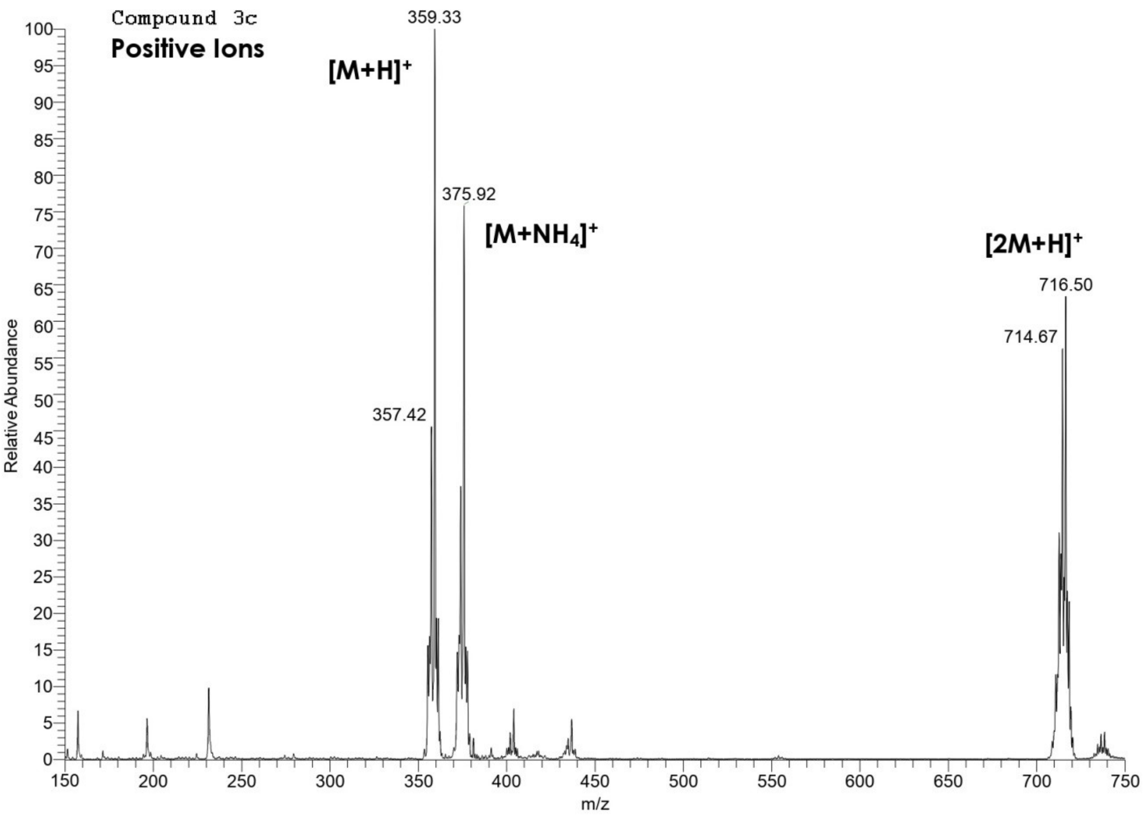

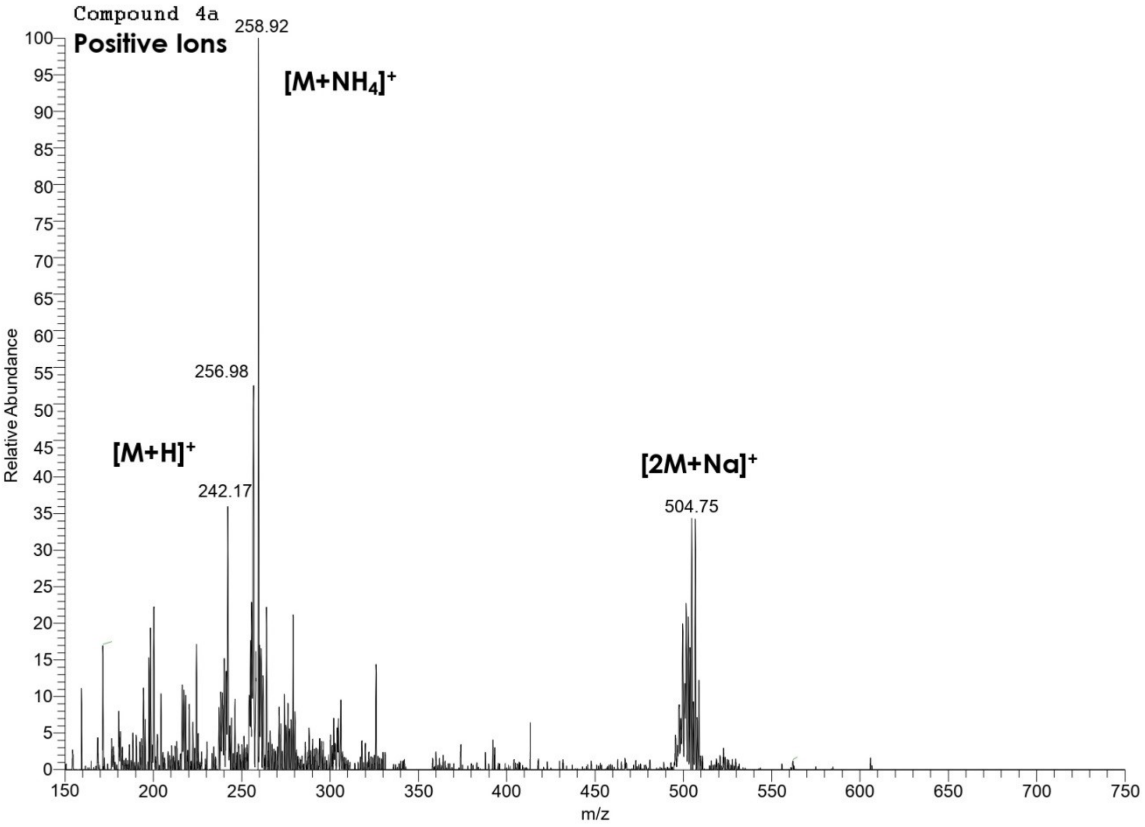


4b #903-914 T: ITMS + p ESI Full ms [150.00-750.00]

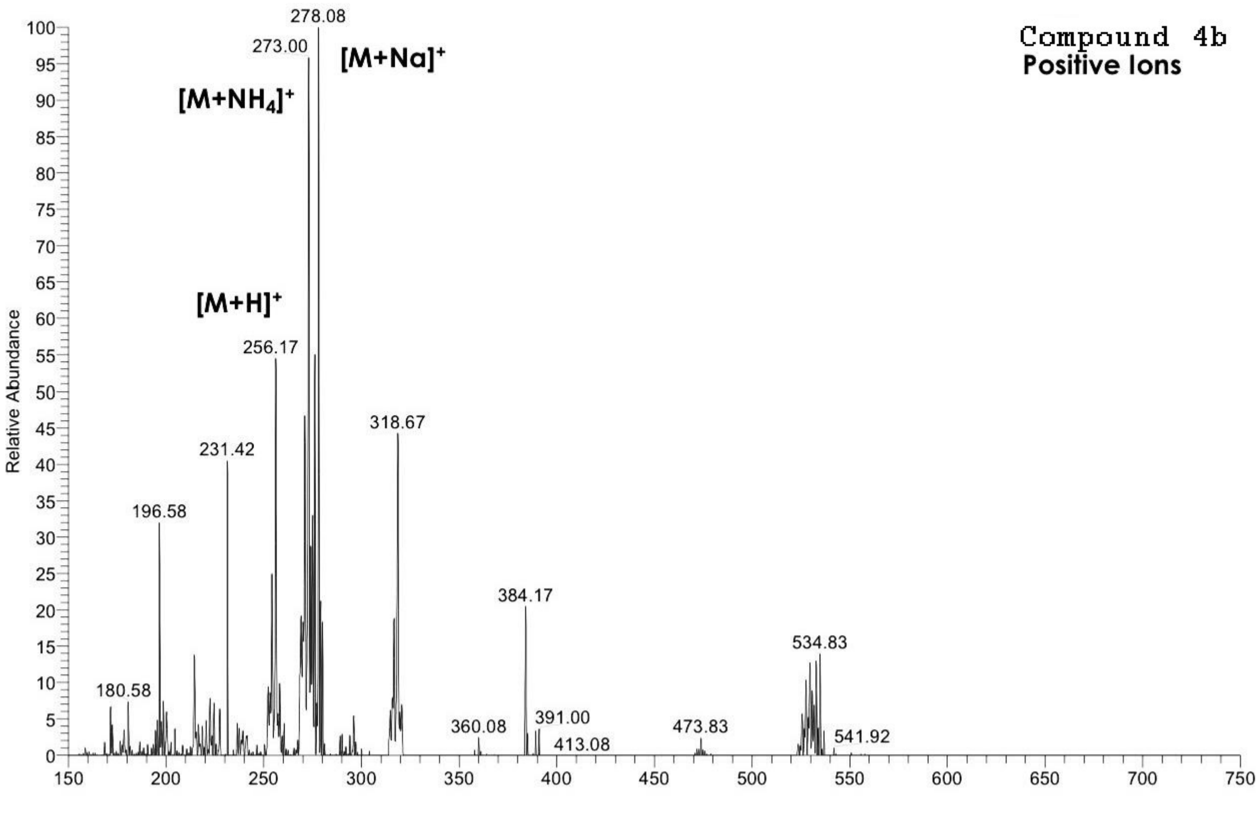

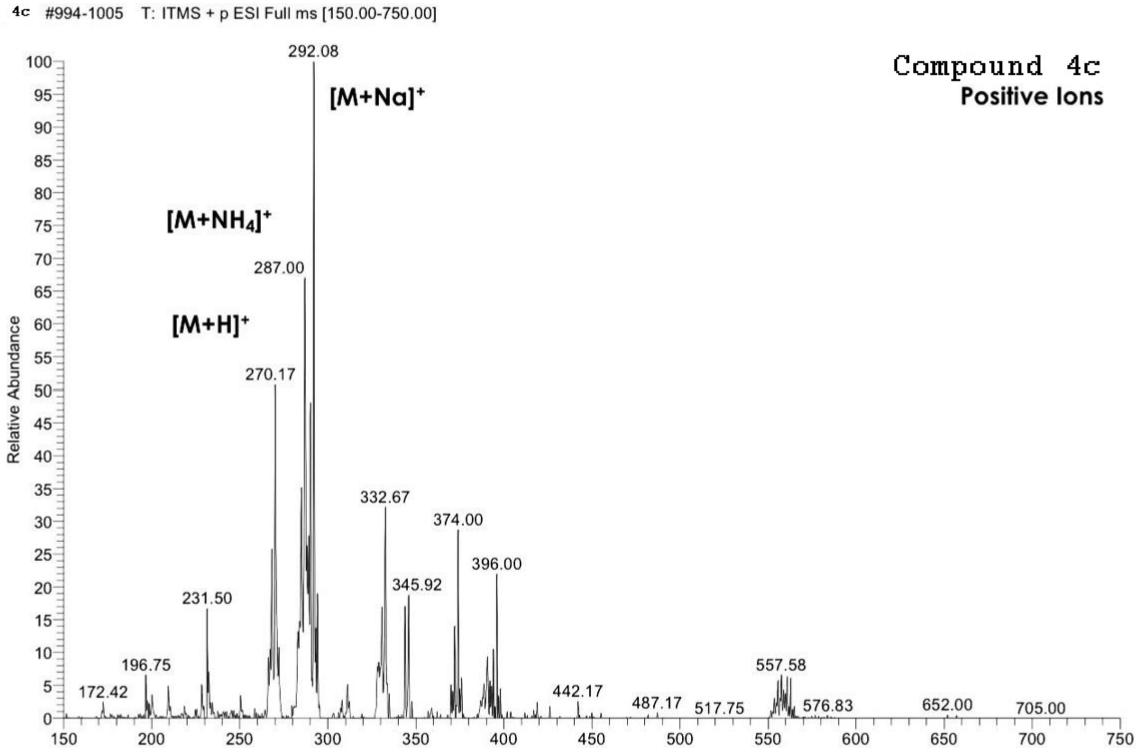
4c #994-1005 T: ITMS + p ESI Full ms [150.00-750.00]
Compound 4c
Positive Ions
[M+Na]+
[M+NH4]+
[M+H]+
292.08
287.00
270.17
332.67
374.00
396.00
345.92
231.50
196.75
172.42
442.17
487.17
517.75
557.58
576.83
652.00
705.00
Relative Abundance

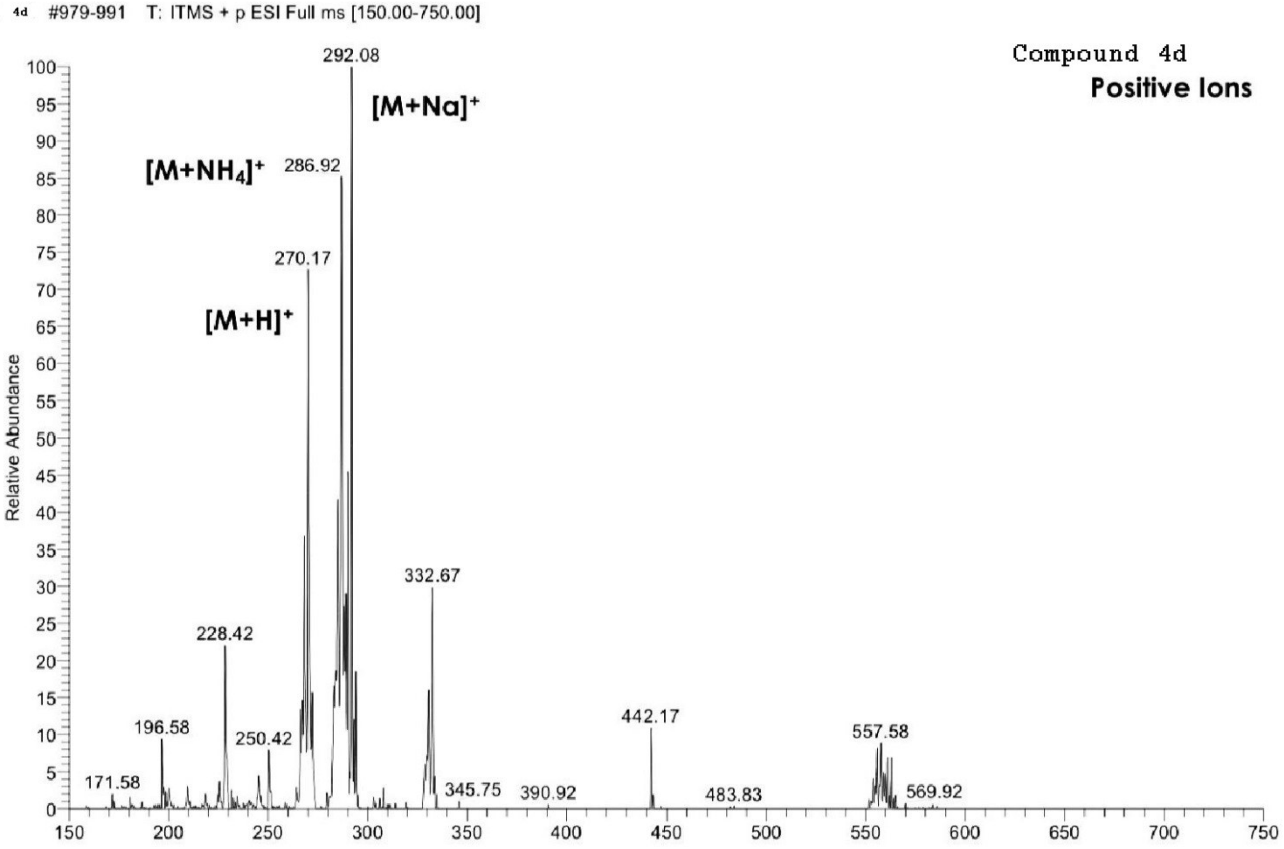
4d #979-991 T: ITMS + p ESI Full ms [150.00-750.00]
Compound 4d
Positive Ions
[M+Na]+
[M+NH4]+
[M+H]+
292.08
286.92
270.17
332.67
228.42
196.58
250.42
171.58
345.75
390.92
442.17
483.83
557.58
569.92
Relative Abundance

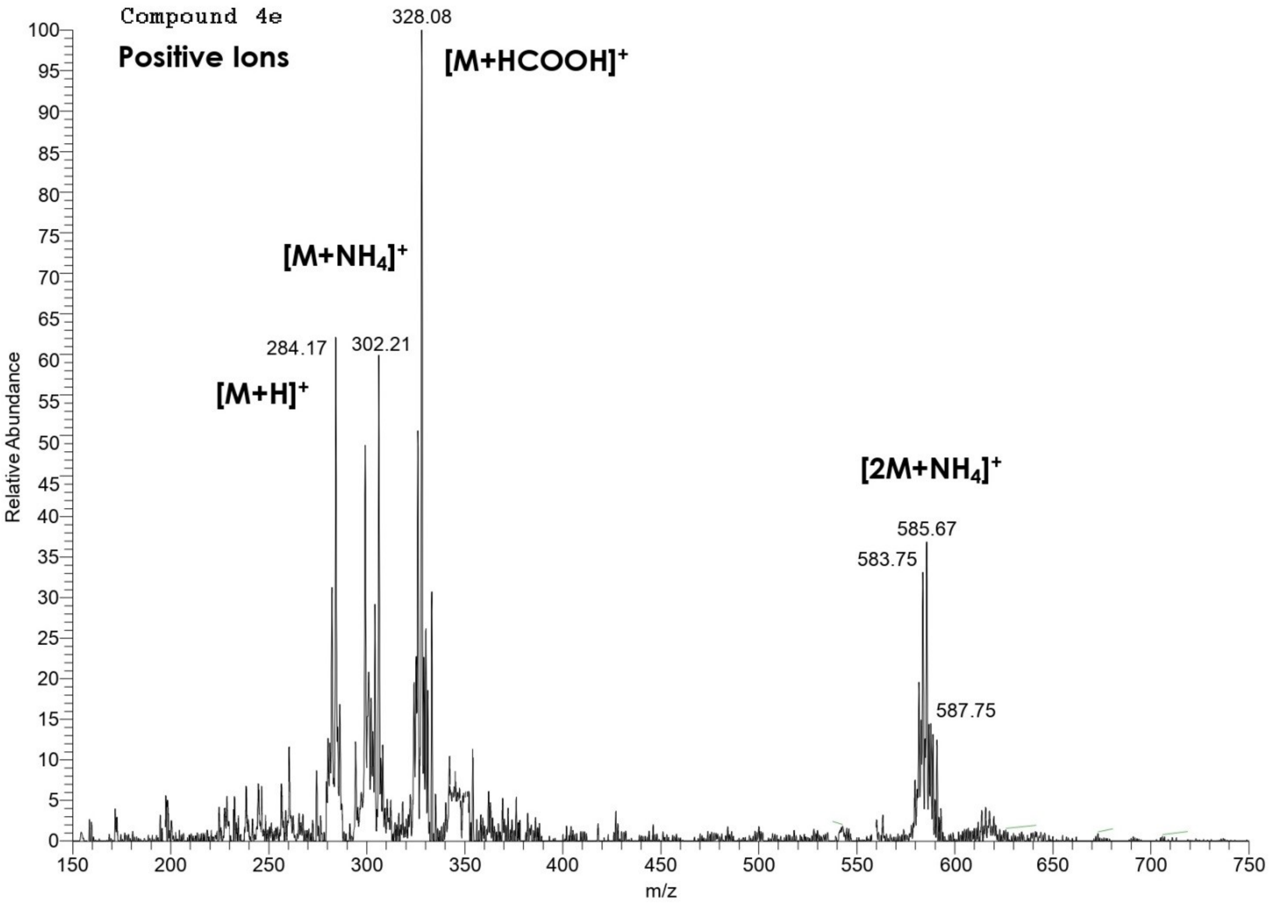
Compound 4e
Positive Ions
328.08
[M+HCOOH]+
[M+NH4]+
284.17
302.21
[M+H]+
[2M+NH4]+
585.67
583.75
587.75
Relative Abundance
m/z

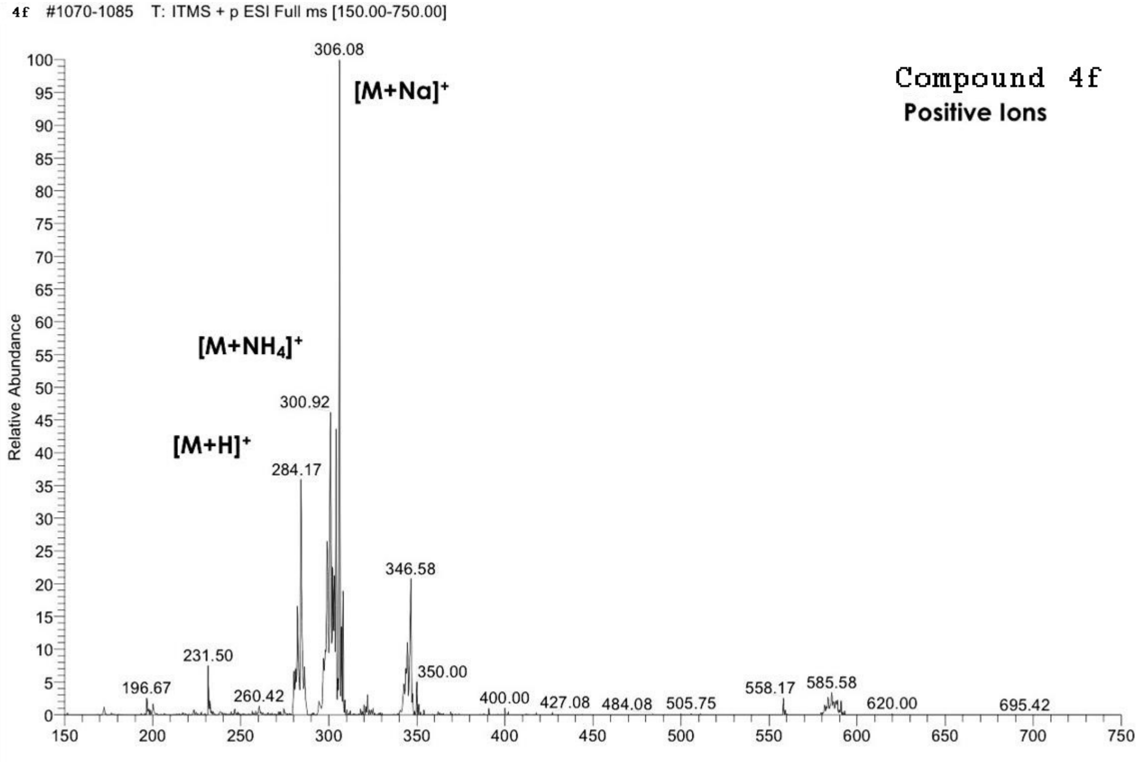
4f #1070-1085 T: ITMS + p ESI Full ms [150.00-750.00]
306.08
[M+Na]+
Compound 4f
Positive Ions
[M+NH4]+
300.92
[M+H]+
284.17
346.58
231.50
350.00
196.67
260.42
400.00
427.08
484.08
505.75
558.17
585.58
620.00
695.42
Relative Abundance

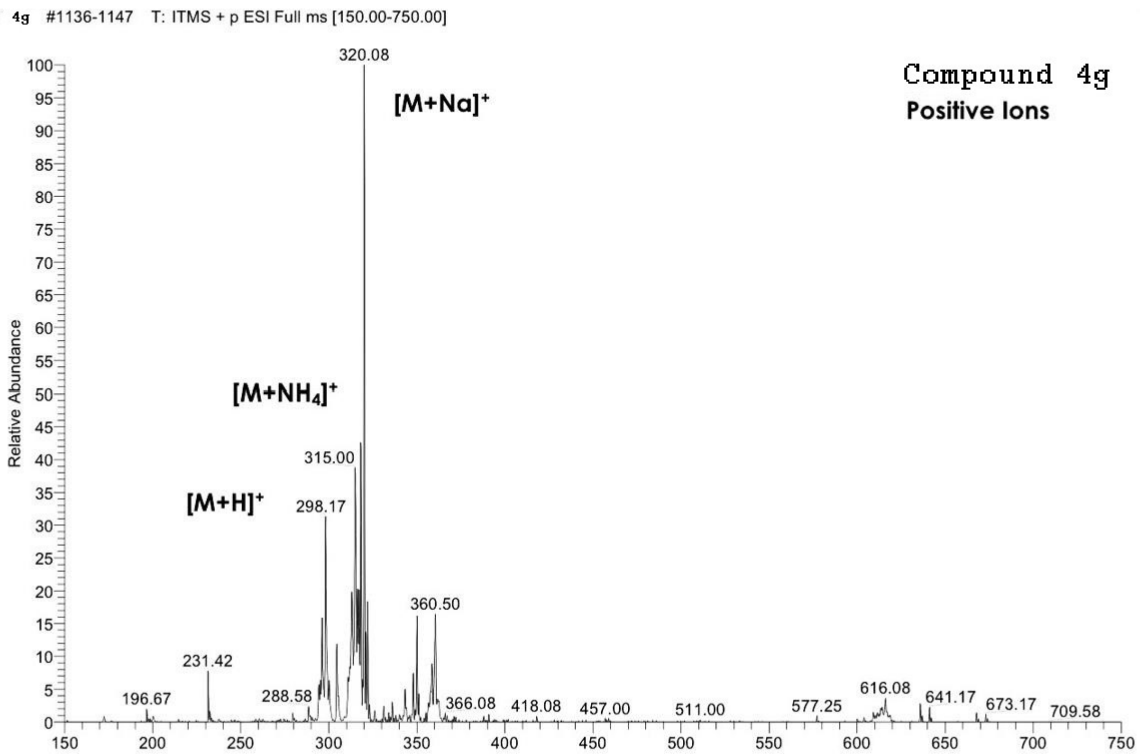
4g #1136-1147 T: ITMS + p ESI Full ms [150.00-750.00]
Compound 4g
Positive Ions
[M+Na]+
[M+NH4]+
[M+H]+
320.08
315.00
298.17
360.50
231.42
196.67
288.58
366.08
418.08
457.00
511.00
577.25
616.08
641.17
673.17
709.58
Relative Abundance

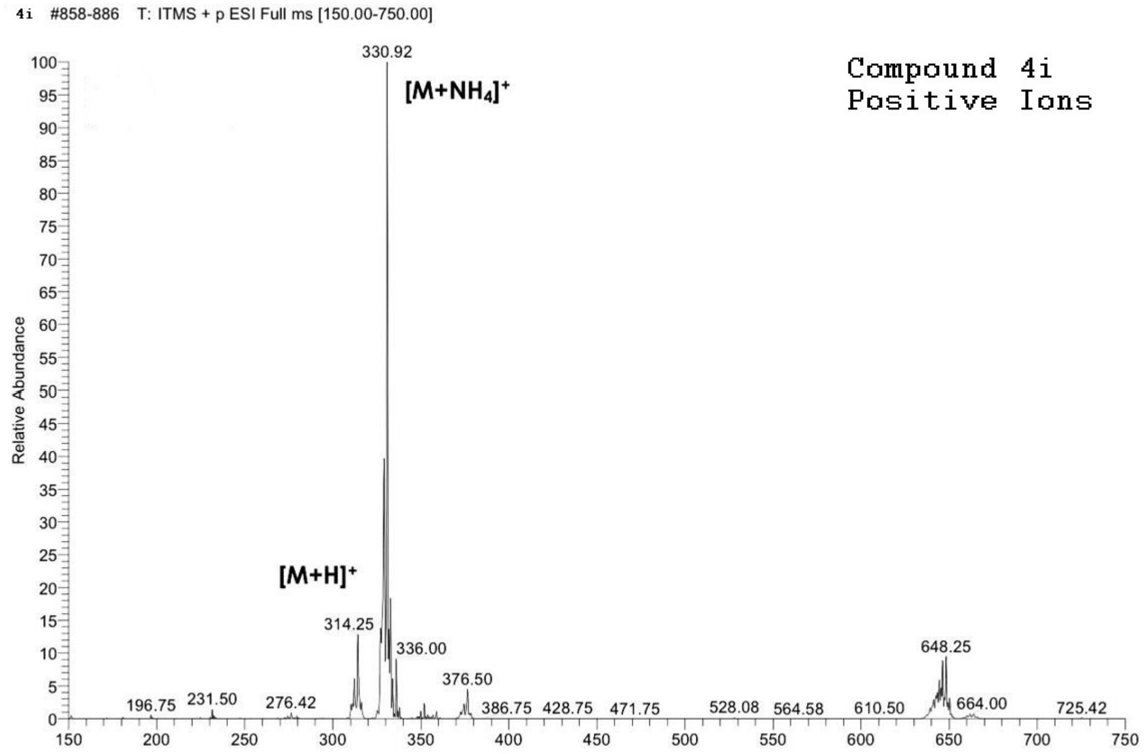
4i #858-886 T: ITMS + p ESI Full ms [150.00-750.00]
Compound 4i
Positive Ions
[M+NH4]+
[M+H]+
330.92
314.25
336.00
376.50
196.75
231.50
276.42
386.75
428.75
471.75
528.08
564.58
610.50
648.25
664.00
725.42
Relative Abundance

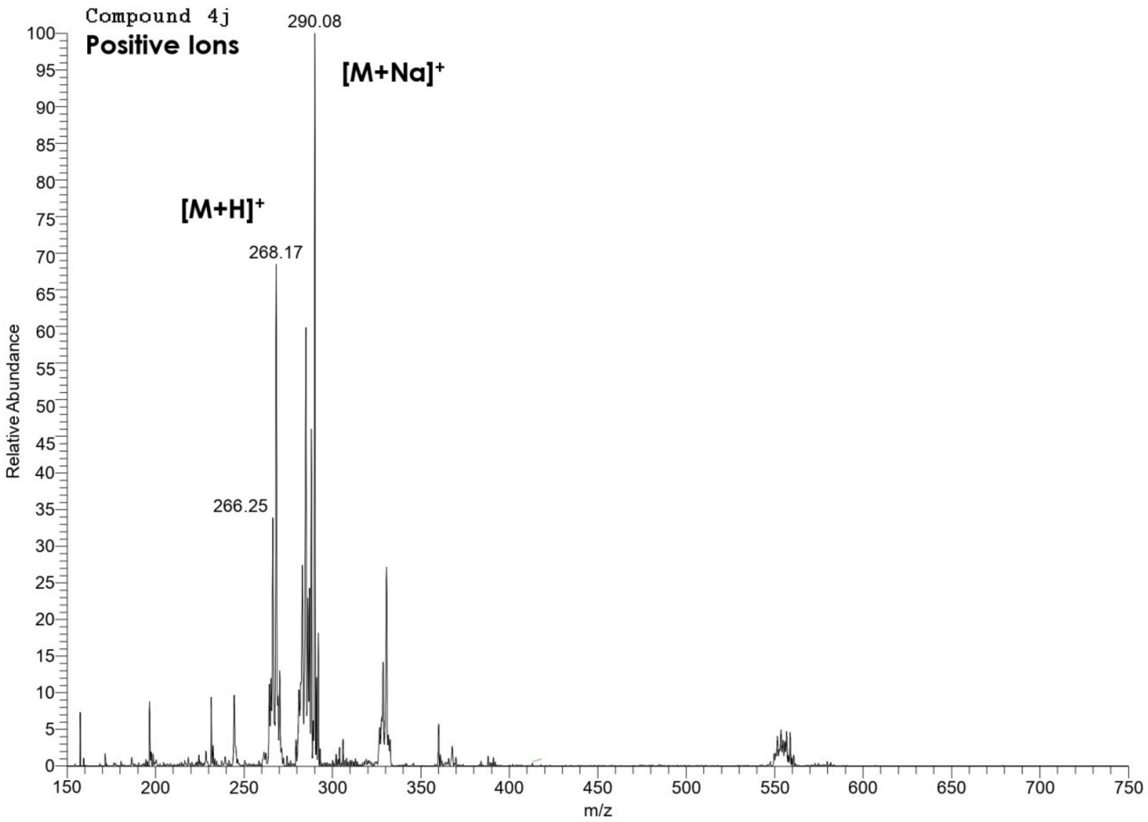
Compound 4j
Positive Ions
290.08
[M+Na]+
[M+H]+
268.17
266.25
Relative Abundance
m/z

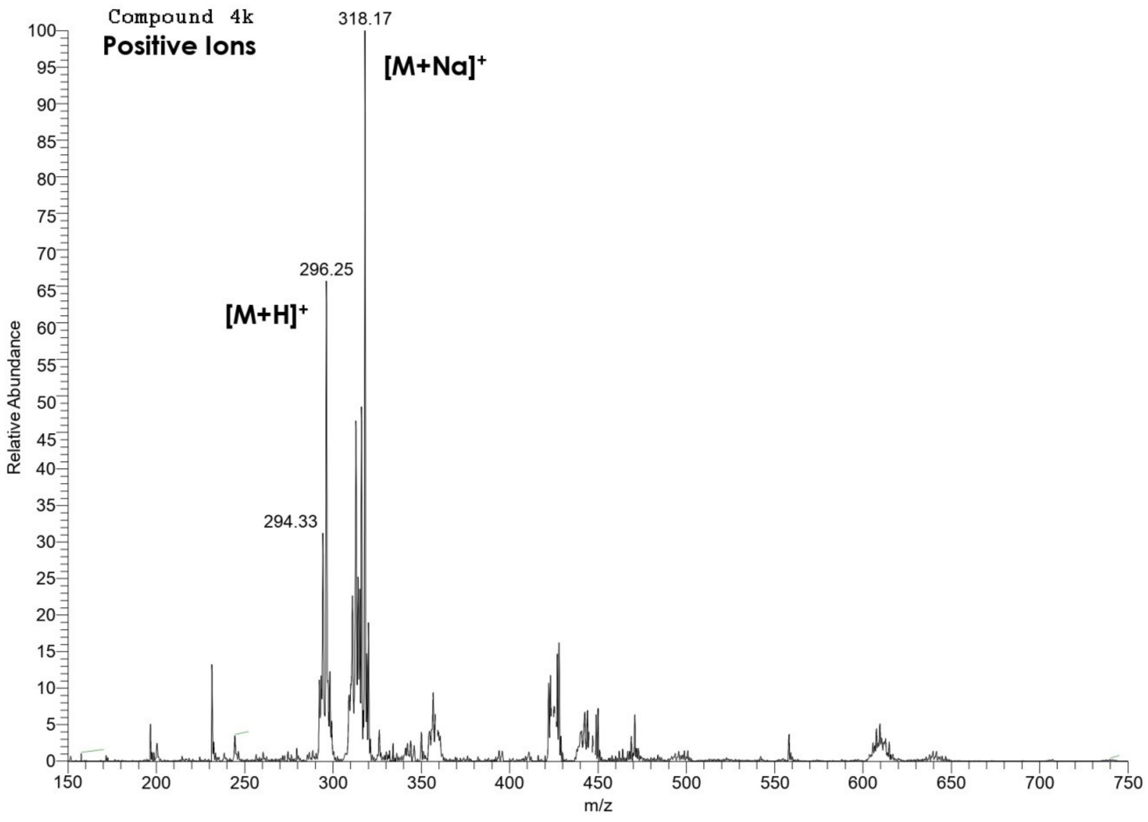
Compound 4k
Positive Ions
318.17
[M+Na]+
296.25
[M+H]+
294.33
Relative Abundance
m/z

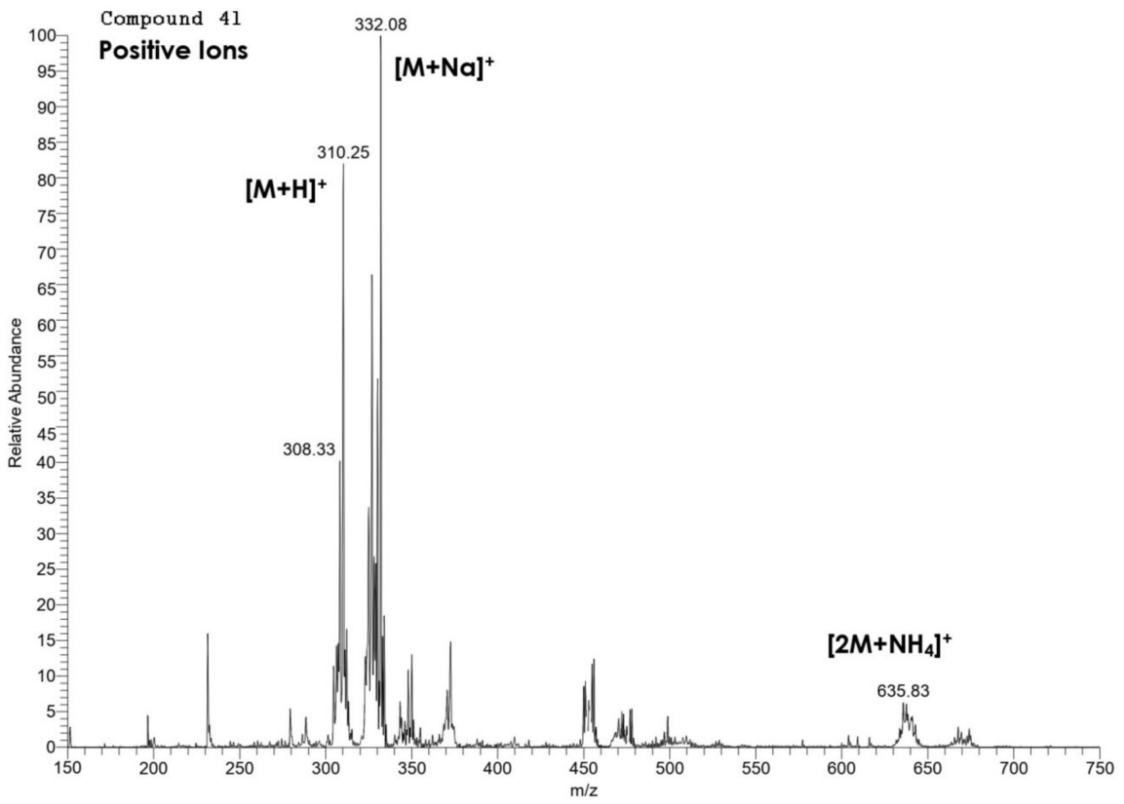
Compound 4l
Positive Ions
332.08
[M+Na]+
310.25
[M+H]+
308.33
[2M+NH4]+
635.83
Relative Abundance
m/z

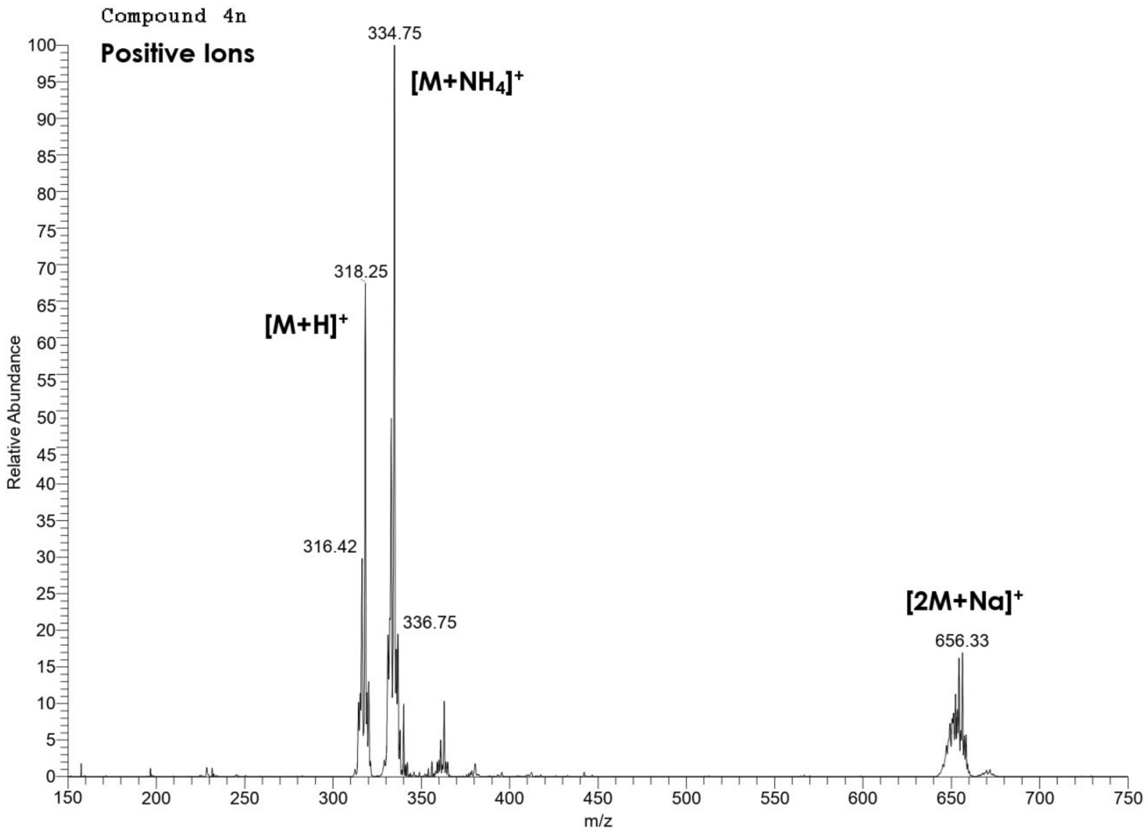
Compound 4n
Positive Ions
334.75
[M+NH4]+
318.25
[M+H]+
316.42
336.75
[2M+Na]+
656.33
Relative Abundance
m/z

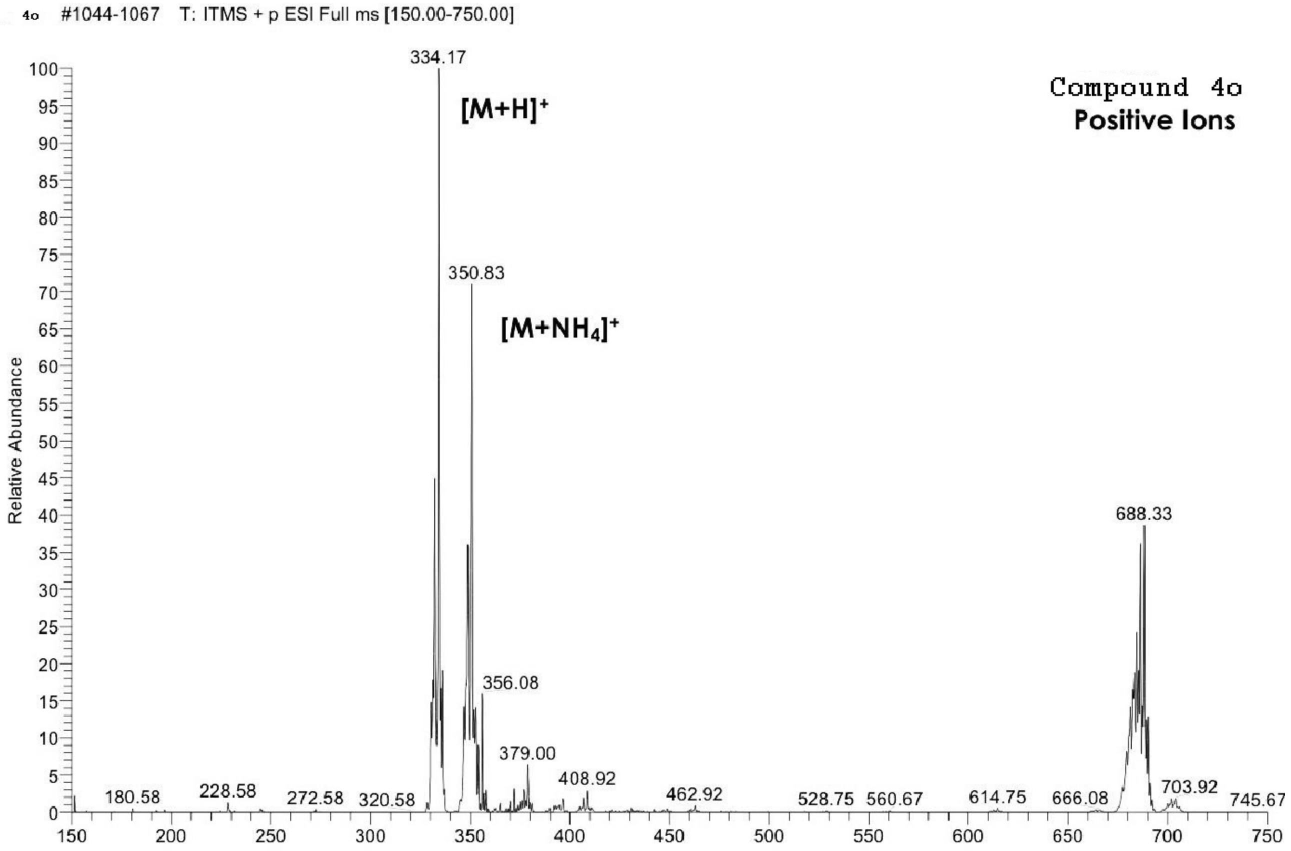
4o #1044-1067 T: ITMS + p ESI Full ms [150.00-750.00]
Compound 4o
Positive Ions
334.17
[M+H]+
350.83
[M+NH4]+
356.08
379.00
408.92
688.33
180.58
228.58
272.58
320.58
462.92
528.75
560.67
614.75
666.08
703.92
745.67
Relative Abundance

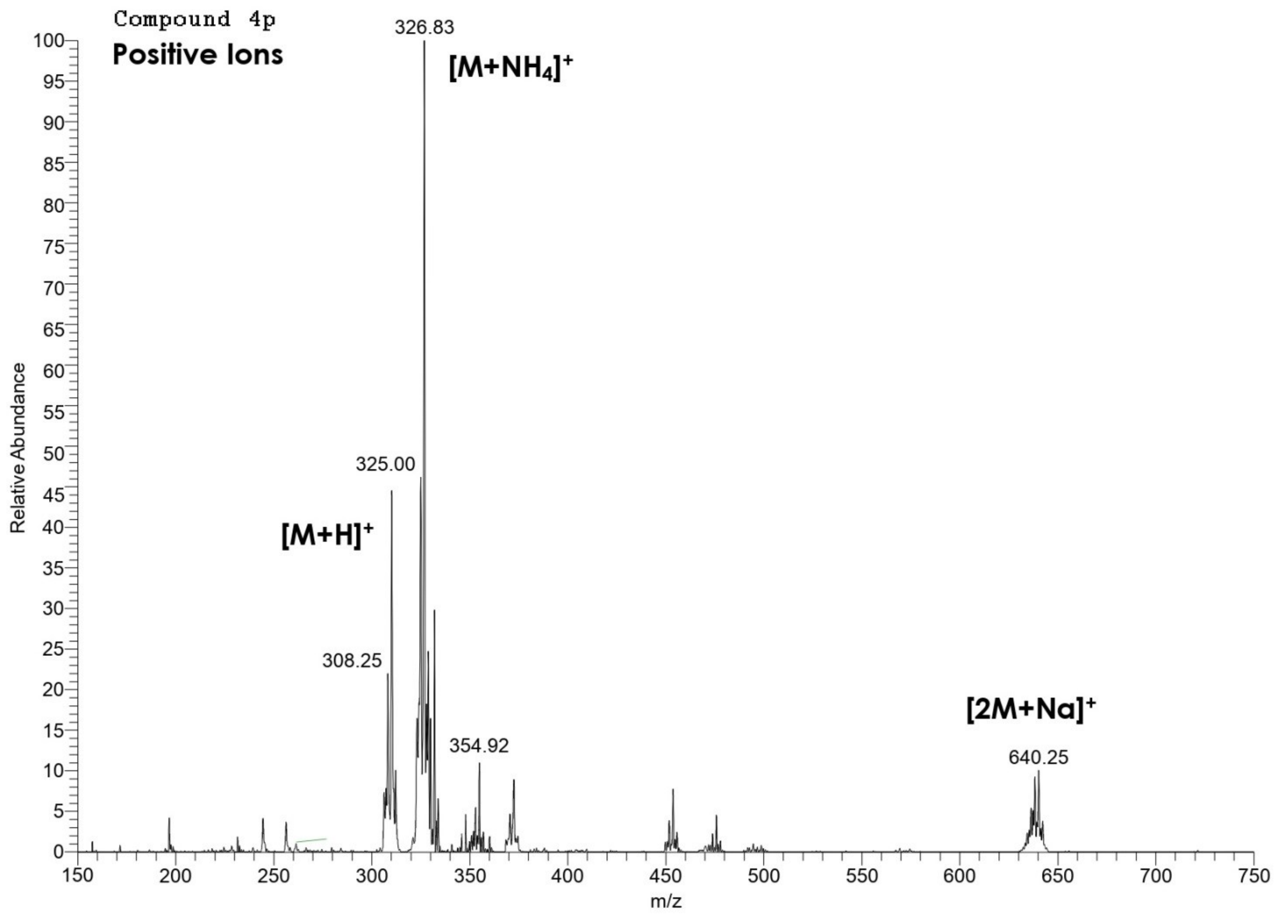
Compound 4p
Positive Ions
326.83
[M+NH4]+
325.00
[M+H]+
308.25
354.92
[2M+Na]+
640.25
Relative Abundance
m/z

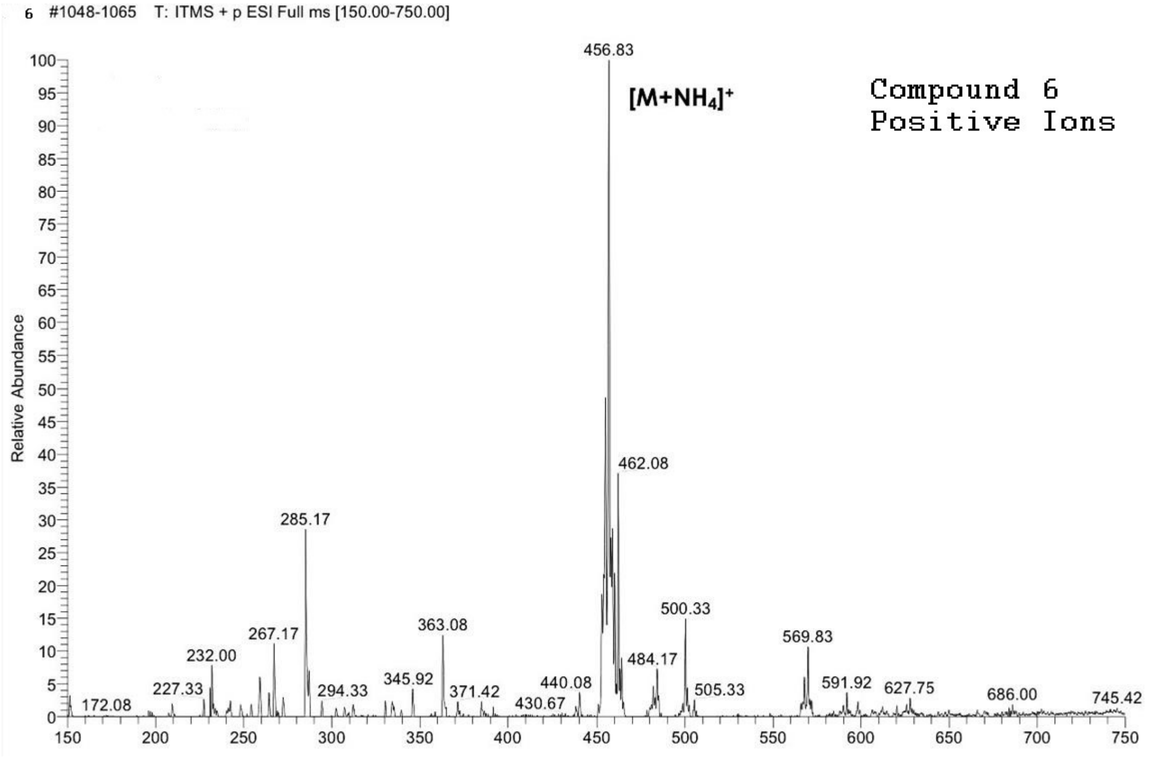
6 #1048-1065 T: ITMS + p ESI Full ms [150.00-750.00]
Compound 6
Positive Ions
[M+NH4]+
456.83
462.08
285.17
500.33
569.83
363.08
267.17
232.00
484.17
227.33
345.92
294.33
371.42
440.08
430.67
505.33
591.92
627.75
686.00
745.42
172.08
Relative Abundance

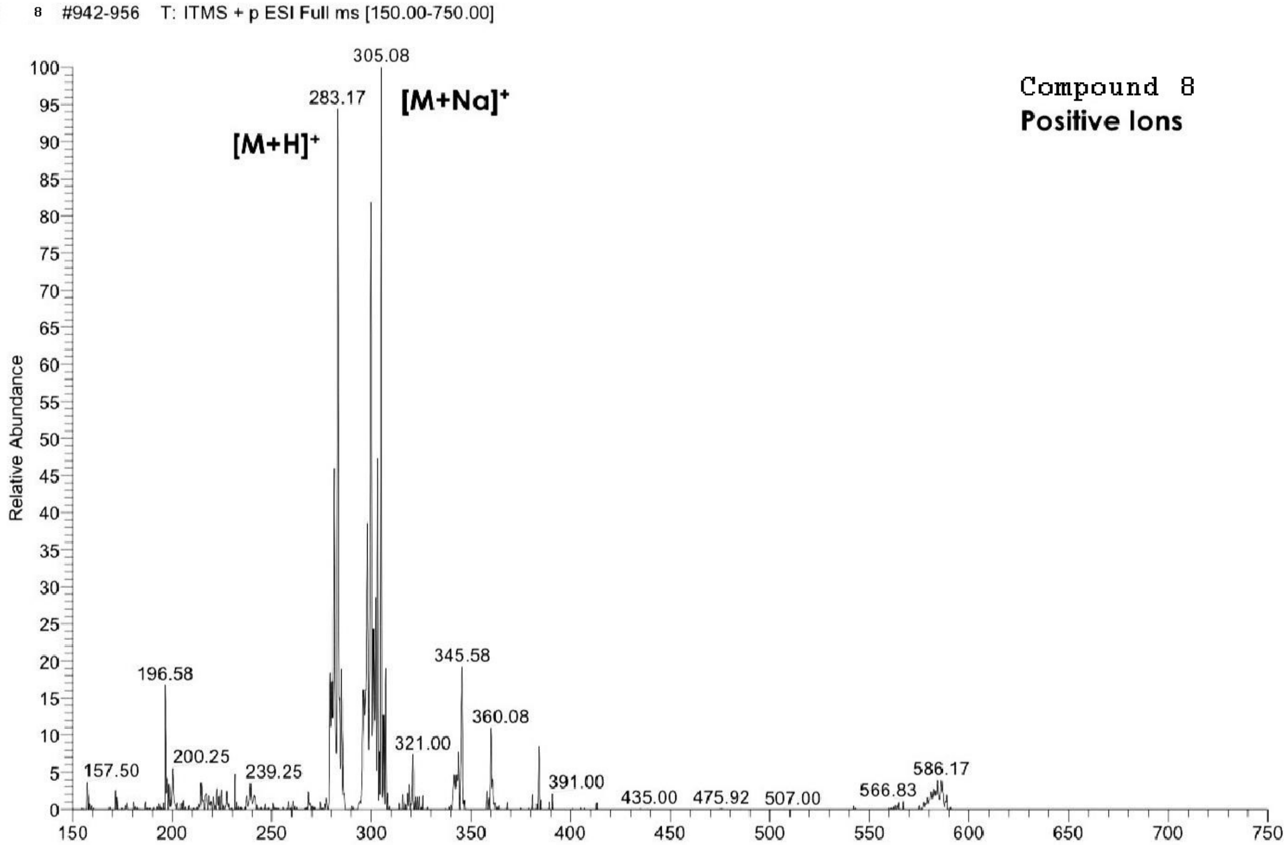
8 #942-956 T: ITMS + p ESI Full ms [150.00-750.00]
Compound 8
Positive Ions
305.08
283.17
[M+Na]+
[M+H]+
196.58
345.58
360.08
321.00
200.25
239.25
157.50
391.00
435.00
475.92
507.00
566.83
586.17
Relative Abundance

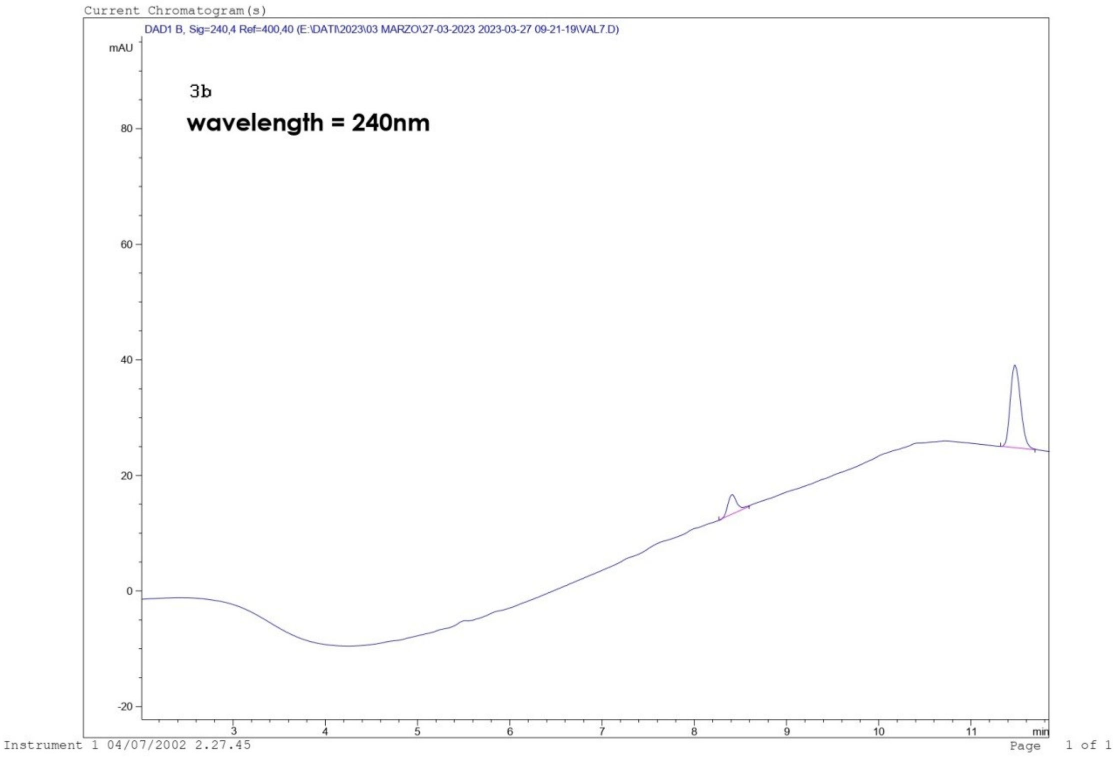
Current Chromatogram(s)
DAD1 B, Sig=240,4 Ref=400,40 (E:\DATI\2023\03 MARZO\27-03-2023 2023-03-27 09-21-19\VAL7.D)
mAU
3b
wavelength = 240nm
80
60
40
20
0
-20
3
4
5
6
7
8
9
10
11
min
Instrument 1 04/07/2002 2.27.45
Page 1 of 1

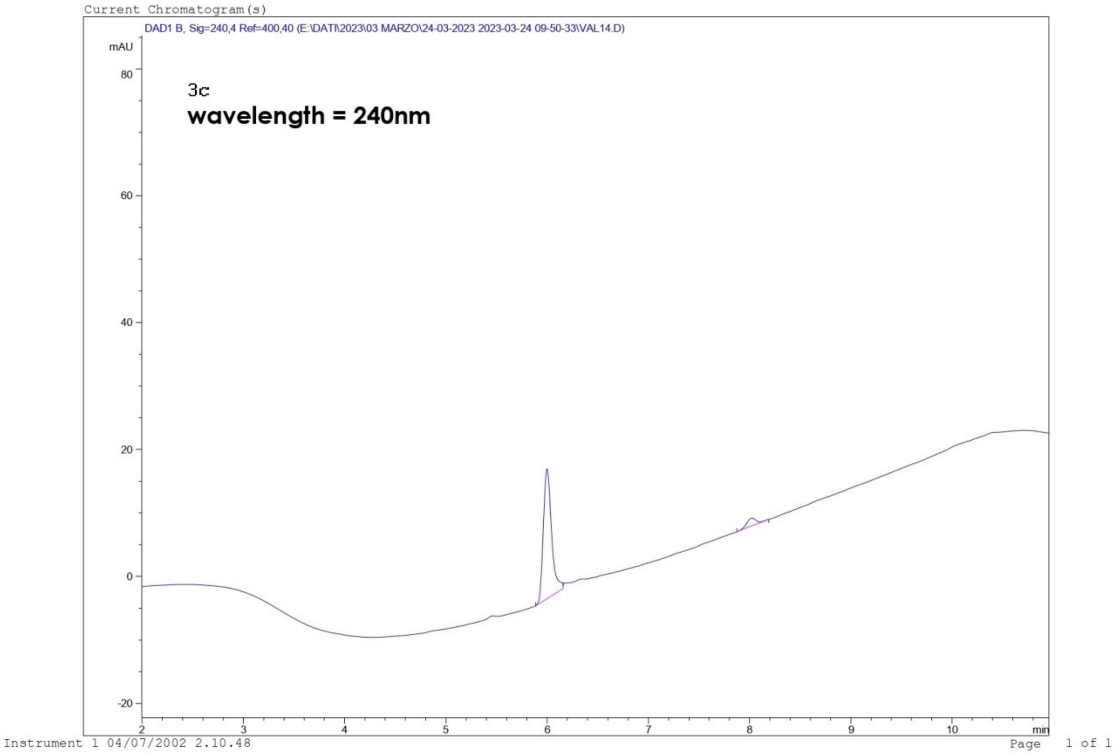
Current Chromatogram(s)
DAD1 B, Sig=240,4 Ref=400,40 (E:\DATI\2023\03 MARZO\24-03-2023 2023-03-24 09-50-33\VAL14.D)
mAU
80
3c
wavelength = 240nm
60
40
20
0
-20
2
3
4
5
6
7
8
9
10
min
Instrument 1 04/07/2002 2.10.48
Page 1 of 1

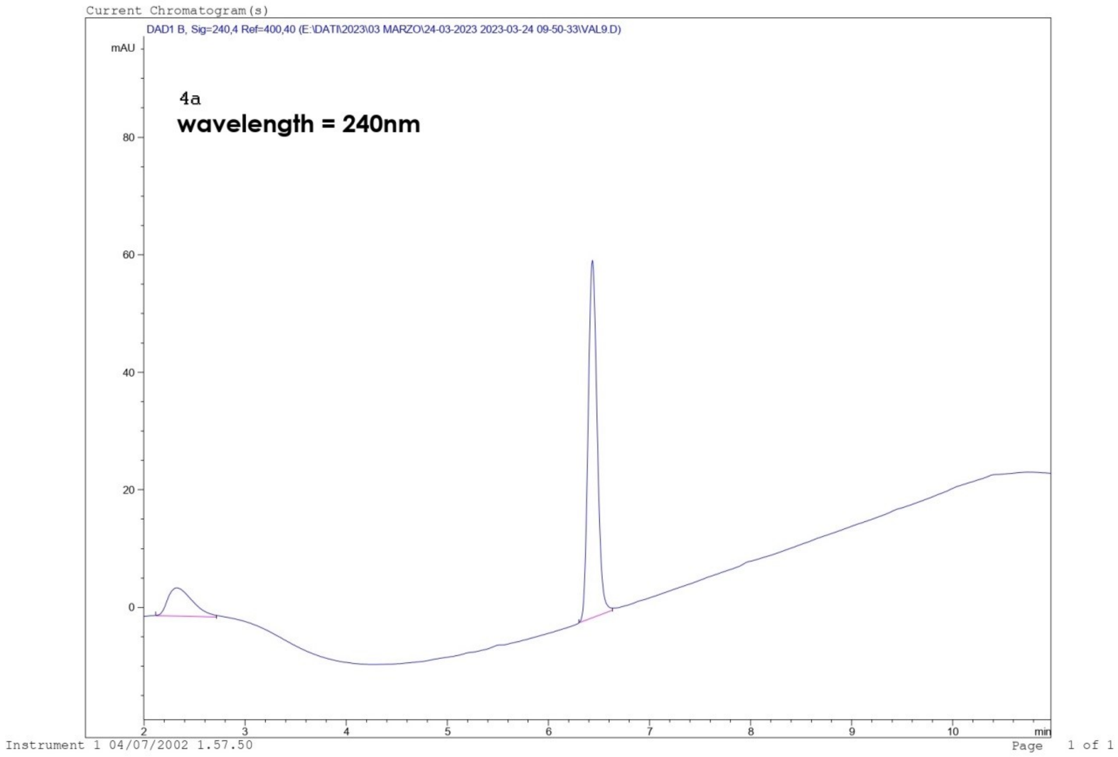
Current Chromatogram(s)
DAD1 B, Sig=240,4 Ref=400,40 (E:\DATI\2023\03 MARZO\24-03-2023 2023-03-24 09-50-33\VAL9.D)
mAU
4a
wavelength = 240nm
min
Instrument 1 04/07/2002 1.57.50
Page 1 of 1

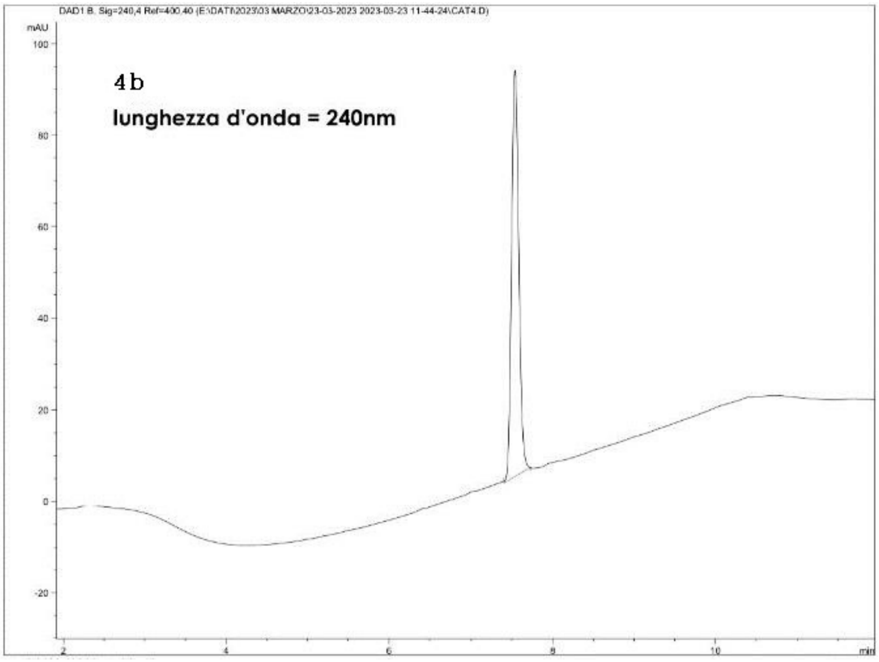
DAD1 B, Sig=240,4 Ref=400,40 (E:\DATI\2023\03 MARZO\23-03-2023 2023-03-23 11-44-24\CAT4.D)
mAU
4b
lunghezza d'onda = 240nm
min

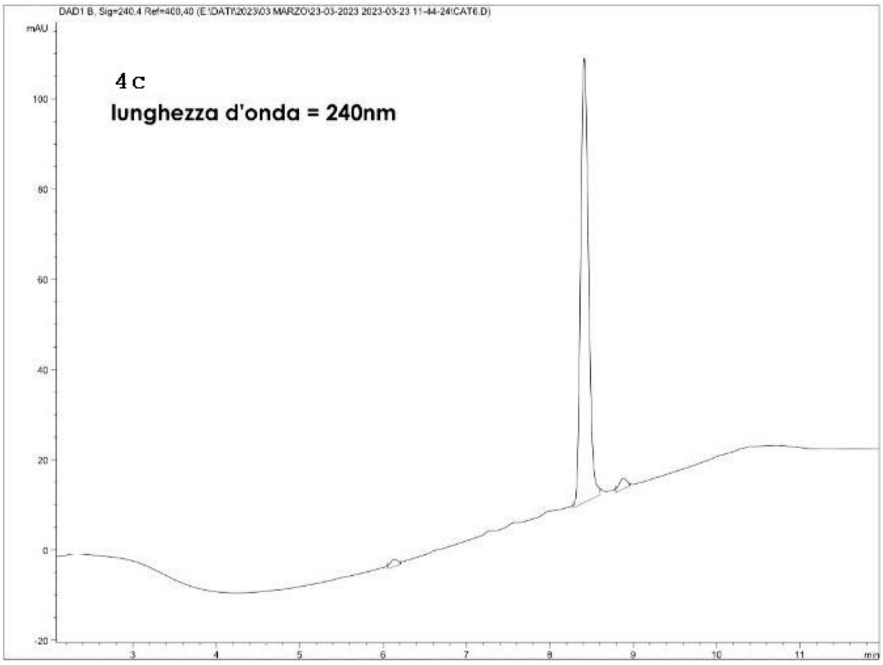
DAD1 B, Sig=240,4 Ref=400,40 (E:\DATI\2023\03 MARZO\23-03-2023 2023-03-23 11-44-24\CAT6.D)
mAU
4c
lunghezza d'onda = 240nm
100
80
60
40
20
0
-20
3
4
5
6
7
8
9
10
11
min

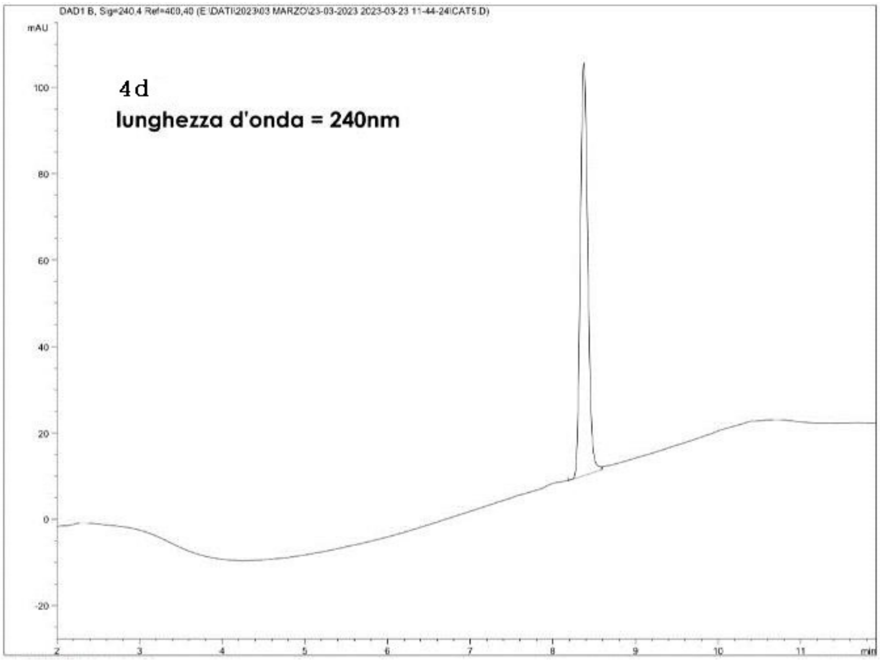
DAD1 B, Sig=240,4 Ref=400,40 (E:\DATI\2023\03 MARZO\23-03-2023 2023-03-23 11-44-24\CAT5.D)
mAU
4d
lunghezza d'onda = 240nm
100
80
60
40
20
0
-20
2
3
4
5
6
7
8
9
10
11
min

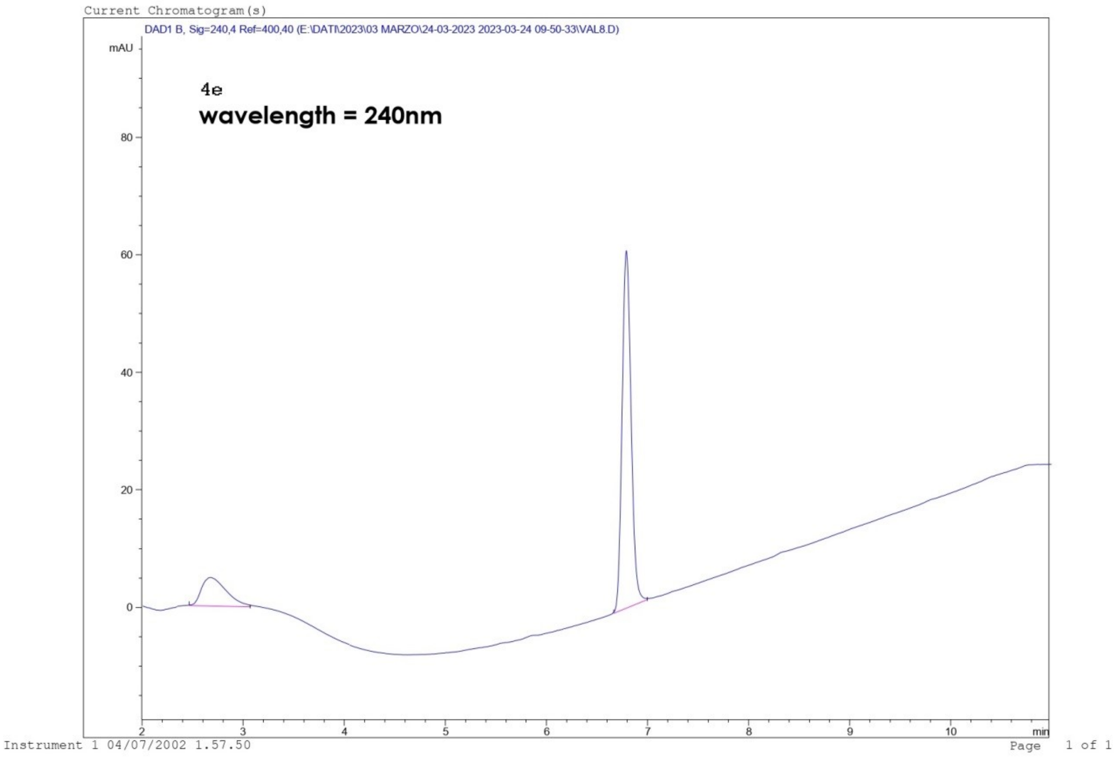
Current Chromatogram(s)
DAD1 B, Sig=240,4 Ref=400,40 (E:\DATI\2023\03 MARZO\24-03-2023 2023-03-24 09-50-33\VAL8.D)
mAU
4e
wavelength = 240nm
min
Instrument 1 04/07/2002 1.57.50
Page 1 of 1

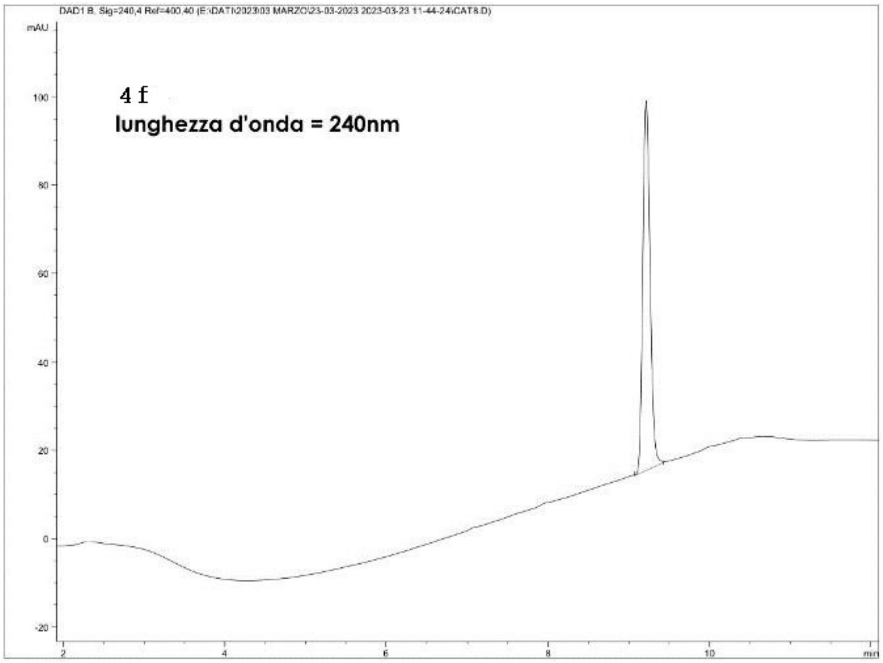
DAD1 B, Sig=240,4 Ref=400,40 (E:\DATI\2023\03 MARZO\23-03-2023 2023-03-23 11-44-24\CAT8.D)
mAU
4 f
lunghezza d'onda = 240nm
min

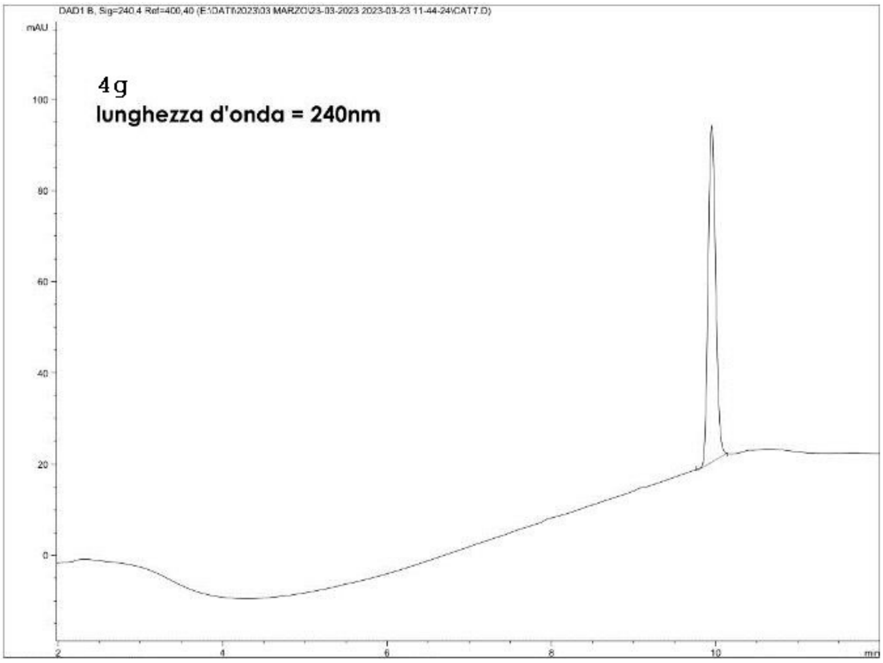
DAD1 B, Sig=240,4 Ref=400,40 (E:\DATI\2023\03 MARZO\23-03-2023 2023-03-23 11-44-24\CAT7.D)
mAU
4g
lunghezza d'onda = 240nm
100
80
60
40
20
0
2
4
6
8
10
min

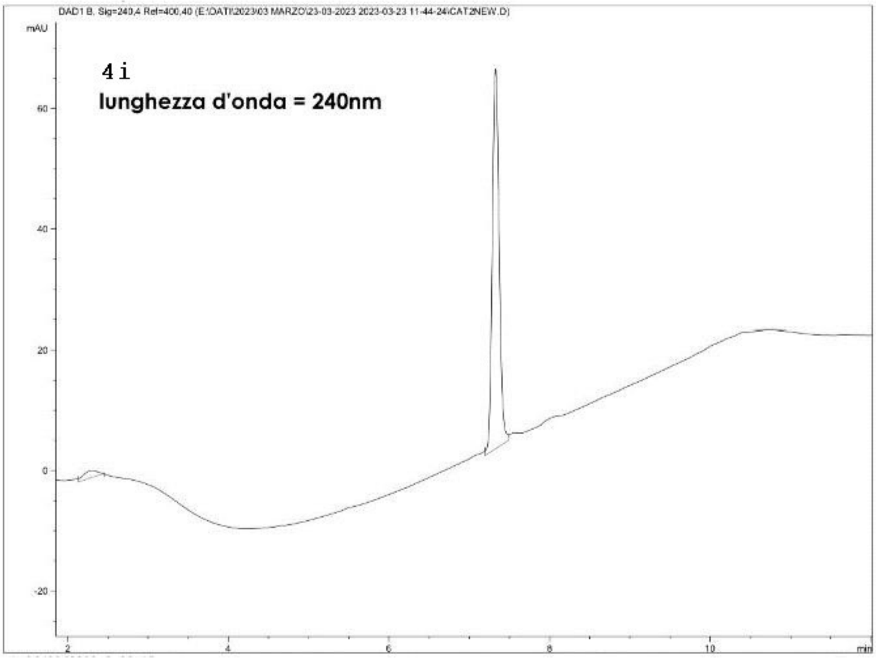
DAD1 B, Sig=240,4 Ref=400,40 (E:\DATI\2023\03 MARZO\23-03-2023 2023-03-23 11-44-24\CAT2NEW.D)
mAU
4i
lunghezza d'onda = 240nm
60
40
20
0
-20
2
4
6
8
10
min

Print of window 38: Current Chromatogram(s)

Current Chromatogram(s)

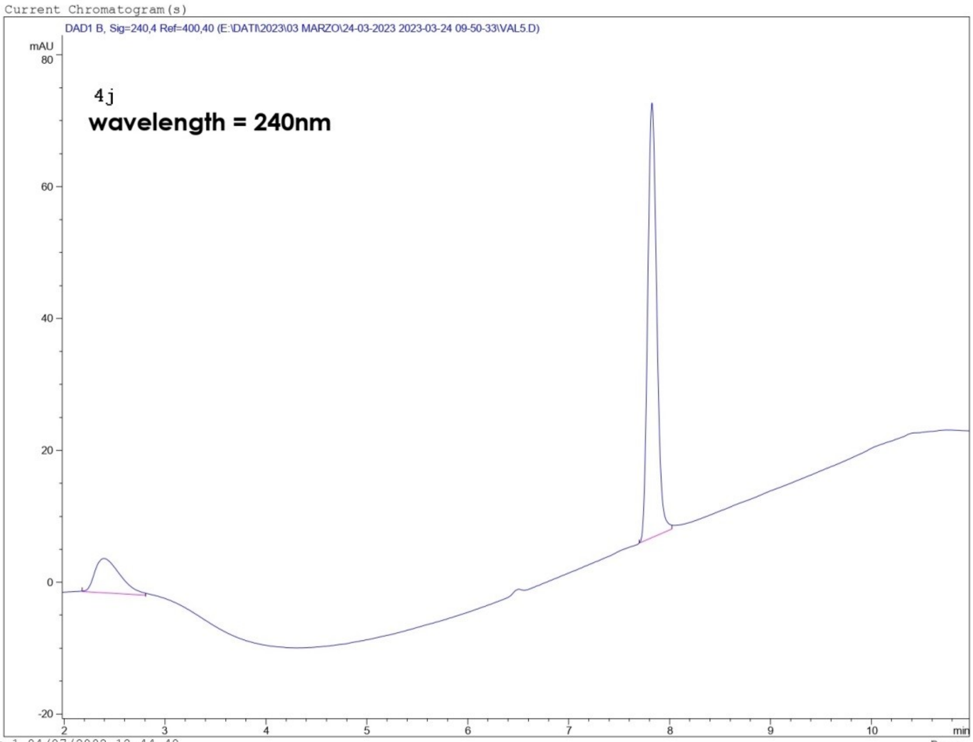


Instrument 1 04/07/2002 12.44.40 Page 1 of 1

Current Chromatogram(s)

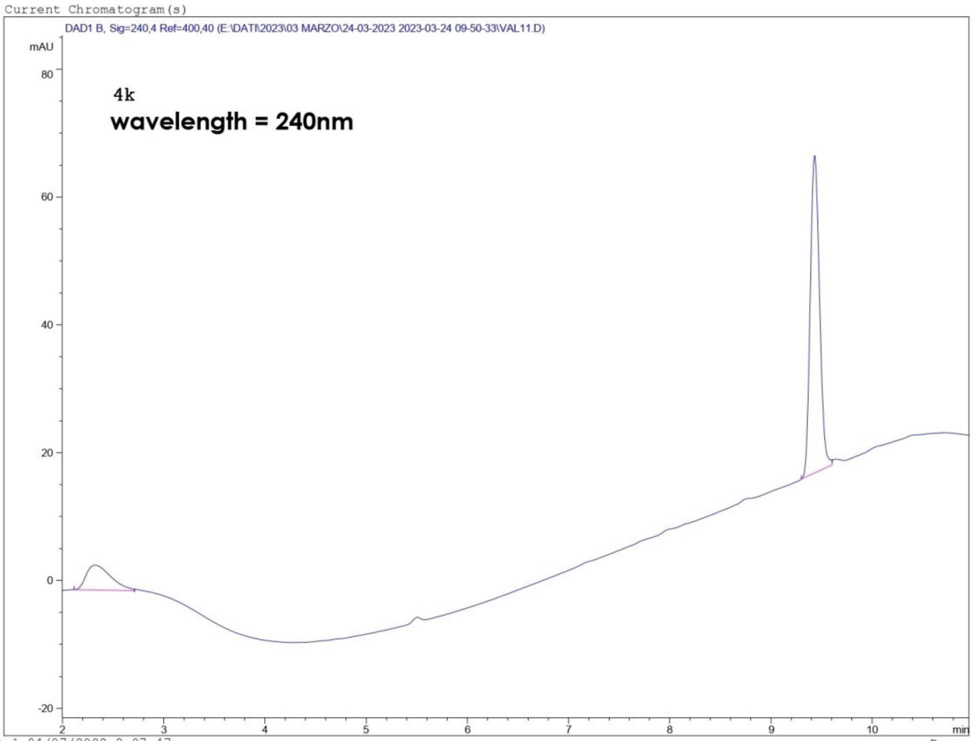


Instrument 1 04/07/2002 2.07.47 Page 1 of 1

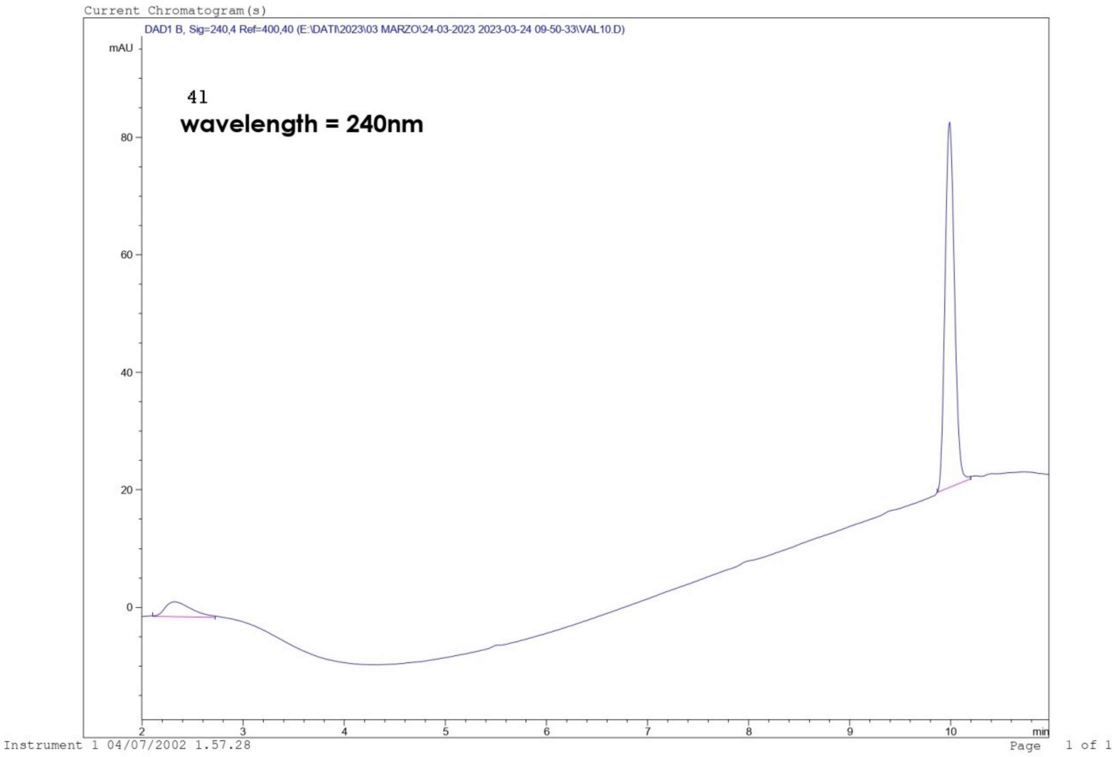
Current Chromatogram(s)
DAD1 B, Sig=240,4 Ref=400,40 (E:\DATI\2023\03 MARZO\24-03-2023 2023-03-24 09-50-33\VAL10.D)
mAU
4l
wavelength = 240nm
80
60
40
20
0
2
3
4
5
6
7
8
9
10
min
Instrument 1 04/07/2002 1.57.28
Page 1 of 1

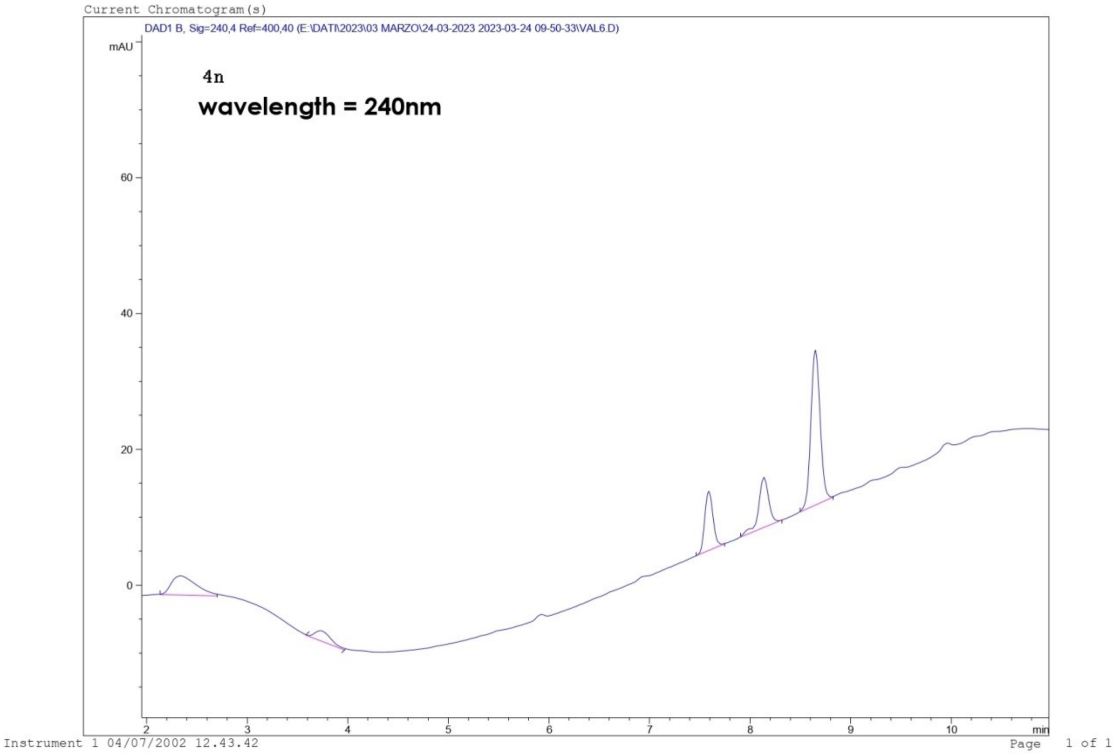
Current Chromatogram(s)
DAD1 B, Sig=240,4 Ref=400,40 (E:\DATI\2023\03 MARZO\24-03-2023 2023-03-24 09-50-33\VAL6.D)
mAU
4n
wavelength = 240nm
60
40
20
0
2
3
4
5
6
7
8
9
10
min
Instrument 1 04/07/2002 12.43.42
Page 1 of 1

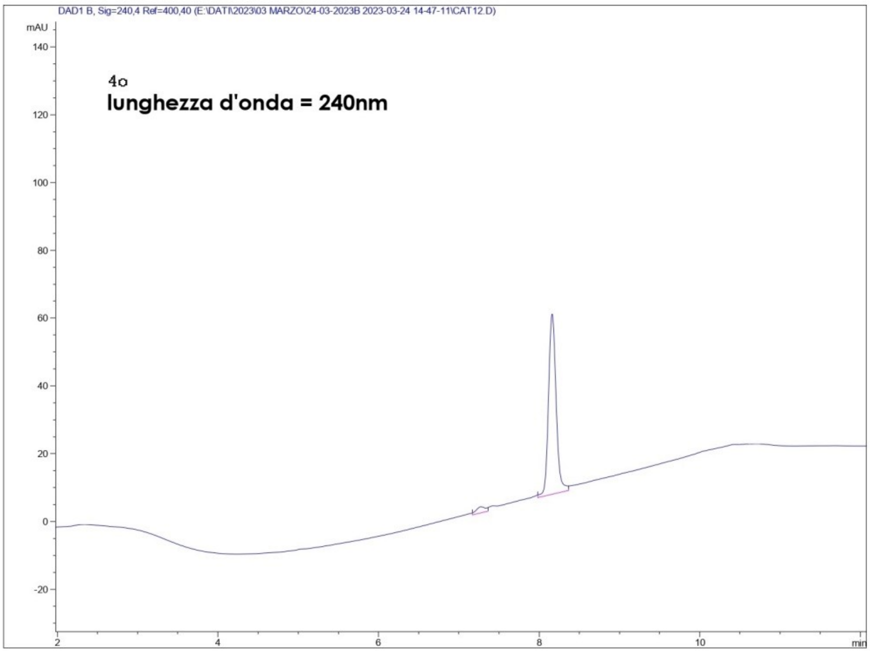


Current Chromatogram(s)

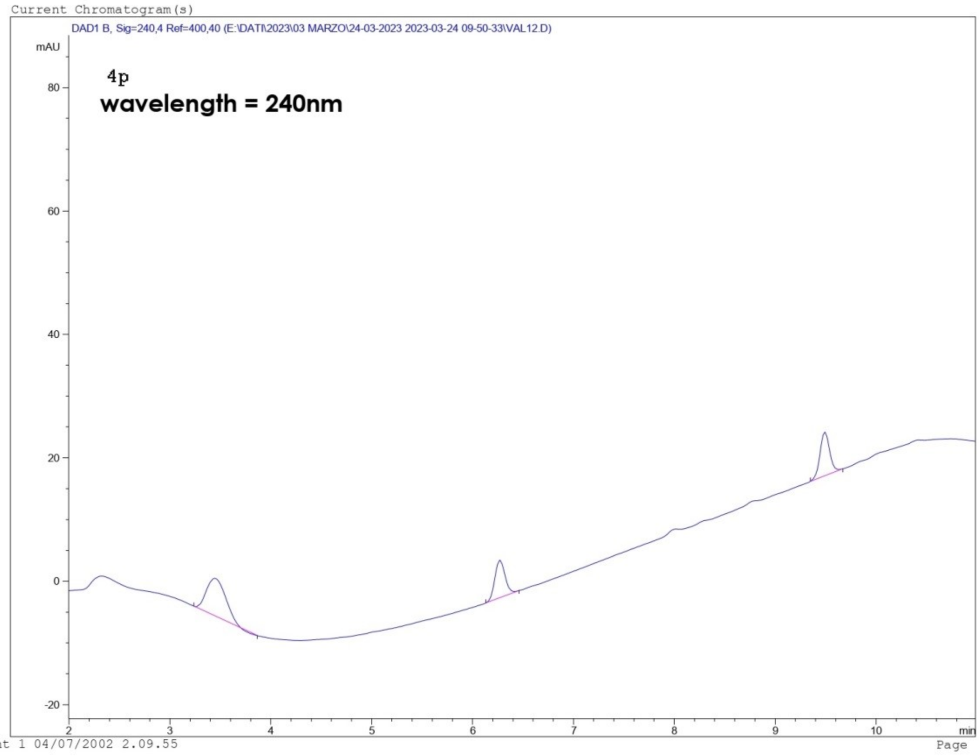


Instrument 1 04/07/2002 2.09.55 Page 1 of 1

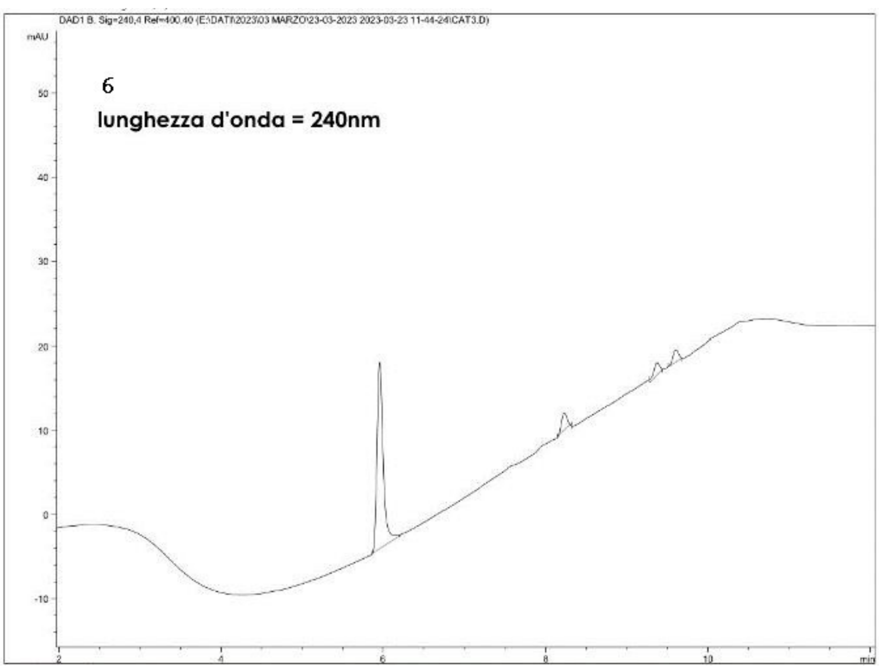
DAD1 B, Sig=240,4 Ref=400,40 (E:\DATI\2023\03 MARZO\23-03-2023 2023-03-23 11-44-24\CAT3.D)
mAU
6
lunghezza d'onda = 240nm
min

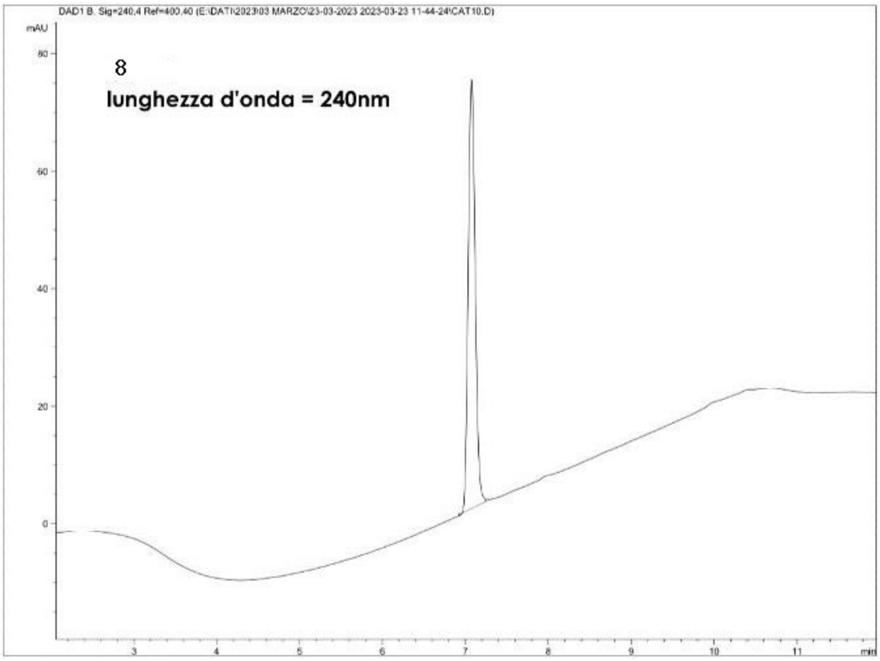
DAD1 B, Sig=240,4 Ref=400,40 (E:\DATI\2023\03 MARZO\23-03-2023 2023-03-23 11-44-24\CAT10.D)
mAU
8
lunghezza d'onda = 240nm
min

**Table S1.** Purity data (retention time RT, area, height, width)

| **Compound** | **RT** (min) | **Area** | **Height** | **Width 1/2** (min) |
|---|---|---|---|---|
| **4b** | 7,535 | 547 | 89 | 0,103 |
| **4c** | 8,412 | 646 | 99 | 0,109 |
| **4d** | 8,374 | 602 | 96 | 0,105 |
| **4f** | 9,212 | 526 | 84 | 0,105 |
| **4g** | 9,948 | 471 | 74 | 0,106 |
| **4i** | 7,326 | 372 | 63 | 0,099 |
| **4m** | 7,120 | 97 | 15 | 0,109 |
| **4o** | 8,160 | 353 | 52 | 0,110 |
| **6** | 5,957 | 116 | 22 | 0,087 |
| **8** | 7,071 | 428 | 73 | 0,098 |

3. *Crystallographic data and refinement parameters of* ***4d***

**Table S2**

| | **4d** |
|---|---|
| empirical formula | $C_{11}H_{11}NO_2Se$ |
| formula weight | 268.17 |
| T (K) | 100 |
| Crystal system, space group | orthorhombic, Pbca |
| Unit cell dimensions (Å) | a = 12.976(1)<br>b = 7.1286(6)<br>c = 22.931(2) |
| $V$ (Å$^3$) | 2121.1(3) |
| Z, dcalc(g/cm$^3$) | 8, 1.680 |
| μ (mm$^{-1}$) | 4.631 |
| F(000) | 1072 |
| Reflections collected/unique/Rint | 16898 / 1920 / 0.0466 |
| Data/paramenters | 1914 / 169 |
| Final R indices [I>2σ(I)] | R1 = 0.0451, wR2 = 0.1271 |
| R indices all data | R1 = 0.0461, wR2 = 0.1274 |

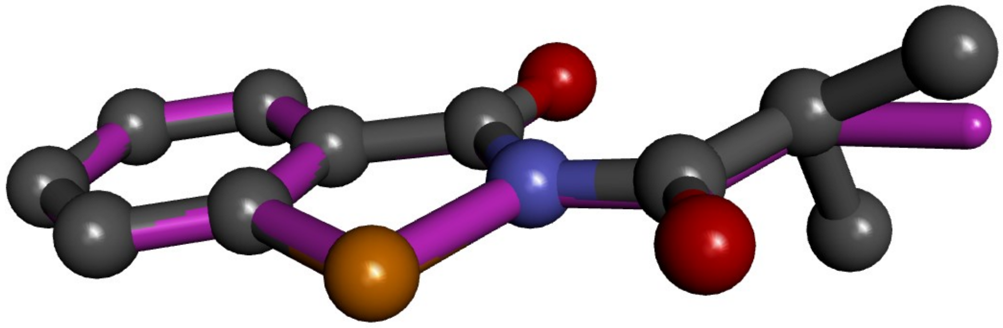

**Figure S1**. Superimposition of the **4d** (ball and sick) and the XAYHIO (stick, purple) molecules.

4. *Volume and surface accessible to solvent for* ***4d****,* ***4e****,* ***4j****,* ***4g*** *and* ***4k*** *and for the i-propyl, t-butyl, c-propyl, n-pentyl and c-pentyl residues*

For molecules **4d, 4e, 4j, 4g** and **4k,** the surface accessible to solvent (Connolly surface, Å$^2$) and the subtended volume (Å$^3$) were evaluated (by Accelrys\DSViewerPro60) and reported in **Table S3.**

The same values are evaluated also for the *i*-propyl, *t*-butyl, *c*-propyl, *n*-pentyl and *c*-pentyl residues.

**Table S3**

| **Compound** | Volume Å$^3$ | Surface area Å$^2$ |
|---|---|---|
| **4d** | 169.96 | 196.66 |
| **4e** | 189.41 | 206.88 |
| **4j** | 161.08 | 187.38 |
| **4g** | 201.88 | 236.45 |
| **4k** | 191.92 | 218.73 |
| | | |
| **residue** | | |
| ***i*-propyle** | 74.57 | 95.55 |
| ***t*-butyl** | 91.66 | 110.31 |
| ***c*-propyl** | 64.77 | 87.77 |
| **n-pentyl** | 107.42 | 133.62 |
| **c-pentyl** | 90.53 | 111.71 |

5. *Overlapping of surface accessible to solvent of compounds* ***4d****,* ***4e****,* ***4j***

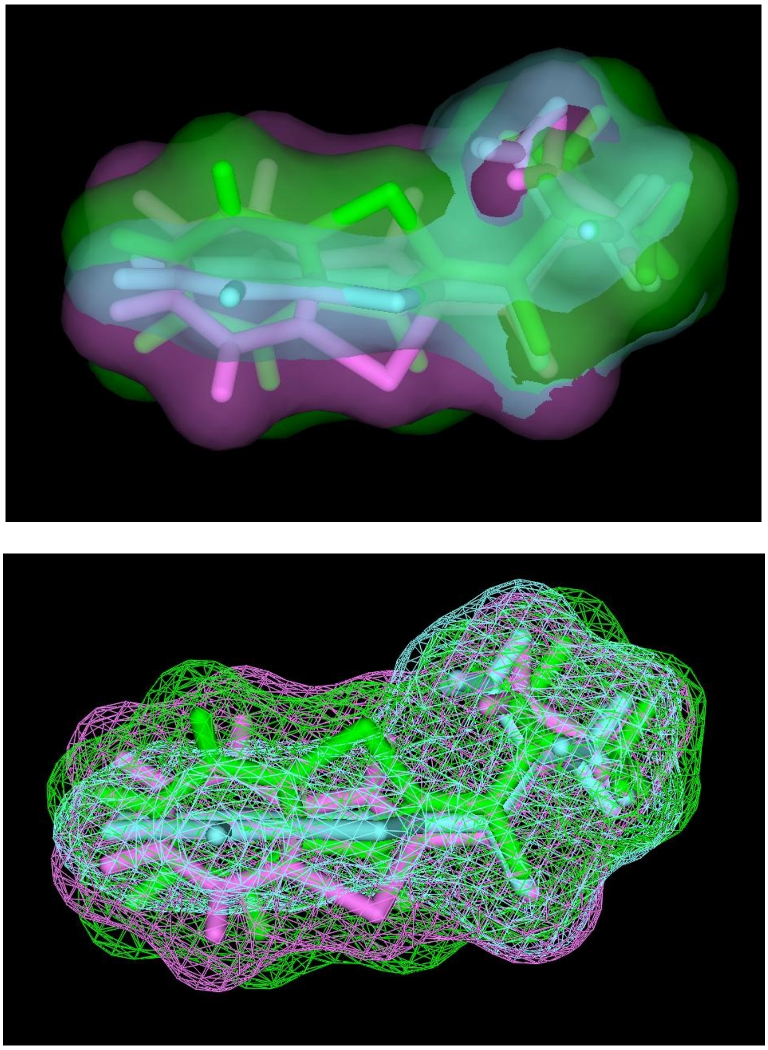

**Figure S2**. The surface accessible to solvent (Connolly surface) of **4d** (green), **4e** (light blue), **4j** (pink).

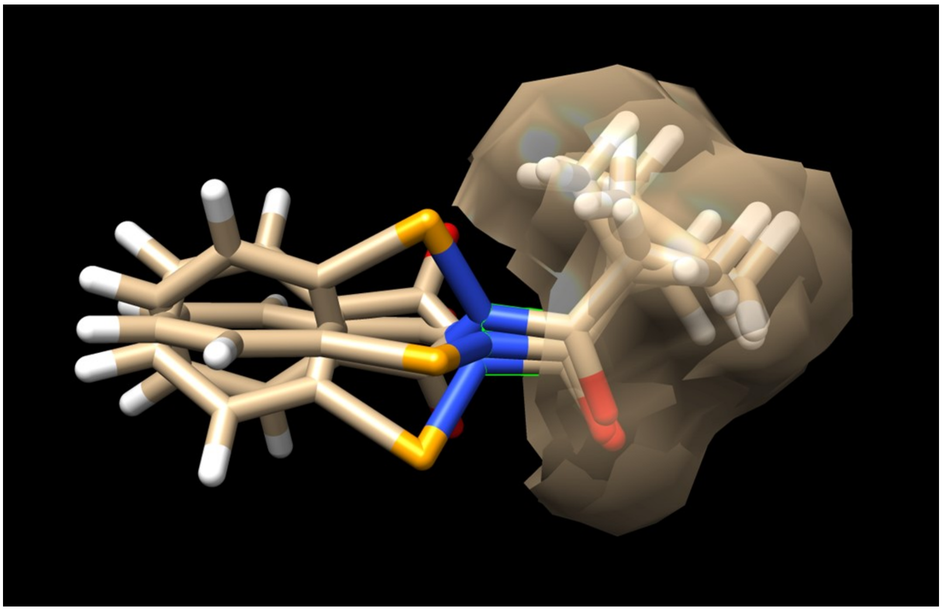

**Figure S3**. Surface accessible to solvent for the residues *i*-propyl, *t*-butyl and *c*-propyl.

Compounds **4g** and **4k**, respectively, the n-pentanyl and the ciclopentanyl derivatives show a similar surface accessible to solvent as illustrated in **Figure S4**.

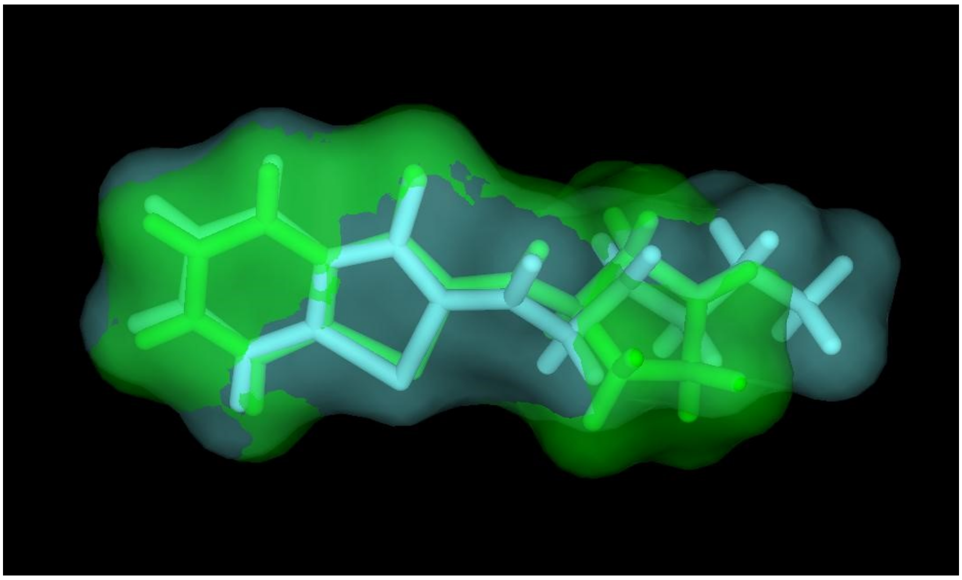

**Figure S4**. The surface accessible to solvent (Connolly surface) of **4g** (light blue) and **4k** (green).

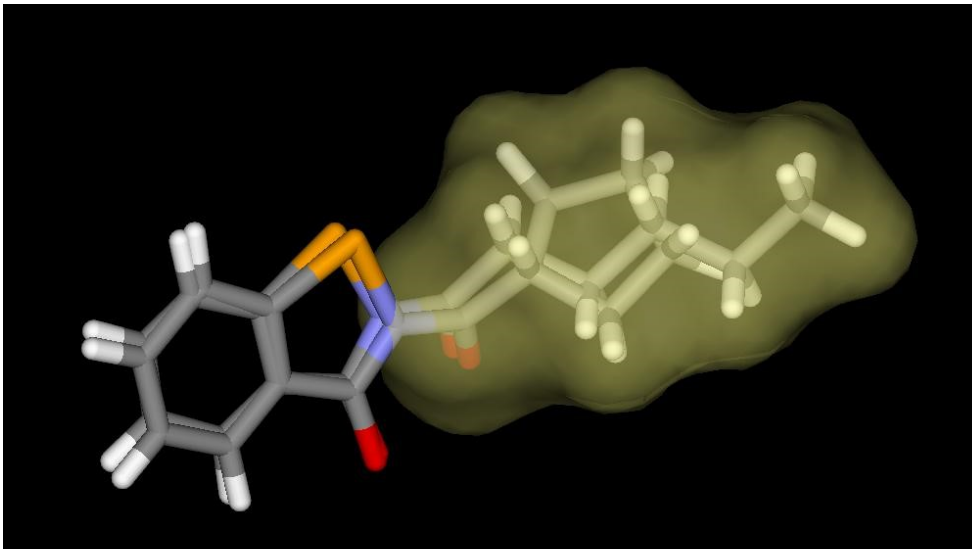

**Figure S5**. Surface accessible to solvent for the residues *n*-pentyl and *c*-pentyl.
